# Transcriptional programs of neoantigen-specific TIL in anti-PD-1-treated lung cancers

**DOI:** 10.1038/s41586-021-03752-4

**Published:** 2021-07-21

**Authors:** Justina X. Caushi, Jiajia Zhang, Zhicheng Ji, Ajay Vaghasia, Boyang Zhang, Emily Han-Chung Hsiue, Brian J. Mog, Wenpin Hou, Sune Justesen, Richard Blosser, Ada Tam, Valsamo Anagnostou, Tricia R. Cottrell, Haidan Guo, Hok Yee Chan, Dipika Singh, Sampriti Thapa, Arbor G. Dykema, Poromendro Burman, Begum Choudhury, Luis Aparicio, Laurene S. Cheung, Mara Lanis, Zineb Belcaid, Margueritta El Asmar, Peter B. Illei, Rulin Wang, Jennifer Meyers, Kornel Schuebel, Anuj Gupta, Alyza Skaist, Sarah Wheelan, Jarushka Naidoo, Kristen A. Marrone, Malcolm Brock, Jinny Ha, Errol L. Bush, Bernard J. Park, Matthew Bott, David R. Jones, Joshua E. Reuss, Victor E. Velculescu, Jamie E. Chaft, Kenneth W. Kinzler, Shibin Zhou, Bert Vogelstein, Janis M. Taube, Matthew D. Hellmann, Julie R. Brahmer, Taha Merghoub, Patrick M. Forde, Srinivasan Yegnasubramanian, Hongkai Ji, Drew M. Pardoll, Kellie N. Smith

**Affiliations:** 1 Bloomberg~Kimmel Institute for Cancer Immunotherapy at Johns Hopkins, Baltimore, MD USA; 2 The Mark Center for Advanced Genomics and Imaging at Johns Hopkins, Baltimore, MD USA; 3 Sidney Kimmel Comprehensive Cancer Center at Johns Hopkins, Baltimore, MD USA; 4 Department of Biostatistics, Bloomberg School of Public Health, Johns Hopkins University, Baltimore, MD USA; 5 Ludwig Center and Howard Hughes Medical Institute at Johns Hopkins, Baltimore, MD USA; 6 Lustgarten Pancreatic Cancer Research Laboratory, Sidney Kimmel Comprehensive Cancer Center, Johns Hopkins University, Baltimore, MD USA; 7 Immunitrack, Copenhagen, Denmark; 8 Department of Medicine, Memorial Sloan Kettering Cancer Center and Weill Cornell Medicine, New York, NY USA; 9 The Swim Across America and Ludwig Collaborative Laboratory, Immunology Program, Parker Institute for Cancer Immunotherapy at Memorial Sloan Kettering Cancer Center, New York, NY USA; 10 Human Oncology and Pathogenesis Program, Memorial Sloan Kettering Cancer Center, New York, NY USA; 11 Present Address: Department of Biostatistics and Bioinformatics, Duke University School of Medicine, Durham, NC USA; 12 Present Address: Ontario Institute for Cancer Research, Queens University, Kingston, Ontario Canada; 13 Present Address: Beaumont Hospital Dublin, RCSI University of Medicine and Health Science, Dublin, Ireland; 14 Present Address: Georgetown Lombardi Comprehensive Cancer Center at Georgetown University, Washington, DC USA

**Keywords:** Cellular immunity, Immunotherapy, CD8-positive T cells, T-cell receptor, Tumour immunology

## Abstract

PD-1 blockade unleashes CD8 T cells^1^, including those specific for mutation-associated neoantigens (MANA), but factors in the tumour microenvironment can inhibit these T cell responses. Single-cell transcriptomics have revealed global T cell dysfunction programs in tumour-infiltrating lymphocytes (TIL). However, the majority of TIL do not recognize tumour antigens^2^, and little is known about transcriptional programs of MANA-specific TIL. Here, we identify MANA-specific T cell clones using the MANA functional expansion of specific T cells assay^3^ in neoadjuvant anti-PD-1-treated non-small cell lung cancers (NSCLC). We use their T cell receptors as a ‘barcode’ to track and analyse their transcriptional programs in the tumour microenvironment using coupled single-cell RNA sequencing and T cell receptor sequencing. We find both MANA- and virus-specific clones in TIL, regardless of response, and MANA-, influenza- and Epstein–Barr virus-specific TIL each have unique transcriptional programs. Despite exposure to cognate antigen, MANA-specific TIL express an incompletely activated cytolytic program. MANA-specific CD8 T cells have hallmark transcriptional programs of tissue-resident memory (TRM) cells, but low levels of interleukin-7 receptor (IL-7R) and are functionally less responsive to interleukin-7 (IL-7) compared with influenza-specific TRM cells. Compared with those from responding tumours, MANA-specific clones from non-responding tumours express T cell receptors with markedly lower ligand-dependent signalling, are largely confined to HOBIT^high^ TRM subsets, and coordinately upregulate checkpoints, killer inhibitory receptors and inhibitors of T cell activation. These findings provide important insights for overcoming resistance to PD-1 blockade.

## Abstract (web summary)

Single-cell RNA sequencing and T cell receptor sequencing are combined to identify transcriptional programs specific to mutation-associated neoantigen-specific T cells in non-small cell lung cancers treated with anti-PD-1, providing insights into resistance to PD-1 blockade.

## Main

The efficacy of PD-1- and PD-L1-blocking agents is predicated upon CD8 T cell-mediated anti-tumour immunity^1^. Early studies focused on tumour-associated antigens, whereas recent work has shifted attention to T cell recognition of mutation-associated neoantigens (MANA), owing to the large numbers of somatic mutations acquired by many cancers during their development^4^. The association of improved anti-PD-1 and anti-PD-L1 clinical responses with high tumour mutational burden^5^ strongly suggests that MANA are important targets of anti-tumour immunity induced by PD-1 blockade.

Despite the success of immune checkpoint blockade (ICB) in improving clinical outcomes, most cancers still do not respond^6^. Improving response rates to ICB will require an understanding of the functional state of tumour-specific T cells, particularly in the tumour microenvironment. However, a fundamental limitation in the current understanding of the T cell functional programs that underpin the response to ICB has been the absence of transcriptional profiling of true MANA-specific TIL. A related problem is the paucity of information regarding the differences between MANA-specific TIL in ICB-responsive versus resistant tumours. Indeed, MANA-specific T cells represent a small fraction of total TIL^2,7^, particularly in lung cancer, in which they have been shown to selectively upregulate CD39. This highlights the challenges confronting characterization of the cells responsible for the activity of T cell-targeting immunotherapies.

## Global gene expression of NSCLC TIL

**Fig. 1:**
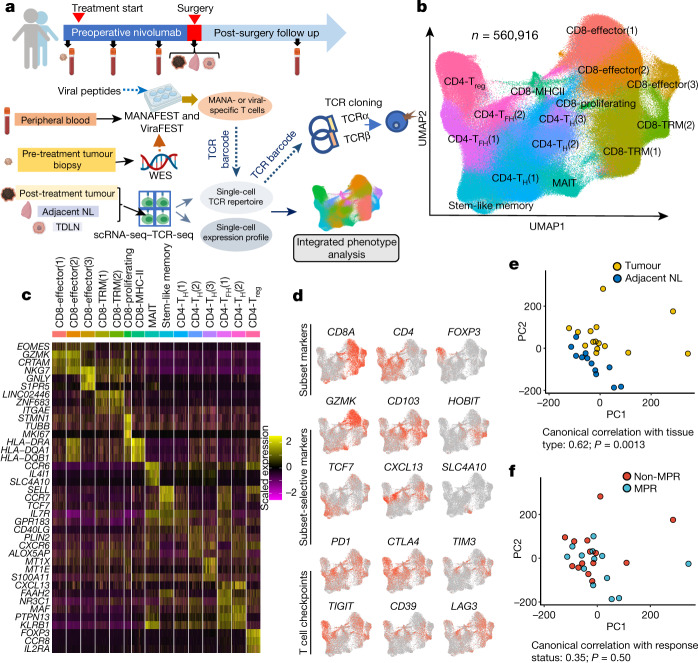
Profiling single T cells in NSCLC treated with neoadjuvant PD-1 blockade. Twenty patients with resectable NSCLC were treated with two doses of PD-1 blockade before surgical resection. **a**, An overall schematic of the clinical trial, biospecimen collection (top) and study design (bottom). scRNA-seq–TCR-seq was performed on T cells isolated from resected tumour (*n* = 15), adjacent normal lung (NL; *n* = 12), TDLN (*n* = 3), and a resected brain metastasis (*n* = 1) from patients with NSCLC treated with two doses of neoadjuvant anti-PD-1 (bottom). The MANAFEST and ViraFEST assays were used to identify MANA- and viral (EBV and influenza)-specific TCRs, respectively. WES, whole-exome sequencing. **b**, UMAP projection of the expression profiles of the 560,916 T cells that passed quality control. Immune cell subsets, defined by 15 unique clusters, are annotated and marked by colour code. **c**, Relative expression of the top-3 most differentially expressed genes. Five-thousand cells (or all cells in the cluster if the cluster size was fewer than 5,000 cells) were randomly sampled from each cluster for visualization. MAIT, mucosal-associated invariant T cells; T_FH_, T follicular helper cells; T_reg_, regulatory T cells. **d**, Expression of T cell subset-defining genes, T cell subset-selective genes and major T cell checkpoint genes. *CD39* is also known as *ENTPD1*. **e**, PCA of cell-cluster-level pseudobulk gene expression for individual samples for tumour (yellow, *n* = 15) and adjacent normal lung (dark blue, *n* = 12). One-sided permutation test. **f**, PCA of cell-cluster-level pseudobulk gene expression for non-MPR (red, *n* = 9) and MPR (light blue, *n* = 6) tumours. One-sided permutation test.

**Extended Data Fig. 1:**
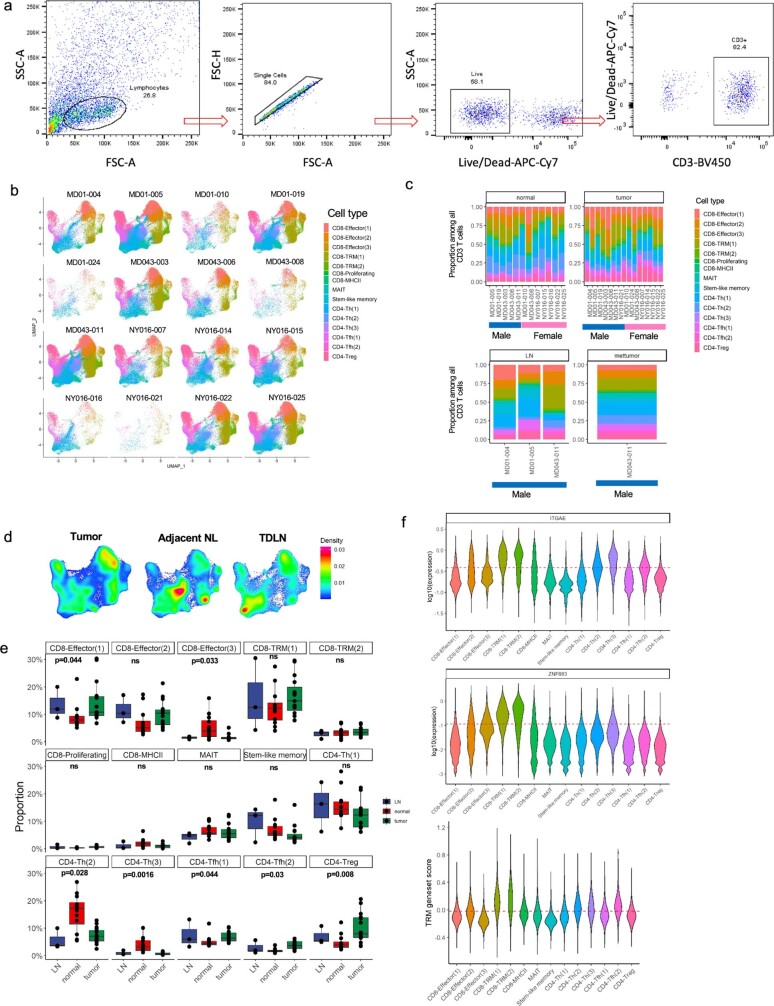
Defining CD3^+^ T cell subsets in patients with non-small cell cancer treated with anti-PD-1. **a**, FACS gating strategy for sorting CD3+ T cells. The gating strategy is shown for sorting live CD3^+^ T cells from tumour, normal lung, lymph node, or metastasis, when available, on a BD FACSAria. **b**, Patient and tissue compartment variability across clusters on UMAP. scRNA-seq–TCR-seq was performed on available resected biospecimens (tumour, adjacent NL, TDLN, and a brain metastasis) from 16 patients treated with neoadjuvant PD-1 blockade. CD3^+^ T cells stratified by patient are visualized using UMAP. Each cluster is annotated and marked by colour code. **c**, Barplots show the proportion of each T cell cluster in the TDLN, brain metastasis, tumour, and adjacent NL of each patient. Each cluster as shown on the UMAP is denoted by colour code. No clusters were driven by a particular patient based. **d**, A density plot of all CD3^+^ T cells on the UMAP, stratified by tissue compartment, is shown. Cells were obtained from 15 tumours, 12 adjacent NL specimens, and 3 TDLN. Because a metastasis was sequenced in only one patient, this specimen is not included in this analysis. **e**, The proportion (%) of total CD3^+^ T cells made up by each T cell cluster was compared between tumour (*n* = 15 biologically independent samples), adjacent NL (*n* = 12 biologically independent samples), and TDLN (*n* = 3 biologically independent samples). *P* values were obtained using Kruskal–Wallis Test and were adjusted for multiple comparisons using Benjamini-Hochberg method. Each dot represents a patient and all data points are shown. Individual data points are superimposed over a Box and Whiskers plot summarizing the data. The middle bar shows the median, with the lower and upper hinges corresponding to the 25^th^ and 75^th^ percentiles, respectively (interquartile range, IQR). The upper whisker extends from the hinge to the largest value no further than 1.5 * IQR from the hinge. The lower whisker extends from the hinge to the smallest value at most 1.5 * IQR of the hinge. **f**, Tissue-resident defining genes and core TRM gene set signature on different T cell cluster. The top and middle violin plots show the expression of TRM-defining genes (ITGAE, ZNF683) by each cell in each cluster. The dashed line indicates the mean expression of the respective gene among all CD3^+^ T cells. Expression values were log_10_ transformed for visualization. The bottom violin plot shows the TRM gene-set score for each cluster. This gene-set is comprised of TRM-associated genes as published previously (Supplementary Data 2.1). The dashed line shows the mean TRM gene-set score among all T cells. Because the proliferating cluster is driven by proliferation-associated genes and is comprised of mixed cell types, this cluster was not shown in the violin plots.

For this study, we used peripheral blood and tissue biospecimens obtained from the first-in-human clinical trial of neoadjuvant anti-PD-1 (nivolumab) in resectable non-small cell lung cancer^8^ (NSCLC; ClinicalTrials.gov identifier: NCT02259621; Fig. 1a, top) to study the transcriptional programs of MANA-specific TIL. Nine out of 20 patients with NSCLC (45%) treated in this trial had a major pathologic response (MPR) at the time of resection, defined as no more than 10% viable tumour at the time of surgery; previous studies have established an association between MPR and improved overall survival^9–12^. A schematic of the study design and experimental approach is shown in Fig. 1a, bottom. Combined single-cell RNA sequencing (scRNA-seq) and T cell receptor sequencing (TCR-seq) was performed on TIL (*n* = 15), paired adjacent normal lung (*n* = 12), tumour-draining lymph nodes (TDLN, *n* = 3) and a distant metastasis (Extended Data Fig. 1a, Supplementary Tables 1–3). In total, 560,916 T cells passed quality control (Fig. 1b, Supplementary Table 3) and were carried forward for analyses.

Uniform manifold approximation and projection (UMAP) analysis of cells from all samples on the basis of filtered and normalized transcript counts defined 15 T cell clusters (Fig. 1b, c, Extended Data Fig. 1b–e, Supplementary Data 1.1). Expression of subset-defining markers and T cell checkpoints was visualized in red scale on the UMAP (Fig. 1d). The two clusters designated as TRM had the highest expression of the canonical TRM genes, *ZNF683* (also known as *HOBIT*) and *ITGAE* (also known as *CD103*), and the highest expression of a TRM gene set^13^ (Extended Data Fig. 1f, Supplementary Data 2.1). Principal component analysis (PCA) of samples based on concatenated cell-cluster-level pseudobulk profiles distinguished adjacent normal-lung T cells from TIL (Fig. 1e), but did not distinguish MPR from non-MPR TIL (Fig. 1f). We did not observe notable differentially expressed gene programs between MPR and non-MPR TIL (Supplementary Data 3), indicating that gene expression profiling of total TIL has limited sensitivity in distinguishing the pathologic response to PD-1 blockade.

## Expression programs of MANA-specific TIL

**Fig. 2:**
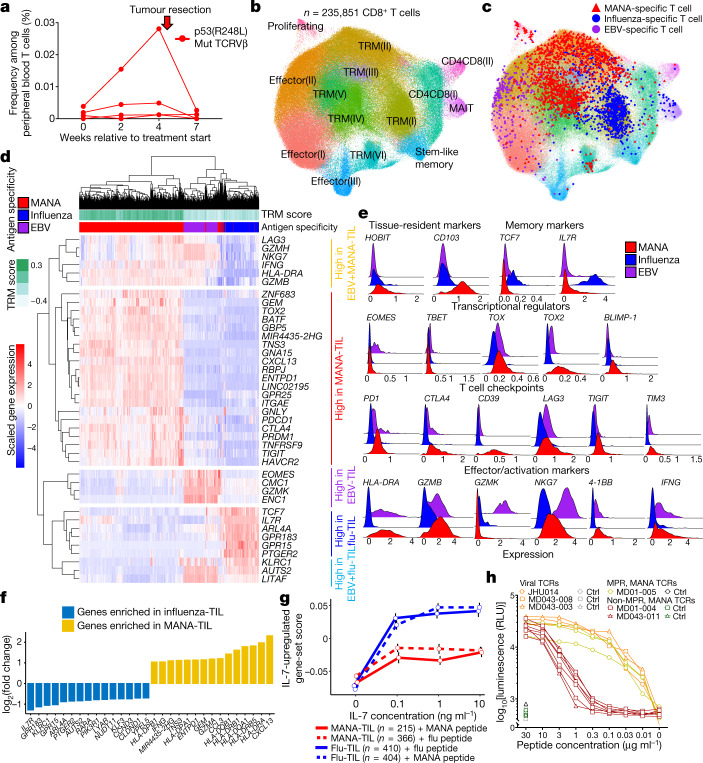
Characterization of antigen-specific T cells in NSCLC treated with neoadjuvant PD-1 blockade. The MANAFEST assay was performed on four patients with MPR and five patients without MPR. Results are shown in Extended Data Fig. 2 and Supplementary Data 5. **a**, Four TCRs recognizing p53(R248L)-derived MD01-004-MANA12 were identified in patient without MPR MD01-004. Their frequency was tracked in serial peripheral blood. Mut, mutant. **b**, Refined clustering was performed on 235,851 CD8^+^ T cells from tumour (*n* = 15), adjacent normal lung (*n* = 12), TDLN (*n* = 3) and one resected brain metastasis (MD043-011). Fourteen unique clusters were visualized and were using T cell gene programs described in previous studies^16^. Cluster-defining genes are shown in Extended Data Fig. 5a. **c**, MANA-specific (red), influenza-specific (blue) and EBV-specific (purple) clonotypes were visualized on the CD8 UMAP. **d**, Antigen-specific gene programs in the TIL were visualized as a heat map. Comparisons were performed at the individual cell level using a two-sided Wilcoxon rank-sum test with *P*-value adjustment using Bonferroni correction. **e**, Expression levels of key markers are shown. *TBET* is also known as *TBX21*; *4-1BB* is also known as *TNFRSF9*. **f**, Transcriptional programs of influenza-specific and MANA-specific TIL were compared. The top-15 significantly upregulated genes in influenza -specific T cells (blue) and in MANA-specific T cells (yellow) are shown. **g**, TIL from MD01-004 were cultured with MD01-004-MANA-12 or influenza peptide and titrating concentrations of IL-7, followed by scRNA-seq–TCR-seq. In total, 814 influenza-specific TIL (410 co-cultured with influenza peptide and 404 co-cultured with MANA peptide) and 581 MANA-specific TIL (366 co-cultured with influenza peptide and 215 co-cultured with MANA peptide) were detected from a single experiment and were analysed. Composite expression of an IL-7 gene set by influenza-specific and MANA-specific TIL (as determined by their TCRVβ CDR3) was analysed. Dose–response curve of the IL-7-upregulated gene set-score is shown (mean ± s.e.m.). **h**, TCRs corresponding to seven MANA-specific clonotypes from two patients without MPR (red lines), three MANA-specific clonotypes from a patient with MPR (yellow lines), two influenza-specific TCRs, and one EBV-specific TCR (orange lines) were tested for ligand-dependent TCR-signalling capacity. Ctrl, control; RLU, relative luminescence units.

**Extended Data Fig. 2:**
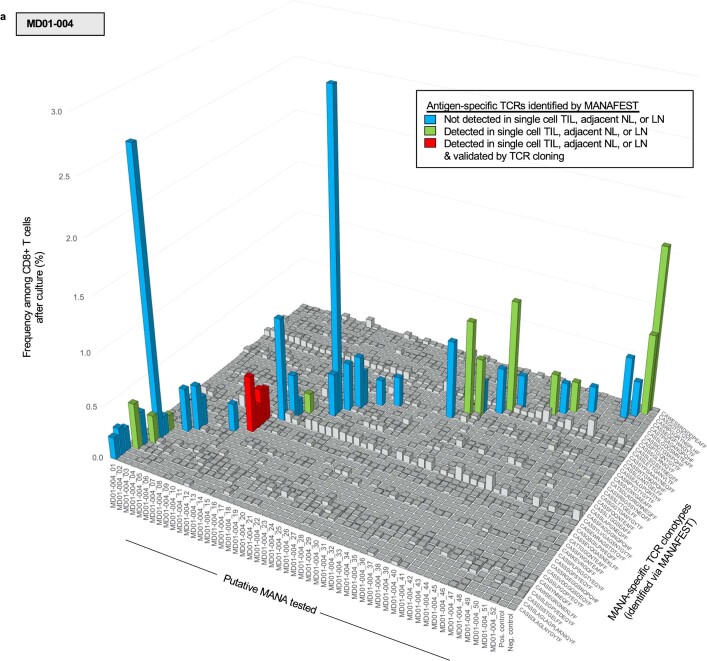
MANA-specific TCRs detected in patient without MPR MD01-004 using MANAFEST and ViraFEST assays. Antigen-specific responses identified using the MANAFEST assay are shown for patient without MPR MD01-004. MANAFEST assays for all other patients are shown in Supplementary Data 5. Each antigen-specific clonotypic expansion is colour coded to indicate if the clone was not detected in the single-cell data (blue), detected in the single-cell data but not tested via TCR cloning (green), or detected in the single-cell data and validated with TCR cloning (red). Data are shown as the percent of MANAFEST+ clonotypes among CD8+ T cells after 10 day culture.

**Extended Data Fig. 3:**
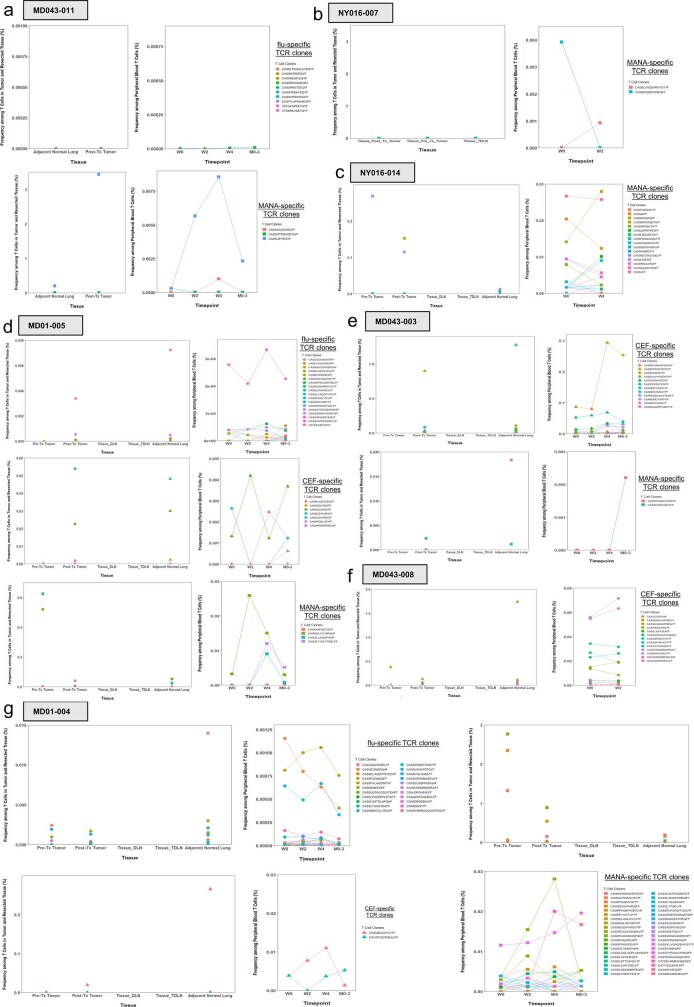
Peripheral dynamics and cross-compartment representation of antigen-specific T cells. Bulk TCRseq was performed on pre- and post-treatment tissue (left panels) and peripheral blood (right panels) for each patient in whom antigen-specific TCRs were identified by ViraFEST/MANAFEST (as shown in Extended Data Fig. 2 and Supplementary Data 5). Data are shown as the frequency of each influenza-, CEF-, and MANA-specific TCR clonotype among all TCRs detected by bulk TCR sequencing of the indicated tissue or peripheral blood time point. Antigen-specific clonotypes were not detected by bulk TCRseq of any available tissue/peripheral blood time point in patient NY016-025. TDLN, tumour draining lymph node; DLN, draining lymph node.

We next performed the MANA functional expansion of specific T cells assay (MANAFEST)^3^ on 9 of the 16 individuals on whom scRNA-seq–TCR-seq was conducted. This assay detects in vivo antigen-experienced T cell responses and identifies the clonal identity of the T cell receptor (TCR) corresponding to these cells. Of these nine, four were classed as MPR and five were non-MPR (results from one individual have been previously described^8^). Putative MANA (Supplementary Tables 4–6), peptide pools representing influenza matrix and nucleoproteins, and a pool of major histocompatibility complex (MHC) class I-restricted cytomegalovirus (CMV), Epstein–Barr virus (EBV) and influenza virus epitopes (CEF) were queried for CD8^+^ T cell reactivity in parallel (Supplementary Tables 6, 7). From 7 (3 with MPR and 4 without MPR) of the 9 individuals, 72 total unique MANA-specific TCRs, 33 unique CEF-specific TCRs, and 52 unique influenza-specific TCRs were identified (Extended Data Fig. 2, Supplementary Tables 8, 9, Supplementary Data 4, 5). Out of 33 CEF-specific TCRs, 6 matched known public EBV-specific TCRs and 3 matched known public influenza-specific TCRs^14^. No CMV-reactive TCRs were mapped from our CEF-specific TCRs. Notably, 4 of the 41 MANA-specific TCRVβ complementarity-determining region 3 (CDR3) clonotypes identified in a patient without MPR (patient ID MD01-004) (Extended Data Fig. 2) were specific for a MANA (MD01-004-MANA12) derived from a p53 R248L hotspot mutation, and were found at appreciable frequency in the pre- and post-treatment tumour (Extended Data Fig. 3), despite the tumour not attaining MPR. Most MANA-specific clones were detected at very low frequency (median: 0.001%) in the peripheral blood across all available time points (Fig. 2a, Extended Data Fig. 3). Overall, pathologic response was not associated with the prevalence, frequency or intratumoral representation of MANA-specific T cells (Extended Data Figs. 2, 3 Supplementary Table 9). In fact, more MANA-specific TIL were observed among non-MPR TIL than among MPR TIL. No consistent pattern was observed for the frequency of viral-specific T cells in the tissue or peripheral blood (Extended Data Figs. 2, 3).

**Extended Data Fig. 4:**
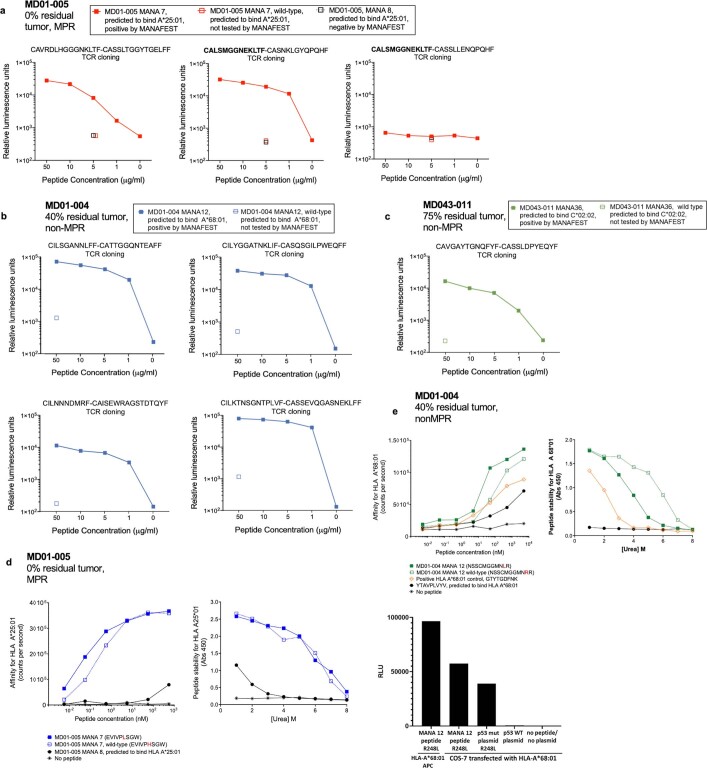
TCR cloning validation of MANA-specific TCRs and MANA binding kinetics. Ten TCRs identified via the MANAFEST assay were selected for TCR cloning and transfer into our NFAT/luciferase Jurkat reporter system. Seven of these TCRs recognized the cognate MANA. **a**, In MD01-005, three TCR Vβ clonotypes recognizing the ARVCF H497L-derived EVIVPLSGW MANA were identified by MANAFEST. Single-cell analysis determined that the Vβ CDR3 s CASNKLGYQPQHF and CASSLLENQPQHF were consistently detected in the same cell and paired with the same Vα CDR3, CALSMGGNEKLTF, likely the result of incomplete allelic exclusive at the beta locus. To validated that these TCRs recognized MD01-005-MANA7, and to determine which Vβ CDR3 was responsible for recognition in the case of incomplete allelic exclusion, all three TCRs were cloned into the Jurkat NFAT luciferase reporter system and tested against autologous LCL loaded with titrating concentrations of MD01-005-MANA7. Data are shown as relative luminescence units (RLU) for MD01-005-MANA7 (solid red square), the cognate wild-type peptide (open red square), or MD01-005-MANA8, which was predicted to bind A*25:01, for each individual TCR. **b**, In patient without MPR MD01-004, four TCRs recognizing the p53 R248L-derived NSSCMGGMNLR MANA (MD01-004-MANA12) were identified by MANAFEST and were detected in the single-cell data. Each Vβ chain paired exclusively with a single Vα chain. These four TCRs were cloned into the Jurkat NFAT/luciferase reporter system and tested against autologous LCL loaded with titrating concentrations of MD01-004-MANA12. Data are shown as relatively luminescence units (RLU) in response to MD01-004-MANA12 (solid blue square) or the cognate wild-type peptide (open blue square). **c**, In patient without MPR MD043-011, a TCR recognizing the CARM1 R208W-derived FAAQAGAWKIY MANA (MD043-011-MANA36) was a candidate for positivity by MANAFEST and was detected in the single-cell data. This Vβ chain paired exclusively with a single Vα chain. This TCR was cloned into the Jurkat NFAT/luciferase reporter system and tested against autologous LCL loaded with titrating concentrations of MD043-011-MANA36. Data are shown as relatively luminescence units (RLU) in response to MD01-004-MANA12 (solid green square) or the cognate wild-type peptide (open green square). **d**, The affinity of MD01-005-MANA7 for HLA A*25:01 was assessed using a luminescent oxygen channeling immunoassay (LOCI, left). This is a proximity-based system using a “donor” and “acceptor” bead, each conjugated with an epitope tag. When the donor bead is excited with light at 650nm and can activate an acceptor bead, resulting in a signal at 520-620nm, which can be quantified per second as a surrogate of affinity. A higher number of counts per second indicates higher affinity of the peptide:HLA pair. Data are shown as the number of counts per second for titrating concentrations of MD01-005-MANA7 (solid blue square), the cognate wild-type (open blue square), MD01-005-MANA8, which is predicted to bind HLA A*68:01 (black circle), or no peptide (star). Stability of these same peptides in the HLA A*68:01 complex was also evaluated using a urea-based assay, whereby the stability of the peptide:HLA complex is measured at increasing concentrations of urea (right). Data are shown as the absorbance at 450nm. Data points represent the mean +/− s.d. of two independent experiments. **e**, Binding (top left) and stability (top right) assays were conducted as in (b) for the p53 R248L-derived MD01-004-MANA12 (solid green square), the cognate wild-type peptide (open green square), a positive control peptide for HLA A*68:01 (orange diamond), the YTAVPLVYV peptide which is predicted to bind A*68:01 (black circle), or no peptide (black star). Data points represent the mean +/− s.d. of two independent experiments. To determine if MD01-004-MANA12 is endogenously processed and presented by HLA A*68:01, COS-7 cells were transfected with HLA-A*68:01 plasmid and p53 R248L mutant plasmid or p53 wild type plasmid. HLA- and p53-transfected COS-7 cells, autologous APC loaded with MD01-004-MANA12, and HLA-A*68:01-transfected COS-7 were co-cultured with CD8+ Jurkat reporter cells expressing the MD01-004-MANA12-reactive TCR, Vβ: CATTGGQNTEAFF, V𝛼: CILSGANNLFF. Data are shown as relative luminescence units (RLU) for each condition (bottom).

Ten MANA-specific clonotypes, for which the TCRα could be confidently identified from the single-cell analysis, were selected for validation of MANA recognition via TCR cloning and introduction into a Jurkat–NFAT luciferase reporter system^15^. Seventy per cent of tested clonotypes (representing 95.2% of total cells bearing TCRs identified by MANAFEST) were validated as MANA-specific (Extended Data Fig. 4a–c). Peptide–human leukocyte antigen (HLA) binding assays demonstrated that two MANA peptides—MD01-005-MANA7 and MD01-004-MANA12—displayed comparably high MHC class I affinity (measured dissociation constants (*K*_d_) = 5.1 nM and 17.5 nM, respectively) and stability (Extended Data Fig. 4d, e).

**Extended Data Fig. 5:**
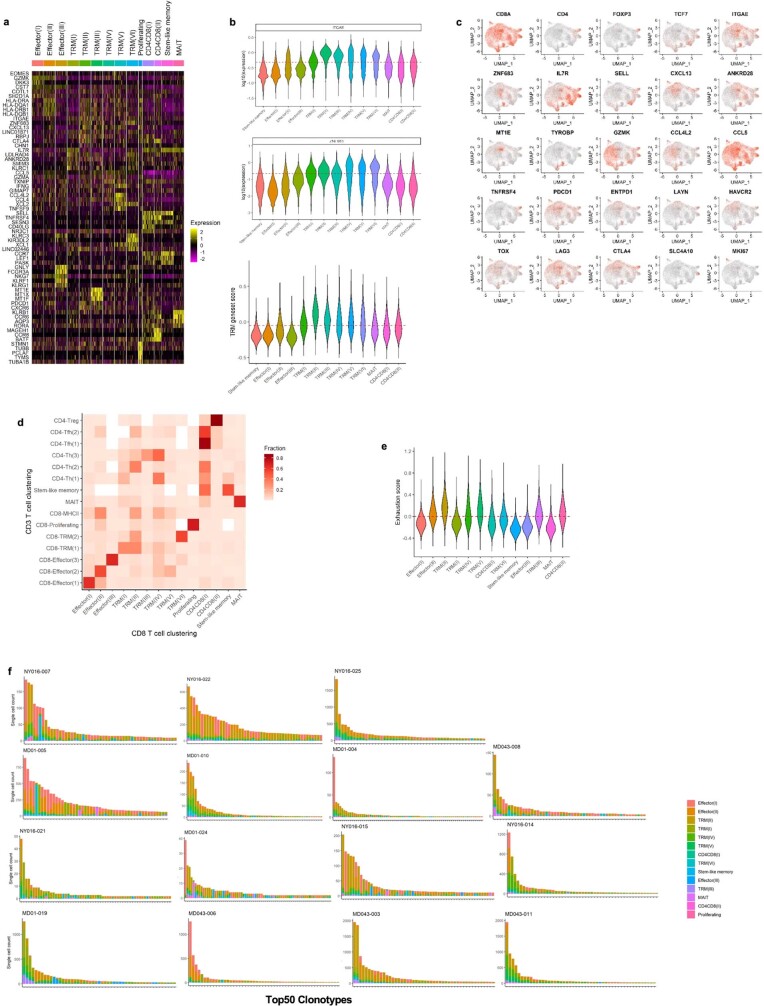
Refined clustering on CD8 T cells. **a**, A heat map shows the top differential genes, ranked by average fold change, for each refined CD8 T cell cluster. 5,000 cells (or all cells in the cluster if cluster size <5000 cells) were randomly sampled from each cluster for visualization (*n* = 16 patients). **b**, Violin plots show the log10 expression of the TRM-defining genes, ITGAE (top) and ZNF683 (HOBIT, middle), and a TRM gene-set score (bottom) for each CD8 T cell cluster. The dashed line indicates the mean expression of the respective gene or gene-set score among all CD8 T cells. Because the proliferating cluster is driven by proliferation-associated genes and represents mixed cell types, this cluster was not shown in the plot. **c**, 2D UMAP red-scale projection of canonical T cell subset marker genes, cell subset selective genes, and immune checkpoints on CD8 T cell subsets. **d**, A heat map shows the proportion of each refined CD8 T cell cluster (Fig. 2b) that is found within each global UMAP T cell cluster (Fig. 1b). This enables visualization of the “parent” cluster for the refined CD8 T cell clusters. **e**, A violin plot shows the exhaustion gene-set score, comprised of a published exhaustion gene list (Supplementary Data 2.2), for each refined CD8 T cell cluster. The dashed line shows the mean exhaustion gene-set score among all CD8 T cells. Because the proliferating cluster is driven by proliferation-associated genes and represents mixed cell types, this cluster was not shown in the plot. **f**, CD8^+^ T cell clonotypic cluster composition. The top 50 CD8^+^ TCR clonotypes in the tumour are shown for each patient, and the proportion of each clonotype that was found within each cluster is designated by the colour code.

We next evaluated the transcriptional programming of MANA- and viral-specific CD8^+^ T cells. Refined clustering of all CD8^+^ T cells (*n* = 235,851) identified 15 unique clusters (Fig. 2b, Extended Data Fig. 5a, Supplementary Data 1.2). Clusters were named on the basis of previously defined T cell states from single-cell transcriptomic studies^16^. Six clusters had gene expression programs consistent with TRM T cells, characterized by high expression of *HOBIT*, *LINC02446*, *CD103* and a previously published TRM gene set (Extended Data Fig. 5b). Selective genes and linkage to the global CD3 T cell clusters shown in Fig. 1 were visualized (Extended Data Figs. 5c, d). The six TRM subsets were heterogenous in their expression of an exhaustion gene set described previously in NSCLC^17^ (Extended Data Fig. 5e, Supplementary Data 2.2). None of the most frequent tumour-infiltrating clonotypes were restricted to a single cluster (Extended Data Fig. 5f). Among all tested individuals, a total of 28 MANA-specific CD8 clonotypes (1,350 total cells from 3 patients with MPR and 3 patients without MPR) as identified by MANAFEST were detected in the single-cell data, of which 20 clonotypes (890 cells) were in the tumour (Fig. 2c, Supplementary Table 8). Of the viral-specific T cell clonotypes, 23 influenza-specific (866 cells) and 2 EBV-specific (281 cells) clones were found in the CD8 single-cell analysis.

**Extended Data Fig. 6:**
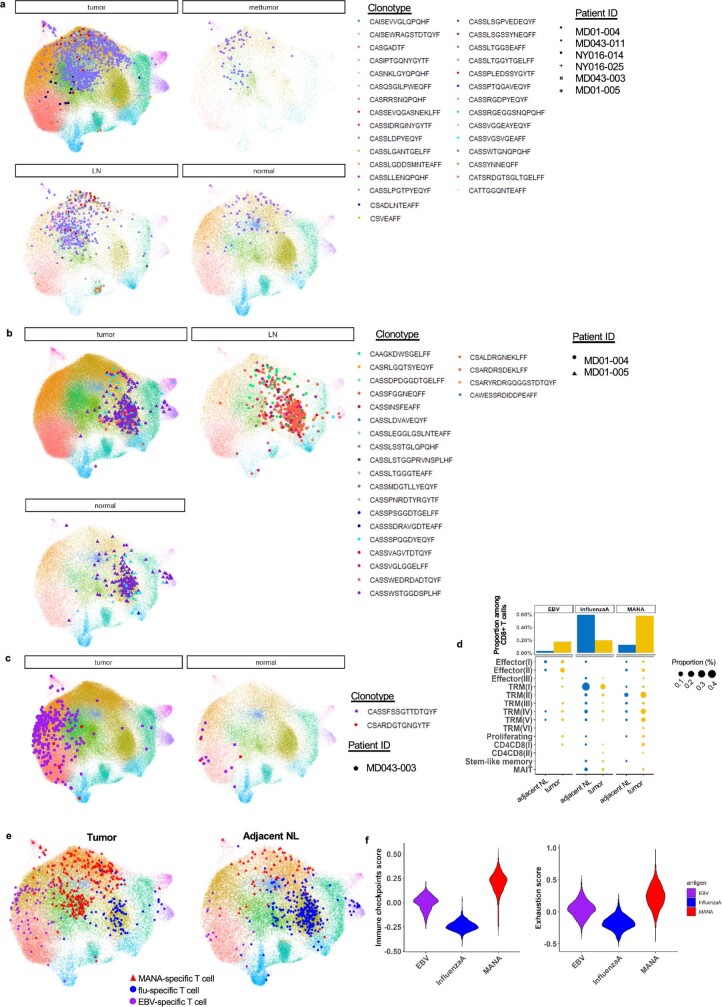
Distinct phenotype of antigen-specific T cells. **a**, Distribution of MANA-specific T cells on UMAP. Individual MANA-specific clonotypes are shown on the UMAP, stratified by tissue compartment and patient ID. Each colour represents a unique MANA-specific clonotype, and each symbol represents a patient. **b**, Distribution of EBV-specific T cells on UMAP. Individual EBV-specific clonotypes are shown on the UMAP, stratified by tissue compartment. Each colour represents a unique EBV-specific clonotype and each symbol represents a patient. **c**, Distribution of influenza-specific T cells on UMAP. Individual influenza-specific clonotypes are shown on the UMAP, stratified by tissue compartment and patient ID. Each colour represents a unique influenza-specific clonotype, and each symbol represents a patient. The CD8 T cell clusters are annotated according to the designation in Fig. 2b. **d**, The barplot (upper) shows the proportion of antigen-specific T cells among total CD8 T cells by tissue compartment (blue bar, adjacent NL; yellow bar, tumour). The dotplot (bottom) shows the proportion of antigen-specific T cells stratified by subset, with the size of the dot representing the proportion among total CD8 T cells (blue dot, adjacent NL; yellow dot, tumour). **e**, TIL and adjacent NL CD8 T cells were downsampled to equal numbers of cells on UMAP before visualization of antigen-specific clonotypes in tumour (left) and adjacent normal lung (right). **f**, The immune checkpoint score and exhaustion score of antigen-specific T cells. A violin plot shows a composite immune checkpoint score (left) and exhaustion score (right) for EBV(purple)-, influenza (blue)-, and MANA (red)-specific T cells.

Overlay of these clonotypes onto the CD8^+^ T cell UMAP demonstrated a marked distinction between the clonotypes with different antigen specificities (Fig. 2c, Extended Data Fig. 6a–c). EBV-reactive T cells primarily resided in effector T (T_eff_) cell clusters, whereas influenza- and MANA-specific T cells largely occupied distinct TRM clusters. Notably, because influenza is a respiratory virus, influenza-specific T cells may be considered the archetypal lung-resident memory T cells^18^. None of the patients in our study were symptomatic for influenza in the six weeks preceding surgery. It is therefore not surprising that influenza-specific CD8 cells were TRM rather than T_eff_ cells. By contrast, EBV-specific T cells exclusively occupied T_eff_ clusters, consistent with periodic acute stimulation upon latent EBV reactivation. Whereas influenza-specific cells were the most abundant in normal lung, MANA-specific CD8 cells were more numerous in the tumour (Extended Data Fig. 6d, e).

There was considerable shared expression of selective cytotoxic T lymphocyte (CTL) activation genes between MANA- and EBV-specific T cells, in particular genes encoding T cell activation and CTL activity, such as *HLA-DRA*, *GZMH*, *IFNG* and *NKG7* (Fig. 2d, Supplementary Data 1.3). However, genes encoding certain canonical cytolytic molecules, such as *GZMK*, were expressed at low levels in MANA-specific TIL. Most notably, *EOMES*, which encodes a transcription factor that is critical for CTL activity^19^, was expressed in EBV-specific CD8 cells but was minimally expressed in most MANA-specific cells. Multiple checkpoints were significantly upregulated in MANA-specific TIL compared with EBV-specific TIL. Notably, MANA-specific cells expressed higher levels of *PRDM1*, which encodes BLIMP-1 and has been reported to participate in coordinated transcriptional activation of multiple checkpoint genes, including *PD-1* (also known as *PDCD1*), *LAG3*, *TIGIT* and *HAVCR2*^20^. *TOX*, which encodes a chromatin modifier important for exhaustion programs of chronic virus-specific and tumour-specific T cells in mouse models^21,22^, was only marginally increased in MANA-specific cells, whereas its homologue, *TOX2*, which has also been reported to drive T cell exhaustion^23^, showed much higher upregulation in MANA-specific versus EBV-specific CD8 TIL. *HOBIT*, which is selectively upregulated in TRM T cells^24^, was also upregulated in MANA-specific TIL, even relative to influenza-specific TRM (Fig. 2e). Indeed, MANA-specific T cells demonstrated the highest immune checkpoint and exhaustion signatures^17^ (Extended Data Fig. 6f). These findings demonstrate that MANA-specific CD8 T cells in the tumour have an unconventional hybrid transcriptional program characterized by incomplete activation of effector programs and significant upregulation of checkpoint molecules such as PD-1, CTLA-4, TIM3, TIGIT and CD39. Genes encoding each of these checkpoint molecules were more highly expressed among MANA-specific CD8 cells than either influenza- or EBV-specific CD8 cells, with *CD39* being the most highly differentially expressed (Fig. 2d, e), congruent with previous flow cytometry findings on MANA-specific lung cancer TIL^2^.

**Extended Data Fig. 7:**
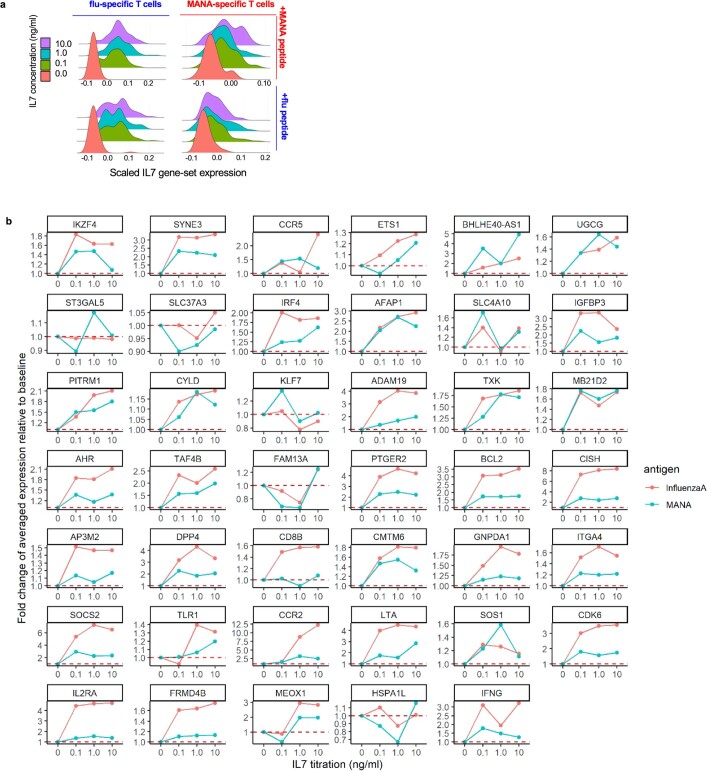
IL-7-induced gene signature between MANA-specific and influenza-specific TIL. TIL from patient MD01-004 were cultured with MD01-004-MANA-12 or influenza A peptide and titrating concentrations of recombinant human IL-7, followed by coupled scRNA-seq–TCR-seq. A total of 814 influenza-specific (410 co-cultured with influenza peptide, 404 co-cultured with MANA peptide) and 581 MANA-specific TIL (366 co-cultured with influenza peptide, 215 co-cultured with MANA peptide) were detected in the single-cell data from a single experiment and were analysed. **a**, Composite expression of an IL-7 gene set by influenza-specific and MANA-specific TIL (as determined by their TCR Vβ CDR3) stimulated with cognate or non-cognate antigen is shown. **b**, Dose–response curve showing the fold change of averaged expression of IL-7-induced genes (Supplementary Data 2.3) that significantly changed from baseline (no IL-7 vs 0.1 ng/ml) in influenza-specific (red) or MANA-specific (blue) T cells. Comparisons were performed using two-sided Wilcoxon rank sum test and adjusted for multiple comparisons using BH method.

**Extended Data Fig. 8:**
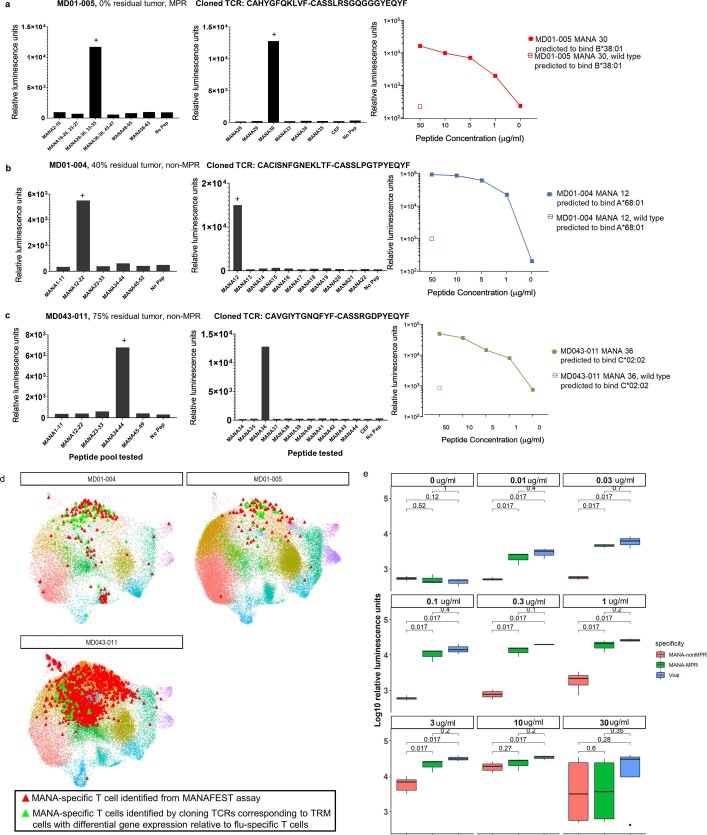
Cloning and dose response of antigen-specific T cells. **a**–**c**, Cloning and screening of TCRs corresponding to CD8 T cells with highly differential gene expression relative to influenza-specific T cells. Seven TCRs were selected from the refined CD8 sc data based on highly differential gene expression relative to influenza-specific T cells. These TCRs were cloned into the Jurkat/NFAT luciferase reporter system and first screened against autologous LCL pre-loaded with pools of putative MANA peptides (10μg/ml) based on the respective patient’s WES and MANA predictions. Three TCRs recognized a MANA peptide pool, one each from patients MD01-005 (**a**), MD01-004 (**b**), and MD043-011 (**c**). The reactive MANA was then mapped from the reactive peptide pool by stimulating the TCR-transfected Jurkat cell with autologous LCL pre-loaded with 10μg/ml of each individual MANA within the reactive pool (centre). Dose–response curves were then generated for each MANA-specific TCR (right). Data are shown as relative luminescence units. A (+) sign indicates the positive response. **d**, Functional characterization of MANAFEST-identified and screening-identified TCRs. 2D projection of clones identified from the MANAFEST assay (red) and clones identified via cloning of TCRs corresponding to T cells with differential gene expression relative to influenza-specific T cells (green) is shown for patients MD01-004, MD01-005, and MD043-011. CD8 T cell clusters are marked with the same colour code as Fig. 2b. **e**, Viral-specific TCRs and MANA-specific TCRs from one patient with MPR and two patients without MPR were cloned into the Jurkat reporter system and tested against titrating concentrations of relevant peptide. The average log_10_ relative luminescence of viral-specific TCRs (blue, 3 clonotypes from 3 different patients), MANA-specific MPR TCRs (green, 3 clonotypes from 1 patient with MPR), and MANA-specific non-MPR TCRs (red, 7 clonotypes from 2 patients without MPR) was compared at each peptide titration. Data are shown as a Box and Whiskers plot. The middle bar shows the median, with the lower and upper hinges corresponding to the 25^th^ and 75^th^ percentiles, respectively (interquartile range, IQR). The upper whisker extends from the hinge to the largest value no further than 1.5 * IQR from the hinge. The lower whisker extends from the hinge to the smallest value at most 1.5 * IQR of the hinge. Comparisons of relative luminescence units for viral-specific vs MANA-specific T cell clonotypes at different titrations were performed using two-sided Wilcoxon rank sum test. ns: *P* > 0.05; *, 0.01 < *P* < 0.05.

Influenza-specific TRM were distinguished from MANA-specific TRM by low levels of both activation and effector CTL programs and had lower expression of multiple checkpoint molecules, but had the highest levels of genes associated with T memory stem cells, such as TCF7 and IL-7R (Fig. 2e, f). Indeed, IL-7R expression was 4.6-fold higher on influenza-specific TIL relative to MANA-specific TIL. In TIL obtained from patient without MPR MD01-004, culture with titrating concentrations of IL-7 in vitro induced much higher levels of IL-7R-regulated genes (Supplementary Data 2.3) in influenza-specific TIL than in MANA-specific TIL (Fig. 2g, Extended Data Fig. 7). Nonetheless, supraphysiological levels of IL-7 induced appreciable upregulation of IL-7R-induced genes in MANA-specific TIL. Given the distinct transcriptional programs of the identified MANA-specific CD8 cells, we hypothesized that other CD8 T cells in the same TRM cluster showing differential expression relative to influenza-specific T cells (Fig. 2g) may also recognize MANA that were not detected by the MANAFEST assay. We cloned seven TCRs corresponding to CD8^+^ T cells with highly differential gene expression relative to influenza-specific T cells. We screened each TCR with a library of candidate MANA (Supplementary Table 6) and confirmed MANA recognition in three of these TCRs, one TCR each from patients MD01-004, MD01-005 and MD043-011 (Extended Data Fig. 8a–d).

To next investigate the ligand-dependent TCR signalling capacity of antigen-specific T cells, we performed a dose–response curve with cognate peptides matched to the ten total Jurkat-validated MANA-specific TCRα–TCRβ pairs (Supplementary Table 10). Peptide dose–response curves of MPR-derived TCRs were comparable to those of EBV- and influenza-specific TCRs, suggesting that these TCRs were capable of strong ligand-dependent signalling (sometimes referred to as functional avidity). However, the peptide dose–response curves of TCRs derived from patients without MPR were markedly lower (approximately 2 log_10_ leftward shift in peptide dose–response curve) (Fig. 2h, Extended Data Fig. 8e). Together, our data show that despite similar measured MANA–HLA binding affinities (Extended Data Fig. 4c, d), TCR from expandable MANA-specific clones from the patient with MPR had significantly higher functional avidity than MANA-specific clones from patients without MPR.

## MANA-specific TIL programs correlate with MPR

**Fig. 3:**
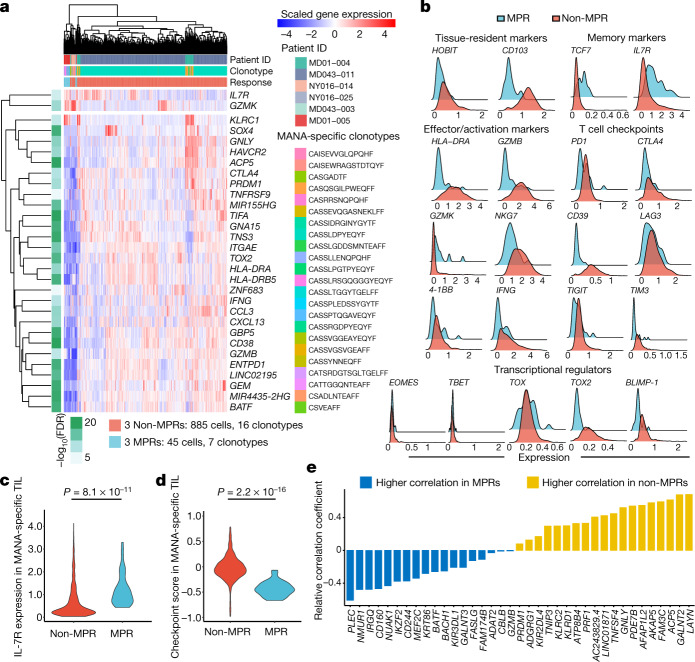
Differential gene expression programs of MANA-specific CD8 T cells in MPR versus non-MPR tumours. Seven unique MANA-specific clonotypes, representing 45 total transcriptomes, were identified in MPR TIL: 39 from MD01-005, 2 from MD043-003 and 4 from NY016-025. In non-MPR TIL, 16 unique clonotypes, representing 885 total transcriptomes, were identified: 782 from MD043-011, 62 from MD01-004 and 22 from NY016-014 (Supplementary Table 8). Differential gene expression analysis was performed on the MANA-specific T cells detected in MPR (*n* = 3) and non-MPR (*n* = 3) tumours. **a**, The top differential genes and selective immune markers of tumour-infiltrating MANA-specific T cells from MPR and non-MPR tumours. Comparisons were performed at the individual cell level using two-sided Wilcoxon rank sum test. *P*-value adjustment was performed using Bonferroni correction. Side bar shows the adjusted *P* value (green scale) and response status (red, TIL from MPR; light blue, TIL from non-MPR). **b**, Histograms show the expression of key genes among MANA-specific T cells from MPR (light blue) and non-MPR (red) tumours. **c**, A violin plot shows IL-7R expression by each MANA-specific CD8 T cell in MPR (red) and non-MPR (light blue) tumours. Comparisons were performed at the individual cell level using two-sided Wilcoxon rank-sum test. **d**, A T cell immune checkpoint score was calculated for each MANA-specific CD8 T cell detected in MPR (red) and non-MPR (light blue) tumours. This checkpoint score was compared between MPR and non-MPR using two-sided Wilcoxon rank-sum test. **e**, The relative correlation coefficient (MPR MANA-specific TIL versus non-MPR MANA-specific TIL) with the immune checkpoint score is shown for genes more highly correlated in non-MPR (yellow) and MPR (blue) TIL.

**Extended Data Fig. 9:**
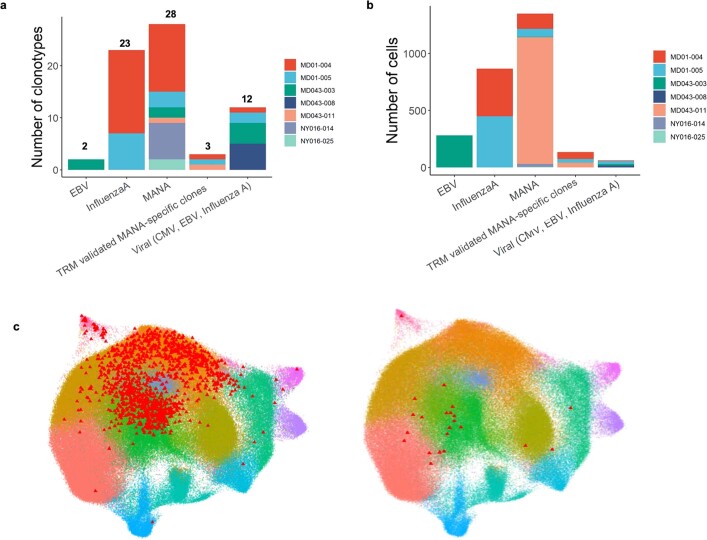
Patient representation of antigen-specific clonotypes. **a**, **b**, Barplots summarize the total number of unique tumour-infiltrating clonotypes (**a**) and cells (**b**), stratified by antigen specificity and method of detection (MANAFEST or based on the TRM gene signature and cloning/peptide screen). Different colours represent the patient identity. **c**, Visualization of clonotypes included in the MANA-specific analysis. The individual UMAP projections of clonotypes that were validated (left) and were not validated (right) by TCR cloning are shown. Of the cells that corresponded to a MANAFEST-identified, MANA-specific clonotype that was detected in the single-cell data, >94% were validated by the jurkat/luciferase TCR cloning system.

**Extended Data Fig. 10:**
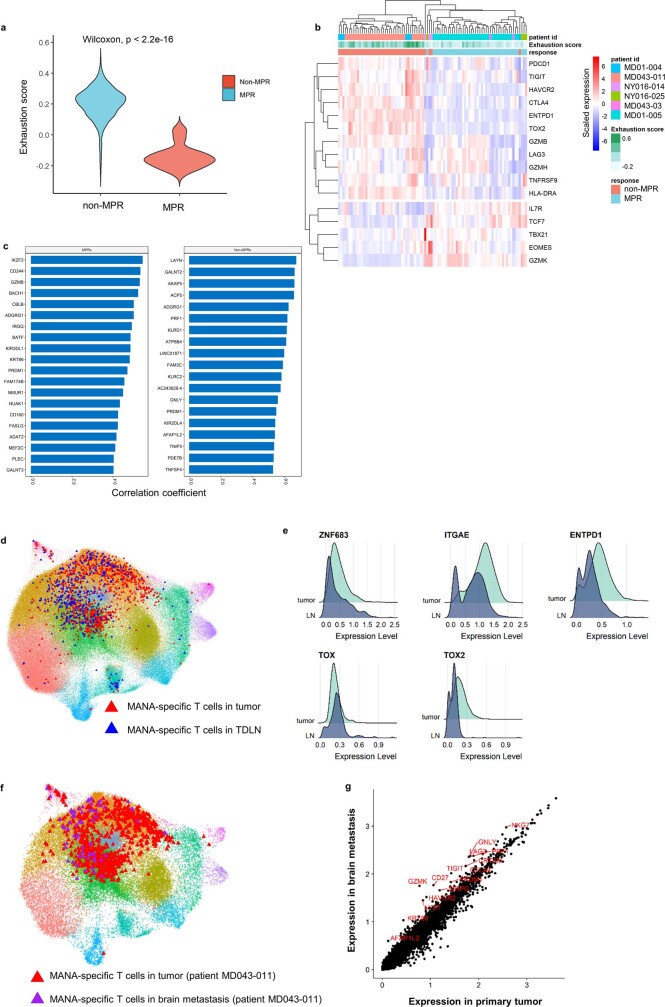
Signatures of MANA-specific T cells according to response and tissue compartment. **a**, Exhaustion score and co-expression of immune checkpoints/effector/memory function gene on MANA-specific TIL. Violin plot shows the exhaustion gene-set score (Supplementary Data 2.2) of MANA-specific TIL of non-MPR (red, *n* = 3) and MPR (light blue, *n* = 3) tumours. Comparisons were performed at the individual cell level using two-sided Wilcoxon rank sum test without multiple comparison adjustment. **b**, Heat map shows co-expression of immune checkpoints and effector/memory genes on MANA-specific TIL. Each column represent a cell. The exhaustion score, response status, and patient IDs are designated by the relevant colour bar. For visualization, MANA-specific T cells were downsampled to the same number of cells from MPR (*n* = 3) and non-MPR (*n* = 3). **c**, Top ranked genes correlated with the immune checkpoint score in MANA-specific TIL. Barplots show the correlation coefficients of the top ranked genes highly correlated with the immune checkpoint score in MPR (left) and non-MPR (right) MANA-specific TIL. **d**, MANA-specific T cells found in the tumour (red triangles) and TDLN (blue triangles) of patients MD01-004, MD01-005, and MD043-011 were projected on the refined CD8 UMAP. **e**, Expression of selective genes is shown for MANA-specific T cells in the tumour and TDLN (*n* = 3). **f**, MANA-specific T cells found in the tumour (red triangle) and brain metastasis (purple triangle) are shown on the UMAP for patient MD043-011. **g**, The scatterplot shows the average expression of genes comparing all refined CD8 T cells from the primary tumour and metastatic brain resection in patient MD043-011. The top differential genes enriched in the brain metastasis are labelled in red. Comparisons were performed at the individual cell level using two-sided Wilcoxon rank sum test. *P*-value adjustment was performed using bonferroni correction. A complete list of differential genes comparing primary tumour at resection vs. the distant brain metastasis is shown in Supplementary Data 1.5. CD8 T cell clusters are marked by the same colour code as Fig. 2b.

To explore determinants of ICB sensitivity, we examined differences in gene expression patterns between MPR and non-MPR MANA-specific TIL. The neoadjuvant clinical trial format enabled us to make this distinction through pathological analysis of surgically resected tissue. In total, we compared 45 MPR TIL transcriptomes (39 from MD01-005, 2 from MD043-003 and 4 from NY016-025) with 885 non-MPR TIL transcriptomes (782 from MD043-011, 62 from MD01-004 and 22 from NY016-014; Extended Data Fig. 9, Supplementary Table 8). We observed highly significant differences between pathologic MPR and non-MPR tumours (Fig. 3a, Supplementary Data 1.4). Significantly higher levels of genes associated with T cell dysfunction such as *TOX2*, *CTLA4*, *HAVCR2* and *ENTPD1* were observed for non-MPR MANA-specific T cells, whereas MPR MANA-specific T cells had higher expression of genes associated with memory (*IL7R* and *TCF7*) and effector function (*GZMK*) (Fig. 3a–c). Both the checkpoint score and exhaustion score were higher in MANA-specific TIL from patients without MPR (Fig. 3d, Extended Data Fig. 10a, b). Of note, *CXCL13* is one of the genes most highly correlated with checkpoint-associated genes in non-MPR MANA-specific TIL, and was also found to be highly expressed in MANA-specific cells relative to virus-specific cells among CD8 TIL (Fig. 2d–f).

A number of genes encoding T cell inhibitory molecules were more highly correlated with a composite immune checkpoint score of MANA-specific TIL from patients without MPR than those from patients with MPR (Fig. 3e, Extended Data Fig. 10c). In two patients without MPR (MD01-004 and MD043-011) and one patient with MPR (MD01-005), we also detected MANA-specific cells upon single-cell profiling of CD8 T cells from TDLN (Extended Data Fig. 10d, e). Tracking the MANA-specific CD8 clonotypes from the primary tumour, we detected those clones among TIL from a brain metastasis resected from patient MD043-011 24 months after primary tumour resection **(**Extended Data Fig. 10f). Relative to the primary tumour, even-higher levels of three checkpoints—LAG3, TIGIT and HAVCR2—were expressed on MANA-specific TIL in the metastasis (Extended Data Fig. 10g, Supplementary Data 1.5).

**Extended Data Fig. 11:**
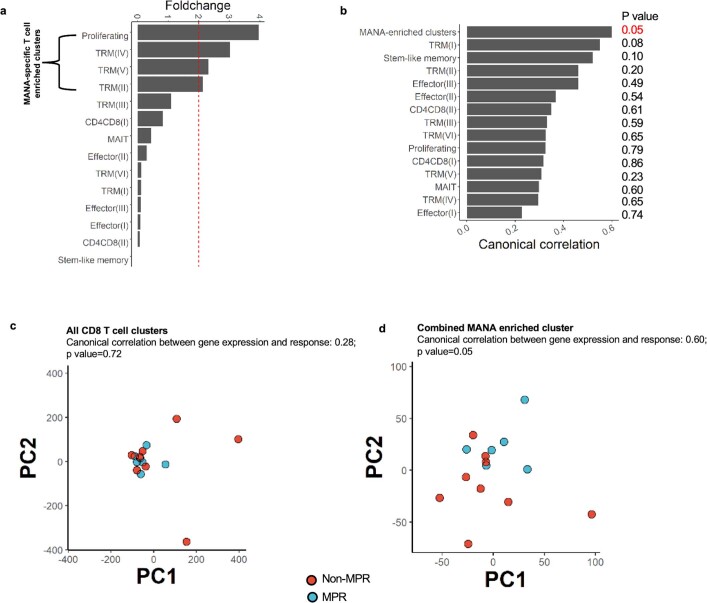
Canonical correlations of CD8 T cell clusters with pathologic response. The canonical correlation between pathologic response status and CD8 T cell clusters vs. a MANA-specific T cell-enriched cluster was evaluated. **a**, Selection of MANA-specific T cell enriched clusters (Proliferating, TRM(IV), TRM (V) and TRM (II)) based on >2 fold change (red dotted line) of MANA-specific T cell frequency relative to random expectation. The above 4 clusters were combined as a ‘MANA-combined’ cluster. **b**, Combined MANA-specific T cell enriched clusters showed the highest canonical correlation with pathologic response. **c**, PCA of pseudobulk gene expression from all CD8 T cell clusters for individual tumour samples (*n* = 15, 6 MPRs and 9 non-MPRs), coloured by response status (MPR as blue blue dots, non-MPR as red dots). **d**, PCA of pseudobulk gene expression from combined MANA enriched T cell cluster for individual tumour samples (*n* = 15, 6 MPRs and 9 non-MPRs), coloured by response status (MPR as light blue dots, non-MPR as red dots). *P* values were obtained using a one-sided permutation test, without correction for multiple comparisons.

Going back to overall TIL transcriptomic patterns, we hypothesized that MANA-specific T cells and/or a MANA-specific T cell-like signature might correlate with response to ICB, even though total TIL single-cell transcriptomic patterns did not (Fig. 1e). Among CD8 TIL from six MPR tumours and nine non-MPR tumours, the greatest correlation with pathologic response status was observed by combining four TIL clusters most highly enriched in MANA-specific cells, whereas the expression profile of total CD8 TIL did not distinguish MPR from non-MPR (Extended Data Fig. 11). These data suggest that additional T cells with this profile may contribute to the anti-tumour response.

## Systemic reprogramming of MANA-specific T cells

**Fig. 4:**
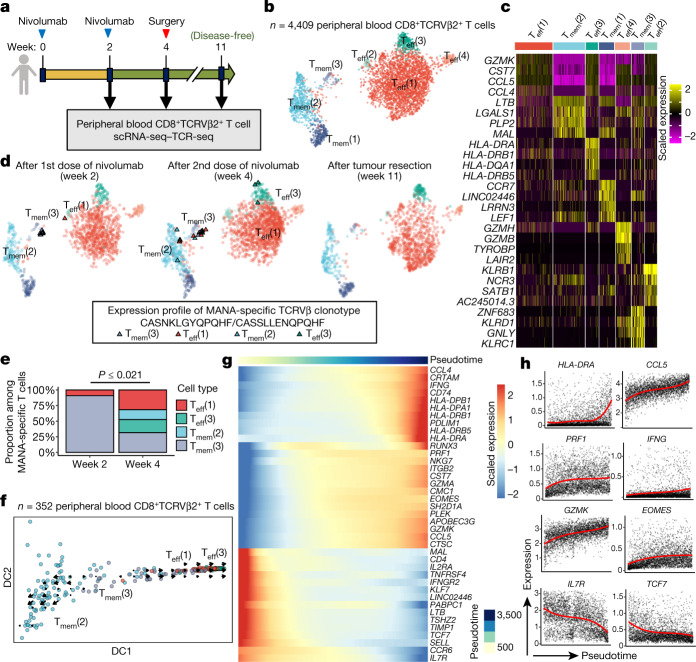
Neoadjuvant PD-1 blockade promotes systemic transcriptional reprogramming in MANA-specific T cells from a patient with complete pathologic response. **a**, Longitudinal peripheral blood mononuclear cells were collected from complete pathologic responder MD01-005 (0% residual tumour) during treatment and in post-surgery follow up. Peripheral blood CD8^+^ T cells were sorted using FACS on the basis of expression of TCRVβ2, which corresponds to the MANA-specific CDR3 CASNKLGYQPQHF, as identified by the MANAFEST assay (Extended Data Fig. 2a). scRNA-seq–TCR-seq was performed on the sorted population from each time point. **b**, UMAP projection of expression profiles of 4,409 peripheral blood CD8^+^TCRVβ2^+^ T cells. **c**, Heat map of the top-5 differential genes, ranked by average fold change, for each T cell cluster. **d**, UMAP projection of MANA-specific T cells, identified via the CASNKLGYQPQHF or CASSLLENQPQHF TCRVβ CDR3, is shown for each time point. Clusters were coloured using the same colour scheme as in **b**. MANA-specific T cells are highlighted as triangles. **e**, The proportions of cells in each T cell cluster among all MANA-specific cells identified at week 2 and week 4 were compared (two-sided Fisher’s exact test and a two-sided test accounting for background cell proportion, both smaller than 0.021; Methods). **f**, Diffusion plot with RNA velocity for clusters in which MANA-specific T cells were detected. Cells were randomly downsampled to 100 cells (or all cells in the cluster if cluster size was smaller than 100 cells) for each cluster for visualization. **g**, Heat map of the top differential genes along the pseudotime trajectory from T_mem_(3) to T_eff_(3). **h**, Pseudotemporal expression of genes that significantly change along the pseudotime from T_mem_(3) to T_eff_(3). Red curves represent the mean temporal function estimates of the three samples from this individual (Methods). Cells with gene expression levels above the top one percentile were removed as outliers.

**Extended Data Fig. 12:**
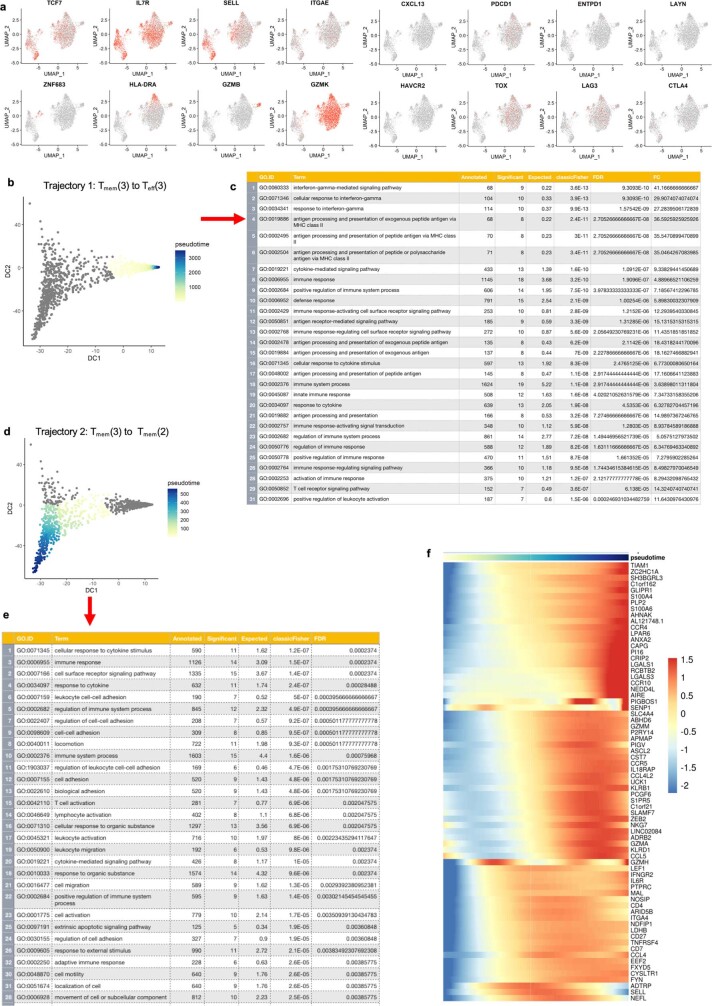
Phenotypic characteristics of FACS-sorted peripheral blood CD8+/Vβ2+ T cells from MPR MD01-005. **a**, Selective gene expression of 2D UMAP red-scale projection is shown of canonical T cell subset marker genes, cell subset selective genes, and immune checkpoints on CD8 T cell subsets sorted from longitudinal peripheral blood of one patient (MD01-005) with complete pathologic response. **b-d**, Pseudotime reconstruction and pseudo-temporal dynamic gene identification in peripheral blood CD8 T cells from a complete pathologic responder. Longitudinal PBMC were collected from complete pathologic responder MD01-005 (0% residual tumour) during treatment and in post-surgery follow up. Peripheral blood CD8^+^ T cells were FACS sorted based on expression of TCR Vβ2, which corresponds to the MANA-specific CDR3 CASNKLGYQPQHF as identified previously via the MANAFEST assay (Extended Data Fig. 2a). scRNA-seq–TCR-seq was performed on the sorted population from each time point. **b**, Constructing the pseudotime axis on the diffusion map from T_mem_(3) to T_eff_(3) as trajectory 1. **c**, GO analysis for genes that significantly change along trajectory 1, ranked by FDR. **d**, Constructing the pseudotime axis on the diffusion map from T_mem_(3) to T_mem_(2) as trajectory 2. **e**, GO analysis for genes that significantly change along trajectory 2, ranked by FDR. **f**, Heat map showing genes that significantly change along trajectory 2 (FDR < 0.05).

We next performed scRNA-seq–TCR-seq of serial peripheral blood T cells from patient with MPR MD01-005 after enriching for expression of the TCR-Vβ genes corresponding to this patient’s MANA-specific TCRs using fluorescence-activated cell sorting (FACS) (Fig. 4a–c, Extended Data Fig. 12a). Nine out of ten MANA-specific clones mapped to a TRM-like cluster (T_mem_(3); T_mem_, memory T cell), with some transcriptional features of TRM, such as expression of *HOBIT*) two weeks after the initiation of anti-PD-1 treatment (Fig. 4d). By four weeks (time of tumour resection), a significant diversification of phenotype was observed (*P* ≤ 0.021; Methods). Half of the MANA-specific cells were in T_eff_ clusters (Fig. 4e). By 11 weeks (7 weeks after tumour resection), the MANA-specific cells were below the limit of detection in the blood, consistent with known TRM patterns in the peripheral blood^25^. Using RNA velocity, we observed a clear bidirectional flow of TRM-like memory MANA-specific T cells in the T_mem_(3) cluster towards either an activated effector (T_eff_(3)) or a T_mem_(2) transcriptional profile (Fig. 4f). Genes associated with T_eff_ cell function and activation, T cell homing and migration, and tissue retention were upregulated along the pseudotime from T_mem_(3) to T_eff_(3), whereas there was a decrease in genes associated with resting memory T cells (Fig. 4g, h). Gene Ontology (GO) analysis revealed significant enrichment of an IFNγ-mediated signalling pathway along the differentiation trajectory (Extended Data Fig. 12b–f). Although all these tissue compartments were only available for one MPR, these findings are consistent with our hypothesis that, upon activation, functional effector MANA-specific T cells enter the blood and traffic into tissues, including normal lung, in search of micro-metastatic tumour^26^, and are compatible with a previous study showing that TRM cell plasticity can influence systemic memory T cell responses^27^.

## Discussion

Here we describe the transcriptional programming of MANA-specific TIL after ICB in lung cancer, and further, differential gene programs between patients whose tumours show MPR versus those that do not. Using the MANAFEST platform, MANA-specific CD8 T cells in peripheral blood were detected in the majority of patients who were treated with anti-PD-1; these were also found among TIL in roughly a third of these individuals. Detection of these T cells was independent of tumour response, suggesting that factors in the tumour microenvironment affecting T cell function probably contribute to anti-tumour responsiveness. Indeed, the most frequent MANA-specific clonotype, representing 782 TIL, was observed in a patient with no MPR. This tumour had dual *KRAS* and *STK11* oncogenic mutations, which are known to be highly associated with non-response to PD-1 blockade^28^. Consistent with an earlier study^2^, *CD39* expression was a key difference between MANA-specific and viral-specific T cells. Among MANA-specific CD8 TIL, roughly 90% were TRM with high expression of *HOBIT* that also displayed a partial but incompletely activated T_eff_ program, along with upregulation of several targetable checkpoints in non-MPR tumours. MANA-specific T cells also express far less IL-7R relative to influenza TRM, translating functionally into poor IL-7 responsiveness. These features may all contribute to their limited tumour-specific responsiveness in contrast to anti-viral responses. Future studies are warranted to assess the diminished functional capacity of MANA-specific T cells that was suggested by the transcriptomic profiles observed in our study.

One hypothesis for the lack of ICB response in some patients is that tumour-specific T cells exhibit low activity owing to poor avidity or affinity of their TCR for its cognate peptide MHC. Our finding comparing the ligand-induced TCR signalling of three MANA-specific TCRs from MPR TIL with seven from patients without MPR supports this notion, although additional studies of this type are necessary to definitively test the hypothesis. An overall limitation of these studies is the modest number of MANA-specific cells among TIL that we were able to detect, representing three responders and three non-responders. Indeed, identification of MANA-specific cells is experimentally challenging, and only a few studies have successfully identified these cells in NSCLC^2,3,8,29,30^, yet none of these profiled the transcriptome of MANA-specific T cells at single-cell resolution. Among the 930 MANA-specific transcriptomes that we identified in TIL, there was high consistency among cells from each response group in highly differential expression of key genes known to regulate T cell function. These findings inform on potential ICB combination therapies to overcome anti-PD-1 resistance that occurs even in the presence of potent MANA-specific T cells. For example, our data demonstrated reduced activation of transcriptional programs downstream of IL-7 ligation in MANA-specific TIL relative to influenza-specific TIL, but the MANA-specific TIL retain their ability to respond to supraphysiological levels of IL-7. Because IL-7 signalling is a requisite for maintenance of T cell homeostasis and long-lived memory, it is conceivable that targeting the IL-7 pathway could enhance ICB response. Our findings thus provide a platform for follow-up studies to more rigorously test the generalizability of our conclusions in the setting of resectable and metastatic NSCLC.

## Methods

No statistical methods were used to predetermine sample size. The experiments were not randomized. The investigators were not blinded to allocation during experiments and outcome assessment.

### Patients and biospecimens

This study was approved by the Institutional Review Boards (IRB) at Johns Hopkins University (JHU) and Memorial Sloan Kettering Cancer Center and was conducted in accordance with the Declaration of Helsinki and the International Conference on Harmonization Good Clinical Practice guidelines. The patients described in this study provided written informed consent. All biospecimens were obtained from patients with stage I-IIIA NSCLC who were enrolled to a phase II clinical trial evaluating the safety and feasibility of administering two doses of anti-PD-1 (nivolumab) before surgical resection. Pathological response assessments of primary tumours were reported previously^8,31^. Tumours with no more than 10% residual viable tumour cells were considered to have a MPR.

### scRNA-seq–TCR-seq

Cryobanked T cells were thawed and washed twice with pre-warmed RPMI with 20% FBS and gentamicin. Cells were resuspended in PBS and stained with a viability marker (LIVE/DEAD Fixable Near-IR; ThermoFisher) for 15 min at room temperature in the dark. Cells were the incubated with Fc block for 15 min on ice and stained with antibody against CD3 (BV421, clone SK7) for 30 min on ice. After staining, highly viable CD3^+^ T cells were sorted into 0.04% BSA in PBS using a BD FACSAria II Cell Sorter. Sorted cells were manually counted using a hemocytometer and prepared at the desired cell concentration (1,000 cells per μl), when possible. The Single Cell 5′ V(D)J and 5′ DGE kits (10X Genomics) were used to capture immune repertoire information and gene expression from the same cell in an emulsion-based protocol at the single-cell level. Cells and barcoded gel beads were partitioned into nanolitre-scale droplets using the 10X Genomics Chromium platform to partition up to 10,000 cells per sample followed by RNA capture and cell-barcoded cDNA synthesis using the manufacturer’s standard protocols. Libraries were generated and sequenced on an Illumina NovaSeq instrument using 2 × 150-bp paired end sequencing. 5′ VDJ libraries were sequenced to a depth of ~5,000 reads per cell, for a total of 5 million to 25 million reads. The 5′ DGE libraries were sequenced to a target depth of ~50,000 reads per cell.

### Whole-exome sequencing, mutation calling and neoantigen prediction

Genomic data for most individuals in our study were reported previously^8^, and whole-exome sequencing, variant calling and neoantigen predictions for individuals MD043-003 and NY016-025 were performed prospectively for the present study. Whole-exome sequencing was performed on pre-treatment tumours unless otherwise noted (Supplementary Table 4) and matched normal samples. DNA was extracted from tumours and matched peripheral blood using the Qiagen DNA kit (Qiagen). Fragmented genomic DNA from tumour and normal samples was used for Illumina TruSeq library construction (Illumina) and exonic regions were captured in solution using the Agilent SureSelect v.4 kit (Agilent,) according to the manufacturers’ instructions as previously described^32^. Paired-end sequencing, resulting in 100 bases from each end of the fragments for the exome libraries was performed using Illumina HiSeq 2000/2500 instrumentation (Illumina). The depth of total and distinct coverage is shown in Supplementary Table 4. Somatic mutations, consisting of point mutations, insertions, and deletions across the whole exome were identified using the VariantDx custom software for identifying mutations in matched tumour and normal samples as previously described^32,33^. Somatic mutations, consisting of nonsynonymous single base substitutions, insertions and deletions, were evaluated for putative MHC class I neoantigens using the ImmunoSelect-R pipeline (Personal Genome Diagnostics) as previously described^30^. Somatic sequence alterations are listed in Supplementary Table 5.

### Identification of neoantigen-specific TCRVβ CDR3 clonotypes

We used the MANAFEST assay^3^ to evaluate T cell responsiveness to MANA and viral antigens. In brief, pools of MHC class I-restricted CMV, EBV and influenza peptide epitopes (CEFX, jpt Peptide Technologies), pools representing the matrix protein and nucleoprotein from H1N1 and H3N2 (jpt Peptide Technologies), and putative neoantigenic peptides defined by the ImmunoSelect-R pipeline (jpt Peptide Technologies; Supplementary Table 6) were each used to stimulate 250,000 T cells in vitro for 10 days as previously described^3^. The time point of peripheral blood collection used for each MANAFEST assay is described in Supplementary Tables 2, 7. In brief, on day 0, T cells were isolated from PBMC by negative selection (EasySep; STEMCELL Technologies). The T cell-negative fraction was co-cultured with an equal number of selected T cells in culture medium (IMDM/5% human serum with 50 μg ml^−1^ gentamicin) with 1 μg ml^-1^ relevant neoantigenic peptide, 1 μg ml^−1^ of an MHC class I-restricted CMV, EBV, and influenza peptide epitope pool (CEFX, jpt Peptide Technologies), 1 μg ml^−1^ of pools representing the matrix protein and nucleoprotein from H1N1 and H3N2 (jpt Peptide Technologies), or no peptide (to use as a reference for non-specific or background clonotypic expansion). On day 3, half the medium was replaced with fresh medium containing cytokines for a final concentration of 50 IU ml^−1^ IL-2 (Chiron), 25 ng ml^−1^ IL-7 (Miltenyi) and 25 ng ml^−1^ IL-15 (PeproTech). On day 7, half the medium was replaced with fresh culture medium containing cytokines for a final concentration of 100 IU ml^−1^ IL-2 and 25 ng ml^−1^ IL-7 and IL-15. On day 10, cells were harvested, washed twice with PBS, and the CD8^+^ fraction was isolated using a CD8^+^ negative enrichment kit (EasySep; STEMCELL Technologies). DNA was extracted from each CD8-enriched culture condition using the Qiamp micro-DNA kit according to the manufacturer’s instructions. TCR sequencing was performed on each individual peptide-stimulated T cell culture using survey-level sequencing (max depth ~60,000 reads) by Adaptive Biotechnologies using their established platform^34^ or by the Sidney Kimmel Comprehensive Cancer Center FEST and TCR Immunogenomics Core (FTIC) facility using the Oncomine TCR Beta short-read assay (Illumina) and sequenced on an Illumina iSeq 100 using unique dual indexes, for a maximum of ~40,000 reads per sample.

Data pre-processing was performed to eliminate non-productive TCR sequences and to align and trim the nucleotide sequences to obtain only the CDR3 region. Sequences not beginning with C or ending with F or W and having less than seven amino acids in the CDR3 were eliminated. TCR sequencing samples with less than 1,000 productive reads were excluded from downstream analysis. MD043-011-MANA_22 was the only such sample in the present study (see Supplementary Table 7). Resultant processed data files were uploaded to our publicly available MANAFEST analysis web app (http://www.stat-apps.onc.jhmi.edu/FEST) to bioinformatically identify antigen-specific T cell clonotypes.

Bioinformatic analysis of productive clones was performed to identify antigen-specific T cell clonotypes meeting the following criteria: (1) significant expansion (Fisher’s exact test with Benjamini–Hochberg correction for false discovery rate (FDR), *P* < 0.05) compared to T cells cultured without peptide, (2) significant expansion compared to every other peptide-stimulated culture (FDR <0.05) except for conditions stimulated with similar neoantigens derived from the same mutation, (3) an odds ratio >5 compared to the no peptide control, and (4) present in at least 10% of the cultured wells to ensure adequate distribution among culture wells. A lower read threshold of 300 was used for assays sequenced by the FTIC and a lower threshold of 30 was used for samples sequenced by Adaptive Biotechnologies. In MANAFEST assays testing less than 10 peptides or peptide pools, cultures were performed in triplicate and reactive clonotypes were defined as being significantly expanded relative to T cells cultured without peptide (FDR <0.05) in two out of three triplicates, and not significantly expanded in any other well tested. When available, TCRseq was also performed on DNA extracted from tumour, normal lung, and lymph node tissue obtained before treatment and at the time of surgical resection, as well as serial peripheral blood samples. The assays performed on each biospecimen are outlined in Supplementary Table 2.

### Peptide affinity and stability measurements

Peptide affinity for cognate HLA molecules was assessed using a luminescent oxygen channeling immunoassay (LOCI; AlphaScreen, Perkin Elmer) as previously described^35^. This is a proximity-based system using a donor and acceptor bead, each conjugated with an epitope tag. When the donor bead is excited with light at 650 nm and can activate an acceptor bead, resulting in a signal at 520–620 nm, which can be quantified per second as a surrogate of affinity. A higher number of counts per second indicates higher affinity of the peptide:HLA pair. The stability of peptide loaded complexes was measured by refolding MHC with peptide and subsequently challenging complexes with a titration of urea. The denaturation of MHC was monitored by ELISA as described previously^36^.

### TCR reconstruction and cloning

Ten MANAFEST+ TCR sequences for which the TCRα chain could be enumerated (>3 cells in single-cell data with the same TCRα–TCRβ pair) were selected for cloning. In addition, seven clones (from three individuals: MD01-004, MD01-005 and MD043-011) that have high composite signature (using the AddModuleScore function) consisting of differential gene programs of MANA-specific T cell relative to influenza-specific T cells in the TRM were selected for cloning. Relevant TCRs were analysed with the IMGT/V-Quest database (http://www.imgt.org). The database allows us to identify the TRAV and TRBV families with the highest likelihood to contain the identified segments which match the sequencing data. To generate the TCRs, the identified TCRA V-J region sequences were fused to the human TRA constant chain, and the TCRB V-D-J regions to the human TRB constant chain. The full-length TCRA and TCRB chains were then synthesized as individual gene blocks (IDT) and cloned into the pCI mammalian expression vector, containing a CMV promoter, and transformed into competent *Escherichia coli* cells according to the manufacturer’s instructions (NEBuilder HiFi DNA Assembly, NEB). Post transformation and plasmid miniprep, the plasmids were sent for Sanger sequencing to ensure no mutations were introduced (Genewiz).

### T cell transfection, transient TCR expression and MANA-recognition assays

To generate a Jurkat reporter cell in which we could transfer our TCRs of interest, the endogenous TCR α- and β-chains were knocked out of a specific Jurkat line that contains a luciferase reporter driven by an NFAT response element (Promega) using the Alt-R CRISPR system (Integrated DNA Technologies, IDT). Two sequential rounds of CRISPR knockout were performed using crDNA targeting the TCRα constant region (AGAGTCTCTCAGCTGGTACA) and the TCRβ constant region (AGAAGGTGGCCGAGACCCTC). Limiting dilution was then used to acquire single cell clones and clones with both TCRα and TCRβ knocked out, as confirmed by Sanger sequencing and restoration of CD3 expression only by the co-transfection of TCRα or TCRβ chains, were chose. CD8α and CD8β chains were then transduced into the TCRα^−^TCRβ^−^ Jurkat reporter cells using the MSCV retroviral expression system (Clontech). Jurkat reporter cells were then co-electroporated with the pCI vector encoding the TCRB and TCRA gene blocks, respectively, using ECM830 Square wave electroporation system (BTX) at 275 V for 10 ms in OptiMem media in a 4-mm cuvette. Post electroporation, cells were rested overnight by incubating in in RPMI 10% FBS at 37 °C, 5% CO_2_. TCR expression was confirmed by flow cytometric staining for CD3 on a BD FACSCelesta and 50,000 CD3^+^ T cells were plated in each well of a 96-well plate. Reactivity of the TCR-transduced Jurkat cells was assessed by co-culturing with 1 × 10^5^ autologous EBV-transformed B cells, loaded with titrating concentrations of MANA peptides, viral peptide pools or negative controls. After overnight incubation, activation of the NFAT reporter gene was measured by the Bio-Glo Luciferase Assay per manufacturer’s instructions (Promega). Jurkat cells were routinely tested for mycoplasma contamination. No cell line authentication was performed.

### COS-7 transfection with HLA allele and p53 plasmids

gBlocks (IDT) encoding HLA A*68:01, p53(R248L) and wild-type p53 were cloned into pcDNA3.4 vector (Thermo Fisher Scientific, A14697). COS-7 cells were transfected with plasmids at 70–80% confluency using Lipofectamine 3000 (Thermo Fisher Scientific, L3000015) and incubated at 37 °C overnight in T75 flasks. A total of 30 μg plasmid (1:1 ratio of HLA plasmid per target protein plasmid in co-transfections) was used. Post transfection, COS-7 cells were plated with TCRαβ-transfected JurkaT cells containing NFAT reporter gene at a 1:1 ratio. After overnight incubation, activation of the NFAT reporter gene was measured by the Bio-Glo Luciferase Assay per manufacturer’s instructions (Promega).

### Single-cell data pre-processing and quality control

Cell Ranger v3.1.0 was used to demultiplex the FASTQ reads, align them to the GRCh38 human transcriptome, and extract their cell and unique molecular identifier (UMI) barcodes. The output of this pipeline is a digital gene expression (DGE) matrix for each sample, which records the number of UMIs for each gene that are associated with each cell barcode. The quality of cells was then assessed based on (1) the number of genes detected per cell and (2) the proportion of mitochondrial gene/ribosomal gene counts. Low-quality cells were filtered if the number of detected genes was below 250 or above 3× the median absolute deviation away from the median gene number of all cells. Cells were filtered out if the proportion of mitochondrial gene counts was higher than 10% or the proportion of ribosomal genes was less than 10%. For single-cell VDJ sequencing, only cells with full-length sequences were retained. Dissociation/stress associated genes^37,38^, mitochondrial genes (annotated with the prefix ‘MT-’), high abundance lincRNA genes, genes linked with poorly supported transcriptional models (annotated with the prefix ‘RP-’)^39^ and TCR (TR) genes (TRA/TRB/TRD/TRG, to avoid clonotype bias) were removed from further analysis. In addition, genes that were expressed in less than five cells were excluded.

### Single-cell data integration and clustering

Seurat^40^ (3.1.5) was used to normalize the raw count data, identify highly variable features, scale features, and integrate samples. PCA was performed based on the 3,000 most variable features identified using the vst method implemented in Seurat. Gene features associated with type I Interferon (IFN) response, immunoglobulin genes and specific mitochondrial related genes were excluded from clustering to avoid cell subsets driven by the above genes^39^. Dimension reduction was done using the RunUMAP function. Cell markers were identified by using a two-sided Wilcoxon rank sum test. Genes with adjusted *P* <0.05 were retained. Clusters were labelled based on the expression of the top differential gene in each cluster as well as canonical immune cell markers. Global clustering on all CD3 T cells and refined clustering on CD8 T cells were performed using same procedure. To select for CD8^+^ T cells, SAVER^41^ was used to impute dropouts by borrowing information across similar genes and cells. A density curve was fitted to the log_2_-transformed SAVER imputed CD8A expression values (using the ‘density’ function in R) of all cells from all samples. A cut-off is determined as the trough of the bimodal density curve (that is, the first location where the first derivative is zero and the second derivative is positive). All cells with log_2_-transformed SAVER imputed CD8A expression larger than the cut-off are defined as CD8^+^ T cells. TRB amino acid sequences were used as a biological barcode to match MANA, EBV or influenza A-specific T cell clonotypes identified from the FEST assay with single-cell VDJ profile and were projected onto CD8^+^ T cell refined UMAP.

### Single-cell subset pseudobulk gene expression analysis

PCA was performed on a standardized pseudobulk gene expression profile, where each feature was standardized to have a mean of zero and unit variance. In global CD3 and CD8 TIL PCA, for each cell cluster we first aggregated read counts across cells within the cluster to produce a pseudobulk expression profile for each sample and normalized these pseudobulk expression profiles across samples by library size. Combat function in the sva R package^42,43^ was applied to address potential batch effects on the normalized pseudobulk profile. Highly variable genes (HVGs) were selected for each cell cluster by fitting a locally weighted scatterplot smoothing (LOESS) regression of standard deviation against the mean for each gene and identifying genes with positive residuals. For each sample, all cell clusters were then concatenated by retaining each cluster’s HVGs to construct a concatenated gene expression vector consisting of all highly variable features identified from different cell clusters. Each element in this vector represents the pseudobulk expression of a HVG in a cell cluster. Samples were embedded into the PCA space based on these concatenated gene expression vectors. Canonical correlation^44,45^ between the first two PCs (that is, PC1 and PC2) and a covariate of interest (that is, tissue type or response status) was calculated. Permutation test was used to assess the significance by randomly permuting the sample labels 10,000 times. In the MANA-specific PCA (Extended Data Fig. 11), MANA-enriched cell clusters, defined by clusters with MANA-specific T cell frequency at least two fold higher than randomly expected, were aggregated as one combined cell cluster. Then, a similar procedure by first identifying HVGs, computing the first 2 PCs and then calculating the canonical correlation was repeated for the combined MANA-enriched cell cluster and each of the other CD8 clusters.

### Differential analysis comparing MPR and non-MPR by total CD8 or CD4 TIL and by cell cluster

The gene expression read counts were adjusted by library size. SAVER^41^ was used to impute the dropouts, and further log2-transformed the imputed values after adding a pseudocount of 1. A linear mixed-effect model^46^ was constructed to identify genes that are significantly differential between MPR and non-MPR among total CD8/CD4 TIL and by each cell cluster, respectively. The B-H procedure^47^ was used to adjust the *P* values for multiple testing, and the statistical significance is determined using a cut-off of FDR <0.05.

### Differential-expression tests and antigen-specific T cell marker genes

Differential-expression tests for antigen-specific T cells were performed using FindAllMarkers functions in Seurat with Wilcoxon rank-sum test on SAVER imputed expression values. Genes with >0.25 log_2_-fold changes, at least 25% expressed in tested groups, and Bonferroni-corrected p values <0.05 were regarded as significantly differentially expressed genes (DEGs). Antigen-specific (MANA versus influenza versus EBV) T cell marker genes were identified by applying the differential expression tests for upregulated genes between cells of one antigen specificity to all other antigen-specific T cells in the dataset. MANA-specific T cell genes associated with response to ICB were identified by applying the differential expression tests comparing MANA-specific T cells from MPR versus those from non-MPR. Top ranked DEGs (by log-fold changes) with a log_2_-fold changes >0.8 and DEGs relating to T cell function were extracted for further visualization in a heat map using pheatmap package. SAVER-imputed expression values of selective marker genes (transcriptional regulators, memory markers, tissue-resident markers, T cell checkpoints, effector and activation markers) were plotted using the RidgePlot function in Seurat.

### In vitro short-term TIL stimulation with IL-7

Cryopreserved TIL from patient MD01-004 were thawed, counted, and stained with the LIVE/DEAD Fixable Aqua (ThermoFisher) viability marker and antibodies specific for CD3 (PE, clone SK1) and CD8 (BV786, clone RPA-T8). Thirty-thousand CD8^+^ T cells per condition were sorted on a BD FACSAria II Cell Sorter into a 96-well plate. Autologous T cell-depleted PBMC were added as antigen presenting cells (APC) at 1:1 ratio. The cells were stimulated with either influenza A or MD01-004-MANA 12 peptide and titrating concentrations of recombinant human IL-7 (Miltenyi) for 12 h in a round-bottomed 96-well plate.

### Gene expression analysis of IL-7-stimulated MANA- and influenza-specific TIL

Following 12 h of antigen and IL-7 stimulation, cells were spun down, counted and re-suspended in 1% BSA at desired concentration. scRNA-seq and VDJ libraries were prepared using 10X Chromium single cell platform using 5′ DGE library preparation reagents and kits according to manufacturer’s protocols (10X Genomics) and as described above. MANA- or influenza-specific T cell clonotypes from the single-cell dataset were identified by using the TRB amino acid sequences as a biological barcode. SAVER imputed gene expression was scaled and centred using the ScaleData function in Seurat. A composite score for the IL-7-upregulated gene set^48^ (Supplementary Data 2.3) expression was computed using the AddModuleScore function and subsequently visualized using ridgeplot. Mean ± standard error was used to show the dose–response curve of the IL-7-upregulated gene-set score by antigen-specific T cells and peptide-stimulation groups.

### Immune checkpoint and exhaustion score generation and highly correlated genes

To characterize dysfunctional CD8 MANA TIL, six best-characterized (and clinically targeted) checkpoints: CTLA4, PDCD1, LAG3, HAVCR2, TIGIT and ENTPD1, were used to compute the T cell checkpoint score, and a published gene list from exhausted T cells was used to compute the T cell exhaustion score, using AddModuleScore function in Seurat. Applying T cell checkpoint score as an anchor, genes that were maximally correlated to the score were identified using linear correlation in MANA-specific TIL from MPR and non-MPR, respectively. Top-30 genes (from HVG selected using FindVariableGenes function in Seurat and excluded the 6 genes included in immune checkpoint score generation) with the highest correlation coefficients were plotted as a bar plot. The difference of correlation coefficients of the above genes was additionally computed between MPR and non-MPR and visualized using waterfall plot.

### Evaluation of peripheral MANA-specific T cell transcriptome changes during treatment

Peripheral blood T cells from patient MD01-005 were sorted based on expression of CD8 and TCRVβ2, followed by scRNA-seq–TCR-seq and clustering on conventional CD8^+^ T cells (MAIT cells excluded). To evaluate whether there was a statistically significant change in the cell types of MANA cells between week 2 (W2) and week 4 (W4) samples in Fig. 4d, e, we first conducted a Fisher’s exact test, which yields a *P* = 0.021, indicating a statistically significant phenotype change in MANA-specific cells (Fig. 4e). We also conducted a more sophisticated test that adjust for potential background differences in cell type abundance between W2 and W4 samples. In this test, we let *m*_*c,t*_ denote the probability that a MANA-specific T cell collected at time point *t* (W2 or W4) comes from cell type *c*, and let *p*_*c,t*_ denote the proportion of all cells in time point *t* that come from cell type *c*. We evaluated the ratio *R*_*c,t*_ = *m*_*c,t*_*/p*_*c,t*_, which characterizes the relative abundance of MANA-specific T cells in each cell type. We compared the null model where this ratio does not change over time (H_0_: *R*_*c,W2*_ = *R*_*c,W4*_ for all cell type *c*) versus the alternative model where W2 and W4 T cells have different ratios (H_1_: *R*_*c,W2*_ ≠ *R*_*c,W4*_). To do this, we computed the test statistic $S=\sum _{c}{({R}_{c,W2}-{R}_{c,W4})}^{2}$ using the observed data and compared it to its null distribution obtained using Monte Carlo simulations. To construct the null distribution for $S$, we pooled cells from W2 and W4 together and treated them as one sample to estimate the common ratio *R*_*c,W2*_ = *R*_*c,W4*_ = *R*_*c*_ shared by W2 and W4, and then derived the probability that a MANA-specific T cell collected at time point *t* comes from cell type *c* under the null model H_0_, which is proportional to *p*_*c,t*_ *R*_*c*_ (that is, the product of the sample-specific background cell type proportion *p*_*c,t*_ and the common MANA-abundance ratio *R*_*c*_ shared between samples). The MANA-specific T cells at time point *t* were then redistributed to different cell types randomly based on a multinomial distribution with this expected MANA-specific T cell type proportion (that is, the expected probability that a MANA-specific T cell at time point *t* comes from cell type *c* under H_0_ is ${p}_{c,t}{R}_{c}/(\sum _{c{\prime} }{p}_{{c}^{{\prime} },t}{R}_{{c}^{{\prime} }})$), while keeping the total number of MANA-specific T cells at each time point the same as the observed MANA-specific T cell number at that time point. The test statistic *S* was then computed using this simulated sample. We repeated this simulation 10,000 times to derive the null distribution of *S*. Comparing the observed *S* to its null distribution yields a *P* < 10^−4^.

### RNA velocity-based differentiation-trajectory tracing

The RNA velocity analysis was performed by first recounting the spliced reads and unspliced reads based on aligned bam files of scRNA-seq data using the velocyto Python package. The calculation of RNA velocity values for each gene in each cell and embedding RNA velocity vector to low-dimension space were done using the SeuratWapper workflow for estimating RNA velocity using Seurat (https://github.com/satijalab/seurat-wrappers/blob/master/docs/velocity.md). The first two diffusion components from Diffusion map were used to construct the coordinates along with velocity. TSCAN (v.1.7.0) was used to reconstruct the cellular pseudotime on diffusion maps space for the PBMC T cells from three time points (samples) of one patient (MD01-005). Based on velocity analysis, the T_mem_(3) cluster was specified as the starting cluster for the pseudotemporal trajectory which has branches. For each branch, log_2_-transformed and library size-normalized SAVER-imputed gene expression values were used for analysing gene expression dynamics along the pseudotime. 10,325 genes with normalized expression ≥ 0.01 in at least 1% of cells were retained. For each gene *g*, the gene expression along pseudotime *t* in each sample *s* was described as a function ${f}_{gs}(t)$ which was obtained by fitting B-spline regression to the gene’s normalized expression values in single cells. The red curves in Fig. 4h are the mean of the function ${f}_{gs}(t)$ of the three samples. In order to test whether the gene expression shows a significant change along pseudotime, we compared the above model with a null model in which ${f}_{gs}(t)$ is assumed to be a constant over time. The likelihood ratio statistic between the two models was computed. To determine the *P* value, the null distribution of the likelihood ratio statistic was constructed by permuting the pseudotime of cells in each sample, refitting the models and recomputing the likelihood ratio statistic. The *P* value was calculated as the number of permutations out of a total of 1,000 permutations that produce a likelihood ratio statistic larger than the observed one. The *P* values from all genes were converted to FDR by Benjamini–Hochberg procedure to adjust for multiple testing. Genes with FDR < 0.05 were considered as dynamic genes with statistical significance. *k*-Means clustering was applied to group genes with similar dynamic expression patterns into clusters. topGO (v.2.42.0) was used to identify the enriched Gene Ontology terms by comparing the genes in each cluster to all 10,325 genes as background.

### Reporting summary

Further information on research design is available in the Nature Research Reporting Summary linked to this paper.

## Online content

Any methods, additional references, Nature Research reporting summaries, source data, extended data, supplementary information, acknowledgements, peer review information; details of author contributions and competing interests; and statements of data and code availability are available at 10.1038/s41586-021-03752-4.

## Supplementary information

Supplementary Information This file contains the full descriptions for Supplementary Data files 1-5, and the titles for Supplementary Tables 1-10.

Reporting Summary

Supplementary Data 1 This file contains differential gene lists in global CD3+ and refined CD8+ T cell UMAP clusters. See Supplementary Information PDF for full file description.

Supplementary Data 2 This file contains gene sets used to study transcriptional programs. See Supplementary Information PDF for full file description.

Supplementary Data 3 This file contains differential gene expression analysis comparing MPR (n=6) vs. non-MPR (n=9) by cell cluster and total CD4/CD8 TIL. See Supplementary Information PDF for full file description.

Supplementary Data 4 This file contains MANAFEST and ViraFEST assay results for each patient. See Supplementary Information PDF for full file description.

Supplementary Data 5 This file contains MANAFEST and ViraFEST assay graphs. See Supplementary Information PDF for full file description.

Supplementary Tables This file contains Supplementary Tables 1-10. See Supplementary Information PDF for descriptions.

## Data Availability

Bulk TCRVβ sequencing data generated by Adaptive Biotechnologies are available in the Adaptive Biotechnologies ImmuneACCESS repository under DOI 10.21417/JC2021N, at https://clients.adaptivebiotech.com/pub/caushi-2021-n. Bulk TCRVβ raw and processed sequencing data generated by the Sidney Kimmel Comprehensive Cancer Center FTIC are available in the Gene Expression Omnibus with accession number GSE173351. Raw scRNA-seq–TCR-seq data reported in this paper are available in the European Genome-phenome Archive under controlled access with accession number EGAS00001005343. Owing to the personal, sensitive and inherently identifying nature of raw genomic data, access to raw RNA-seq–TCR-seq data is controlled and full instructions to apply for data access can be found at https://ega-archive.org/access/data-access. Approvals will be granted immediately upon confirmation that all requirements are met. Processed and de-identified single-cell data are available in the Gene Expression Omnibus with accession number GSE176022.

## Code availability

Scripts to reproduce the analyses used in this study are available at https://github.com/BKI-immuno/neoantigen-specific-T-cells-NSCLC.

## References

[1] Tumeh PC (2014). PD-1 blockade induces responses by inhibiting adaptive immune resistance. Nature.

[2] Simoni Y (2018). Bystander CD8^+^ T cells are abundant and phenotypically distinct in human tumour infiltrates. Nature.

[3] Danilova L (2018). The mutation-associated neoantigen functional expansion of specific T Cells (MANAFEST) assay: a sensitive platform for monitoring antitumor immunity. Cancer Immunol. Res..

[4] Vogelstein B (2013). Cancer genome landscapes. Science.

[5] Yarchoan M, Hopkins A, Jaffee EM (2017). Tumor mutational burden and response rate to PD-1 inhibition. N. Engl. J. Med..

[6] Yarchoan M, Johnson BA, Lutz ER, Laheru DA, Jaffee EM (2017). Targeting neoantigens to augment antitumour immunity. Nat. Rev. Cancer.

[7] Scheper W (2019). Low and variable tumor reactivity of the intratumoral TCR repertoire in human cancers. Nat. Med..

[8] Forde PM (2018). Neoadjuvant PD-1 blockade in resectable lung cancer. N. Engl. J. Med..

[9] Blank CU (2018). Neoadjuvant versus adjuvant ipilimumab plus nivolumab in macroscopic stage III melanoma. Nat. Med..

[10] Huang AC (2019). A single dose of neoadjuvant PD-1 blockade predicts clinical outcomes in resectable melanoma. Nat. Med..

[11] Kaunitz GJ (2017). Melanoma subtypes demonstrate distinct PD-L1 expression profiles. Lab. Invest..

[12] Topalian SL (2020). Neoadjuvant nivolumab for patients with resectable Merkel cell carcinoma in the CheckMate 358 Trial. J. Clin. Oncol..

[13] Clarke J (2019). Single-cell transcriptomic analysis of tissue-resident memory T cells in human lung cancer. J. Exp. Med..

[14] Bagaev, D. V. et al. VDJdb in 2019: database extension, new analysis infrastructure and a T-cell receptor motif compendium. *Nucleic Acids Res*. **48**, D1057–D1062 (2020). 10.1093/nar/gkz874 PMC6943061 31588507

[15] Dykema AG (2021). Functional characterization of CD4^+^ T cell receptors crossreactive for SARS-CoV-2 and endemic coronaviruses. J. Clin. Invest..

[16] van der Leun AM, Thommen DS, Schumacher TN (2020). CD8^+^ T cell states in human cancer: insights from single-cell analysis. Nat. Rev. Cancer.

[17] Guo X (2018). Global characterization of T cells in non-small-cell lung cancer by single-cell sequencing. Nat. Med..

[18] Pizzolla A (2018). Influenza-specific lung-resident memory T cells are proliferative and polyfunctional and maintain diverse TCR profiles. J. Clin. Invest..

[19] Pearce EL (2003). Control of effector CD8^+^ T cell function by the transcription factor eomesodermin. Science.

[20] Chihara N (2018). Induction and transcriptional regulation of the co-inhibitory gene module in T cells. Nature.

[21] Scott AC (2019). TOX is a critical regulator of tumour-specific T cell differentiation. Nature.

[22] Yao C (2019). Single-cell RNA-seq reveals TOX as a key regulator of CD8^+^ T cell persistence in chronic infection. Nat. Immunol..

[23] Seo H (2019). TOX and TOX2 transcription factors cooperate with NR4A transcription factors to impose CD8^+^ T cell exhaustion. Proc. Natl Acad. Sci. USA.

[24] Mackay LK (2016). Hobit and Blimp1 instruct a universal transcriptional program of tissue residency in lymphocytes. Science.

[25] Kok L (2020). A committed tissue-resident memory T cell precursor within the circulating CD8^+^ effector T cell pool. J. Exp. Med..

[26] Topalian SL, Taube JM, Pardoll DM (2020). Neoadjuvant checkpoint blockade for cancer immunotherapy. Science.

[27] Behr FM (2020). Tissue-resident memory CD8^+^ T cells shape local and systemic secondary T cell responses. Nat. Immunol..

[28] Skoulidis F (2018). *STK11/LKB1* mutations and PD-1 inhibitor resistance in *KRAS*-mutant lung adenocarcinoma. Cancer Discov..

[29] Smith KN (2019). Persistent mutant oncogene specific T cells in two patients benefitting from anti-PD-1. J. Immunother. Cancer.

[30] Anagnostou V (2017). Evolution of neoantigen landscape during immune checkpoint blockade in non-small cell lung cancer. Cancer Discov..

[31] Cottrell TR (2018). Pathologic features of response to neoadjuvant anti-PD-1 in resected non-small-cell lung carcinoma: a proposal for quantitative immune-related pathologic response criteria (irPRC). Ann. Oncol..

[32] Anagnostou V (2020). Multimodal genomic features predict outcome of immune checkpoint blockade in non-small-cell lung cancer. Nat. Cancer.

[33] Jones S (2015). Personalized genomic analyses for cancer mutation discovery and interpretation. Sci. Transl. Med..

[34] Carlson CS (2013). Using synthetic templates to design an unbiased multiplex PCR assay. Nat. Commun..

[35] Harndahl M (2009). Peptide binding to HLA class I molecules: homogenous, high-throughput screening, and affinity assays. J. Biomol. Screen..

[36] Sylvester-Hvid C (2002). Establishment of a quantitative ELISA capable of determining peptide - MHC class I interaction. Tissue Antigens.

[37] O’Flanagan CH (2019). Dissociation of solid tumor tissues with cold active protease for single-cell RNA-seq minimizes conserved collagenase-associated stress responses. Genome Biol..

[38] van den Brink SC (2017). Single-cell sequencing reveals dissociation-induced gene expression in tissue subpopulations. Nat. Methods.

[39] Li H (2020). Dysfunctional CD8 T cells form a proliferative, dynamically regulated compartment within human melanoma. Cell.

[40] Stuart T (2019). Comprehensive integration of single-cell data. Cell.

[41] Huang M (2018). SAVER: gene expression recovery for single-cell RNA sequencing. Nat. Methods.

[42] Leek JT, Johnson WE, Parker HS, Jaffe AE, Storey JD (2012). The sva package for removing batch effects and other unwanted variation in high-throughput experiments. Bioinformatics.

[43] Johnson WE, Li C, Rabinovic A (2007). Adjusting batch effects in microarray expression data using empirical Bayes methods. Biostatistics.

[44] Hotelling, H. *Relations Between Two Sets of Variates* Vol. 28, 321–377 (Biometrika, 1936).

[45] Härdle, W. K. & Simar, L. *Applied Multivariate Statistical Analysis* 1st edn (Springer-Verlag, 2003).

[46] McCulloch, C. E., Searle, S. R. & Neuhaus, J. M. *Generalized, Linear and Mixed Models* 2nd edn (Wiley, 2008).

[47] Benjamini Y, Hochberg Y (1995). Controlling the false discovery rate: a practical and powerful approach to multiple testing. J. R. Stat. Soc. B.

[48] Belarif L (2018). IL-7 receptor blockade blunts antigen-specific memory T cell responses and chronic inflammation in primates. Nat. Commun..

