# Author Correction: Transcriptional programs of neoantigen-specific TIL in anti-PD-1-treated lung cancers

**DOI:** 10.1038/s41586-021-03893-6

**Published:** 2021-10-04

**Authors:** Justina X. Caushi, Jiajia Zhang, Zhicheng Ji, Ajay Vaghasia, Boyang Zhang, Emily Han-Chung Hsiue, Brian J. Mog, Wenpin Hou, Sune Justesen, Richard Blosser, Ada Tam, Valsamo Anagnostou, Tricia R. Cottrell, Haidan Guo, Hok Yee Chan, Dipika Singh, Sampriti Thapa, Arbor G. Dykema, Poromendro Burman, Begum Choudhury, Luis Aparicio, Laurene S. Cheung, Mara Lanis, Zineb Belcaid, Margueritta El Asmar, Peter B. Illei, Rulin Wang, Jennifer Meyers, Kornel Schuebel, Anuj Gupta, Alyza Skaist, Sarah Wheelan, Jarushka Naidoo, Kristen A. Marrone, Malcolm Brock, Jinny Ha, Errol L. Bush, Bernard J. Park, Matthew Bott, David R. Jones, Joshua E. Reuss, Victor E. Velculescu, Jamie E. Chaft, Kenneth W. Kinzler, Shibin Zhou, Bert Vogelstein, Janis M. Taube, Matthew D. Hellmann, Julie R. Brahmer, Taha Merghoub, Patrick M. Forde, Srinivasan Yegnasubramanian, Hongkai Ji, Drew M. Pardoll, Kellie N. Smith

**Affiliations:** 1 Bloomberg~Kimmel Institute for Cancer Immunotherapy at Johns Hopkins, Baltimore, MD USA; 2 The Mark Center for Advanced Genomics and Imaging at Johns Hopkins, Baltimore, MD USA; 3 Sidney Kimmel Comprehensive Cancer Center at Johns Hopkins, Baltimore, MD USA; 4 Department of Biostatistics, Bloomberg School of Public Health, Johns Hopkins University, Baltimore, MD USA; 5 Ludwig Center and Howard Hughes Medical Institute at Johns Hopkins, Baltimore, MD USA; 6 Lustgarten Pancreatic Cancer Research Laboratory, Sidney Kimmel Comprehensive Cancer Center, Johns Hopkins University, Baltimore, MD USA; 7 Immunitrack, Copenhagen, Denmark; 8 Department of Medicine, Memorial Sloan Kettering Cancer Center and Weill Cornell Medicine, New York, NY USA; 9 The Swim Across America and Ludwig Collaborative Laboratory, Immunology Program, Parker Institute for Cancer Immunotherapy at Memorial Sloan Kettering Cancer Center, New York, NY USA; 10 Human Oncology and Pathogenesis Program, Memorial Sloan Kettering Cancer Center, New York, NY USA; 11 Present Address: Department of Biostatistics and Bioinformatics, Duke University School of Medicine, Durham, NC USA; 12 Present Address: Ontario Institute for Cancer Research, Queens University, Kingston, Ontario Canada; 13 Present Address: Beaumont Hospital Dublin, RCSI University of Medicine and Health Science, Dublin, Ireland; 14 Present Address: Georgetown Lombardi Comprehensive Cancer Center at Georgetown University, Washington, DC USA

**Keywords:** Cellular immunity, Immunotherapy, CD8-positive T cells, T-cell receptor, Tumour immunology

Correction to: *Nature* 10.1038/s41586-021-03752-4 Published online 21 July 2021

In the originally published version of this Article there were three labeling errors in Fig. 1. The cluster names for “CD8-MHCII” and “CD8-proliferating” were switched and the cluster names for “Stem-like memory” and “MAIT” were switched (Fig. 1c). Their positions have now been corrected. In Fig. 1d, a typo in the “*SCL4A10*” gene label has been corrected to “*SLC4A10*.” The legend for Fig. 1c originally referred to the heatmap as displaying the “top-5” most differential genes. This has been corrected to “the top-3” most differential genes. In the eighth paragraph of main text, a typo in the gene name “*LINC02246*” has been corrected to “*LINC02446*.” Further, in Supplementary Table 9, the data point “5” was missing in the Number of nonsynonymous mutations per exome column for patient NY016-007; this has now been corrected. The original Article has been corrected online.

