# Supplementary material for: Transcriptional programs of neoantigen-specific TIL in anti-PD-1-treated lung cancers
Source: Nature. 2021 Jul 21;596(7870):126–32. doi: 10.1038/s41586-021-03752-4 (PMC8338555; doi:10.1038/s41586-021-03752-4)
Supplement: Supplementary file 1 — This file contains the full descriptions for Supplementary Data files 1-5, and the titles for Supplementary Tables 1-10. [file 41586_2021_3752_MOESM1_ESM.pdf]

nature portfolio

https://doi.org/10.1038/s41586-021-03752-4

Supplementary information

Transcriptional programs of neoantigen-specific TIL in anti-PD-1-treated lung cancers

In the format provided by the authors and unedited

Distinct transcriptional programs characterize neoantigen-specific TIL in lung cancers treated with anti-PD-1

Caushi JX and Zhang J et al.

Supplementary Information Guide

1. **Supplementary Data 1. Differential gene lists in global CD3+ and refined CD8+ T cell UMAP clusters.**
    a. Supplementary Data 1.1 – Complete list of differential genes in each cluster of the CD3+ T cell UMAP
    b. Supplementary Data 1.2 – Complete list of differential genes in each cluster of the CD8+ T cell UMAP
    c. Supplementary Data 1.3 – Complete list of differential genes comparing MANA- vs. EBV- vs. flu-specific TIL
    d. Supplementary Data 1.4 – Complete list of differential genes comparing MANA-specific T cells from MPR and non-MPR
    e. Supplementary Data 1.5 – Complete list of differential genes comparing MANA-specific T cells of primary tumor vs. a distant brain metastasis from non-MPR MD043-011

2. **Supplementary Data 2. Gene sets used to study transcriptional programs.**
    a. Supplementary Data 2.1 – Core TRM gene set as published by Clarke, et. al.
    b. Supplementary Data 2.2 – Exhausted T cell gene set as published by Zhang et. al.
    c. Supplementary Data 2.3 – IL-7-induced gene set as published by Belarif et. al.

3. **Supplementary Data 3 – Differential gene expression analysis comparing MPR (n=6) vs. non-MPR (n=9) by cell cluster and total CD4/CD8 TIL.** The differential genes between MPR and non-MPR are shown for each T cell cluster. Each tab of the excel file corresponds to a cluster in the global CD3+ T cell UMAP.

4. **Supplementary Data 4. MANAFEST and ViraFEST assay results for each patient.** Each tab represent a patient for whom MANAFEST/ViraFEST was performed. Column 1 indicates the antigen-specific TCRs identified by the bioinformatic platform and row 1 indicates the antigen tested in the assay (refer to Supplementary Table 6). Each cell indicates the frequency of each TCR (as indicated in column 1) in the relevant peptide-stimulated well (as indicated in row 1).

5. **Supplementary Data 5. MANAFEST and ViraFEST assay graphs.**
    a. **Supplementary Data 5.1 - MANA-specific TCRs identified in MPR and non-MPR patients using the MANAFEST assays.** Antigen-specific responses identified using the MANAFEST assay are shown for MPRs MD01-005 (re-analyzed results are shown below; original results previously reported for this patient in Forde PM et al, N Engl J Med, 2018), MD043-008, MD043-003, and NY016-025. Antigen-specific responses identified using the MANAFEST assay are shown for non-MPRs MD043-011, NY016-007, and NY016-014. Each antigen-specific clonotypic expansion is color coded to indicate if the clone was not detected in the single cell data (blue), detected in the single cell data but not tested via TCR cloning (green), detected in the single cell data but did not validate with TCR cloning (orange), or detected in the single cell data and validated with TCR cloning (red). Data are shown as the percent of MANAFEST+ clonotypes among CD8+ T cells after 10 day culture. Neither MANA-specific nor CEF-specific T cell responses were detected in non-MPR patient, MD01-019. *Clones determined to recognize CEF were queried against a database of TCRs with known specificity (vdjdb, Supplementary Table 8). Several clonotypes were previously been reported to recognize common viral epitopes used in the CEF pool. Two of these clonotypes reported to recognize the influenza A matrix protein HLA A*02:01-restricted GILGFVFTL epitope. The full TCR corresponding to these clonotypes were cloned into the Jurkat transfer system and were validated to recognize the

reported influenza antigen. Composite 3D bar plot for MD01-004 is shown in **Extended Data Fig. 2**.

b. **Supplementary Data 5.2. Influenza-specific TCRs identified using the ViraFEST assay.** The ViraFEST assay was performed on non-MPR patients MD01-004 and MD043-011, and MPR patient MD01-005 to identify influenza-specific TCRβ clones. Influenza A pools consisting of overlapping peptides from the matrix and nucleocapsid proteins of H1N1 and H3N2, or an HIV-1 Gag pool as a negative control, were used to stimulate peripheral blood T cells in vitro for 10 days. Each flu peptide pool was tested in triplicate. Data are shown as the frequency of ViraFEST+ clonotypes among total $CD8^+$ T cells for each culture condition. Clonotypes are color coded to indicate if they were not detected in single cell TIL (blue) or were detected in single cell TIL (green).

c. **Supplementary Data 5.3. The frequency of each MANA-specific TCR Vβ clonotype in each condition tested in the MANAFEST assay visualized by individual dot plots.** Peripheral blood T cells were tested for reactivity to putative MANA using the MANAFEST assay. A graph is shown for each MANA- and CEF-specific TCR Vβ clonotype (right y-axis) detected in each patient. The frequency of each clonotype is shown as the percent among all T cells detected by TCRseq after culture (left y-axis) in each peptide-stimulated condition, the CEF positive control well, and the "no peptide" negative control well (x-axis).

6. **Supplementary Tables**
   a. Supplementary Table 1. Clinical and histopathological features of patients included in the study
   b. Supplementary Table 2. Comprehensive list of biospecimens used for each analysis
   c. Supplementary Table 3. Single cell TCR/RNA sequencing information and metrics
   d. Supplementary Table 4. Summary of next-generation whole exome sequencing analysis
   e. Supplementary Table 5. Somatic sequence alterations.
   f. Supplementary Table 6. Putative MANA tested by MANAFEST
   g. Supplementary Table 7. MANAFEST TCR sequencing summary statistics
   h. Supplementary Table 8. Antigen-specific TCR clonotypes identified by the MANAFEST and ViraFEST assays
   i. Supplementary Table 9. MANAFEST assay results summary
   j. Supplementary Table 10. MANA-specific TCR cloning and tumor representation
