# Supplementary material for: Transcriptional programs of neoantigen-specific TIL in anti-PD-1-treated lung cancers
Source: Nature. 2021 Jul 21;596(7870):126–32. doi: 10.1038/s41586-021-03752-4 (PMC8338555; doi:10.1038/s41586-021-03752-4)
Supplement: Supplementary file 2 — Reporting Summary [file 41586_2021_3752_MOESM2_ESM.pdf]

nature research

Corresponding author(s): Kellie N. Smith, Drew M. Pardoll, Hongkai Ji, Srinivasan Yegnasubramanian

Last updated by author(s): 2021/06/17

Reporting Summary

Nature Research wishes to improve the reproducibility of the work that we publish. This form provides structure for consistency and transparency in reporting. For further information on Nature Research policies, see our Editorial Policies and the Editorial Policy Checklist.

Statistics

For all statistical analyses, confirm that the following items are present in the figure legend, table legend, main text, or Methods section.

n/a	Confirmed	
☐	☒	The exact sample size (*n*) for each experimental group/condition, given as a discrete number and unit of measurement
☐	☒	A statement on whether measurements were taken from distinct samples or whether the same sample was measured repeatedly
☐	☒	The statistical test(s) used AND whether they are one- or two-sided<br>*Only common tests should be described solely by name; describe more complex techniques in the Methods section.*
☐	☒	A description of all covariates tested
☐	☒	A description of any assumptions or corrections, such as tests of normality and adjustment for multiple comparisons
☐	☒	A full description of the statistical parameters including central tendency (e.g. means) or other basic estimates (e.g. regression coefficient) AND variation (e.g. standard deviation) or associated estimates of uncertainty (e.g. confidence intervals)
☐	☒	For null hypothesis testing, the test statistic (e.g. *F*, *t*, *r*) with confidence intervals, effect sizes, degrees of freedom and *P* value noted<br>*Give P values as exact values whenever suitable.*
☒	☐	For Bayesian analysis, information on the choice of priors and Markov chain Monte Carlo settings
☒	☐	For hierarchical and complex designs, identification of the appropriate level for tests and full reporting of outcomes
☐	☒	Estimates of effect sizes (e.g. Cohen's *d*, Pearson's *r*), indicating how they were calculated

Our web collection on statistics for biologists contains articles on many of the points above.

Software and code

Policy information about availability of computer code

Data collection	No software was used for data collection
Data analysis	Alignment and preprocessing was performed using cellranger 3.1.0 (10X Genomics).<br>Data were processed using Seurat 3.1.5 in R 4.0.3. Gene expression heatmap was generated using heatmap3 1.1.7 package. Plots were generated using PRISM 8.2.0 and ggplot2 3.3.2 package in R. Bioinformatic identification of antigen specific T cells in bulk TCR sequencing was performed in http://www.stat-apps.onc.jhmi.edu/FEST/. Scripts to reproduce the analyses used in this study are available at: https://github.com/BKI-immuno/neoantigen-specific-T-cells-NSCLC.

For manuscripts utilizing custom algorithms or software that are central to the research but not yet described in published literature, software must be made available to editors and reviewers. We strongly encourage code deposition in a community repository (e.g. GitHub). See the Nature Research guidelines for submitting code & software for further information.

Data

Policy information about availability of data

All manuscripts must include a data availability statement. This statement should provide the following information, where applicable:

- Accession codes, unique identifiers, or web links for publicly available datasets
- A list of figures that have associated raw data
- A description of any restrictions on data availability

Bulk TCR Vbeta sequencing data generated by Adaptive Biotechnologies is available in the Adaptive Biotechnologies ImmuneACCESS repository under DOI 10.21417/JC2021N, at clients.adaptivebiotech.com/pub/caushi-2021-n. Bulk TCR Vbeta raw and processed sequencing data generated by the SKCCC FTIC are available in GEO with accession number GSE173351. Raw scRNAseq/TCRseq data reported in this paper are available in the European Genome-phenome archive

under controlled access with accession number EGAS00001005343. Due to the personal, sensitive and inherently identifying nature of raw genomic data, access to rawRNAseq/TCRseq data are controlled and full instructions to apply for data access can be found at https://ega-archive.org/access/data-access. Approvals will be granted immediately upon confirmation that all requirements are met. Processed and de-identified single cell data are available in GEO with accession number GSE176022. Somatic mutations, predicted neoantigens, and the identity of all antigen-specific TCR Vbeta clonotypes are shown in the Supplementary Tables.

Field-specific reporting

Please select the one below that is the best fit for your research. If you are not sure, read the appropriate sections before making your selection.

☒ Life sciences ☐ Behavioural & social sciences ☐ Ecological, evolutionary & environmental sciences

For a reference copy of the document with all sections, see nature.com/documents/nr-reporting-summary-flat.pdf

Life sciences study design

All studies must disclose on these points even when the disclosure is negative.

Sample size	21 patients with non-small cell lung cancer were recruited to a prospective phase 2 clinical trial that investigated the safety and feasibility of administering two doses of anti-PD-1 (nivolumab) prior to surgical resection. Twenty patients had surgical resection of their primary tumor. Major pathologic response (MPR) was defined as ≤ 10% residual tumor as assessed by two independent pathologists. All participants signed the informed consent. In total, we performed combined single cell RNA/TCR sequencing of 16 patients (7 MPR, 9 non-MPR) and 9 patients were tested for MANA reactivity (4 MPR, 5 non-MPR).
Data exclusions	Single cell sequencing was performed on all tumor and normal lung specimens with sufficient viably banked T cells. MANAFEST/ViraFest was performed on all patients with adequate blood samples and available WES. Single cell TCRseq/RNAseq was performed on peripheral blood T cells sorted for positive expression of the TCR Vb2 gene. Unconventional/MAIT CD8+ T cells were excluded from this analysis to enable better visualization of canonical CD8+ T cell subsets.
Replication	Epidemiological replication/validation: In this study, we included all NSCLC patients enrolled at two academic cancer centers in the US, who were willing to participate in this study and signed the informed consent. For replication purposes an additional validation cohort would have been desirable. However, due to the limited number of patients in a phase 2 study per study design and increased interests to test combinational regimens in the neoadjuvant setting, we were not able to perform an independent replication trial.<br>Technical replication: We performed single-cell RNA sequencing experiments with high numbers of cells per patient and, in general, high quality sequencing libraries. Sample replicates were performed for 4 patients and showed high consistency in RNA expression profile, and were subsequently merged as one single sample for downstream analysis.
Randomization	*Randomization was not relevant for our study*
Blinding	*Blinding was not relevant for our study*

Behavioural & social sciences study design

All studies must disclose on these points even when the disclosure is negative.

Study description	*Briefly describe the study type including whether data are quantitative, qualitative, or mixed-methods (e.g. qualitative cross-sectional, quantitative experimental, mixed-methods case study).*
Research sample	*State the research sample (e.g. Harvard university undergraduates, villagers in rural India) and provide relevant demographic information (e.g. age, sex) and indicate whether the sample is representative. Provide a rationale for the study sample chosen. For studies involving existing datasets, please describe the dataset and source.*
Sampling strategy	*Describe the sampling procedure (e.g. random, snowball, stratified, convenience). Describe the statistical methods that were used to predetermine sample size OR if no sample-size calculation was performed, describe how sample sizes were chosen and provide a rationale for why these sample sizes are sufficient. For qualitative data, please indicate whether data saturation was considered, and what criteria were used to decide that no further sampling was needed.*
Data collection	*Provide details about the data collection procedure, including the instruments or devices used to record the data (e.g. pen and paper, computer, eye tracker, video or audio equipment) whether anyone was present besides the participant(s) and the researcher, and whether the researcher was blind to experimental condition and/or the study hypothesis during data collection.*
Timing	*Indicate the start and stop dates of data collection. If there is a gap between collection periods, state the dates for each sample cohort.*
Data exclusions	*If no data were excluded from the analyses, state so OR if data were excluded, provide the exact number of exclusions and the rationale behind them, indicating whether exclusion criteria were pre-established.*
Non-participation	*State how many participants dropped out/declined participation and the reason(s) given OR provide response rate OR state that no participants dropped out/declined participation.*

Randomization	*If participants were not allocated into experimental groups, state so OR describe how participants were allocated to groups, and if allocation was not random, describe how covariates were controlled.*

Ecological, evolutionary & environmental sciences study design

All studies must disclose on these points even when the disclosure is negative.

Study description	*Briefly describe the study. For quantitative data include treatment factors and interactions, design structure (e.g. factorial, nested, hierarchical), nature and number of experimental units and replicates.*
Research sample	*Describe the research sample (e.g. a group of tagged Passer domesticus, all Stenocereus thurberi within Organ Pipe Cactus National Monument), and provide a rationale for the sample choice. When relevant, describe the organism taxa, source, sex, age range and any manipulations. State what population the sample is meant to represent when applicable. For studies involving existing datasets, describe the data and its source.*
Sampling strategy	*Note the sampling procedure. Describe the statistical methods that were used to predetermine sample size OR if no sample-size calculation was performed, describe how sample sizes were chosen and provide a rationale for why these sample sizes are sufficient.*
Data collection	*Describe the data collection procedure, including who recorded the data and how.*
Timing and spatial scale	*Indicate the start and stop dates of data collection, noting the frequency and periodicity of sampling and providing a rationale for these choices. If there is a gap between collection periods, state the dates for each sample cohort. Specify the spatial scale from which the data are taken*
Data exclusions	*If no data were excluded from the analyses, state so OR if data were excluded, describe the exclusions and the rationale behind them, indicating whether exclusion criteria were pre-established.*
Reproducibility	*Describe the measures taken to verify the reproducibility of experimental findings. For each experiment, note whether any attempts to repeat the experiment failed OR state that all attempts to repeat the experiment were successful.*
Randomization	*Describe how samples/organisms/participants were allocated into groups. If allocation was not random, describe how covariates were controlled. If this is not relevant to your study, explain why.*
Blinding	*Describe the extent of blinding used during data acquisition and analysis. If blinding was not possible, describe why OR explain why blinding was not relevant to your study.*

Did the study involve field work? ☐ Yes ☐ No

Field work, collection and transport

Field conditions	*Describe the study conditions for field work, providing relevant parameters (e.g. temperature, rainfall).*
Location	*State the location of the sampling or experiment, providing relevant parameters (e.g. latitude and longitude, elevation, water depth).*
Access & import/export	*Describe the efforts you have made to access habitats and to collect and import/export your samples in a responsible manner and in compliance with local, national and international laws, noting any permits that were obtained (give the name of the issuing authority, the date of issue, and any identifying information).*
Disturbance	*Describe any disturbance caused by the study and how it was minimized.*

Reporting for specific materials, systems and methods

We require information from authors about some types of materials, experimental systems and methods used in many studies. Here, indicate whether each material, system or method listed is relevant to your study. If you are not sure if a list item applies to your research, read the appropriate section before selecting a response.

Materials & experimental systems

n/a	Involved in the study	
☐	☒	Antibodies
☐	☒	Eukaryotic cell lines
☒	☐	Palaeontology and archaeology
☐	☒	Animals and other organisms
☐	☒	Human research participants
☐	☒	Clinical data
☒	☐	Dual use research of concern

Methods

n/a	Involved in the study	
☒	☐	ChIP-seq
☐	☒	Flow cytometry
☒	☐	MRI-based neuroimaging

Antibodies

Antibodies used	LIVE/DEAD™ Fixable Near-IR; ThermoFisher<br>CD3-BV605, clone SK7; BD Bioscience<br>LIVE/DEAD™ Fixable Aqua; ThermoFisher<br>CD3-PE, clone HIT3a; BD Bioscience<br>CD8-BV786, clone RPA-T8; BD Bioscience<br>Anti-TCR Vb2-PE, clone REA654; Miltenyi<br>Anti-TCR Vb5-FITC, clone MEM-262, Biolegend
Validation	All new antibodies were titrated to determine the optimal working concentration. Specificity was validated using control primary cells or cell lines. Isotype controls were used to gate on cells staining with the antibody of interest.

Eukaryotic cell lines

Policy information about cell lines

Cell line source(s)	Jurkat cell line - Promega
Authentication	TCRa/b knockout of the jurkat cell line was confirmed by Sanger sequencing and restoration of CD3 expression only by the co-transfection of TCRα or TCRβ chains.
Mycoplasma contamination	The jurkat cell line used in this study tested negative for mycoplasma contamination
Commonly misidentified lines<br>(See ICLAC register)	We did not use any commonly misidentified cell lines

Palaeontology and Archaeology

Specimen provenance	*Provide provenance information for specimens and describe permits that were obtained for the work (including the name of the issuing authority, the date of issue, and any identifying information).*
Specimen deposition	*Indicate where the specimens have been deposited to permit free access by other researchers.*
Dating methods	*If new dates are provided, describe how they were obtained (e.g. collection, storage, sample pretreatment and measurement), where they were obtained (i.e. lab name), the calibration program and the protocol for quality assurance OR state that no new dates are provided.*

☐ Tick this box to confirm that the raw and calibrated dates are available in the paper or in Supplementary Information.

Ethics oversight	*Identify the organization(s) that approved or provided guidance on the study protocol, OR state that no ethical approval or guidance was required and explain why not.*

Note that full information on the approval of the study protocol must also be provided in the manuscript.

Animals and other organisms

Policy information about studies involving animals; ARRIVE guidelines recommended for reporting animal research

Laboratory animals	*For laboratory animals, report species, strain, sex and age OR state that the study did not involve laboratory animals.*
Wild animals	*Provide details on animals observed in or captured in the field; report species, sex and age where possible. Describe how animals were caught and transported and what happened to captive animals after the study (if killed, explain why and describe method; if released, say where and when) OR state that the study did not involve wild animals.*
Field-collected samples	*For laboratory work with field-collected samples, describe all relevant parameters such as housing, maintenance, temperature, photoperiod and end-of-experiment protocol OR state that the study did not involve samples collected from the field.*
Ethics oversight	*Identify the organization(s) that approved or provided guidance on the study protocol, OR state that no ethical approval or guidance was required and explain why not.*

Note that full information on the approval of the study protocol must also be provided in the manuscript.

Human research participants

Policy information about studies involving human research participants

Population characteristics	No patient was excluded from the original clinical trial on the basis of sex, ethnic background, or socio-economic status. Only patients greater than age 18 were eligible for the study. Patients ranged in age from 55 to 84. Health status was good to excellent and the breakdown for race was 5.7% Hispanic, 5.7% African American, 8.6% Other, and 80% Caucasian. Special classes of subjects such as pregnant women, children, prisoners, or other institutionalized individuals were not included in

	the original trial and are therefore not included in our present study.
Recruitment	Participants were not prospectively recruited for this study. The original clinical trial was performed on 21 patients with resectable NSCLC. Twenty of these patients underwent successful surgical resection of their primary tumor. Patients included in the present study were selected solely based on the availability of biospecimens for analysis.
Ethics oversight	This study was approved by the Institutional Review Boards of Johns Hopkins and MSKCC

Note that full information on the approval of the study protocol must also be provided in the manuscript.

Clinical data

Policy information about clinical studies

All manuscripts should comply with the ICMJE guidelines for publication of clinical research and a completed CONSORT checklist must be included with all submissions.

Clinical trial registration	NCT02259621
Study protocol	The results from this clinical trial and associated study protocol have been published previously (Forde PM, et al, N Engl J Med, 2018). The study protocol can be accessed at: https://www.nejm.org/doi/suppl/10.1056/NEJMoa1716078/suppl_file/nejmoa1716078_protocol.pdf
Data collection	All biospecimens were obtained between 2015 and 2018 from patients enrolled to a phase II study of neoadjuvant PD-1 blockade in resectable lung cancer at Johns Hopkins Sidney Kimmel Comprehensive Cancer Center and Memorial Sloan Kettering Cancer Center. Analyses on these biospecimens were continuously performed between 2017 and 2021.
Outcomes	Primary lung tumor and lymph-node surgical specimens were staged according to the criteria of the American Joint Committee on Cancer (seventh edition) for evaluating tumor size, affected lymph nodes, and metastases.7 Primary tumors were assessed for the percentage of residual viable tumor that was identified on routine hematoxylin and eosin staining, and tumors with no more than 10% viable tumor cells were considered to have had a major pathological response. These methods and clinical outcomes have been reported previously (Forde PM, et al, N Engl J Med, 2018).

Dual use research of concern

Policy information about dual use research of concern

Hazards

Could the accidental, deliberate or reckless misuse of agents or technologies generated in the work, or the application of information presented in the manuscript, pose a threat to:

No	Yes	
☒	☐	Public health
☒	☐	National security
☒	☐	Crops and/or livestock
☒	☐	Ecosystems
☒	☐	Any other significant area

Experiments of concern

Does the work involve any of these experiments of concern:

No	Yes	
☒	☐	Demonstrate how to render a vaccine ineffective
☒	☐	Confer resistance to therapeutically useful antibiotics or antiviral agents
☒	☐	Enhance the virulence of a pathogen or render a nonpathogen virulent
☒	☐	Increase transmissibility of a pathogen
☒	☐	Alter the host range of a pathogen
☒	☐	Enable evasion of diagnostic/detection modalities
☒	☐	Enable the weaponization of a biological agent or toxin
☒	☐	Any other potentially harmful combination of experiments and agents

ChIP-seq

Data deposition

☐ Confirm that both raw and final processed data have been deposited in a public database such as GEO.

☐ Confirm that you have deposited or provided access to graph files (e.g. BED files) for the called peaks.

Data access links<br>*May remain private before publication.*	*For "Initial submission" or "Revised version" documents, provide reviewer access links. For your "Final submission" document, provide a link to the deposited data.*
Files in database submission	*Provide a list of all files available in the database submission.*
Genome browser session<br>(e.g. UCSC)	*Provide a link to an anonymized genome browser session for "Initial submission" and "Revised version" documents only, to enable peer review. Write "no longer applicable" for "Final submission" documents.*

Methodology

Replicates	*Describe the experimental replicates, specifying number, type and replicate agreement.*
Sequencing depth	*Describe the sequencing depth for each experiment, providing the total number of reads, uniquely mapped reads, length of reads and whether they were paired- or single-end.*
Antibodies	*Describe the antibodies used for the ChIP-seq experiments; as applicable, provide supplier name, catalog number, clone name, and lot number.*
Peak calling parameters	*Specify the command line program and parameters used for read mapping and peak calling, including the ChIP, control and index files used.*
Data quality	*Describe the methods used to ensure data quality in full detail, including how many peaks are at FDR 5% and above 5-fold enrichment.*
Software	*Describe the software used to collect and analyze the ChIP-seq data. For custom code that has been deposited into a community repository, provide accession details.*

Flow Cytometry

Plots

Confirm that:

☒ The axis labels state the marker and fluorochrome used (e.g. CD4-FITC).

☒ The axis scales are clearly visible. Include numbers along axes only for bottom left plot of group (a 'group' is an analysis of identical markers).

☒ All plots are contour plots with outliers or pseudocolor plots.

☒ A numerical value for number of cells or percentage (with statistics) is provided.

Methodology

Sample preparation	Cells were resuspended in PBS and stained with a viability marker (LIVE/DEAD™ Fixable Near-IR; ThermoFisher) for 15mins at RT in the dark. Cells were the incubated with FC block for 15 mins on ice and stained with antibody against CD3 (BV605, clone SK7) for 30mins on ice. After staining, highly viable CD3+ T cells were sorted into 0.04% BSA in PBS using a BD FACSAria II Cell Sorter. Sorted cells were manually counted using a hemocytometer and prepared at the desired cell concentration (1000 cells/ul), when possible. CD3+ cells were then sorted and immediately used in single cell experiments.
Instrument	FACS Aria II
Software	FACS Diva
Cell population abundance	Cells that were FACS sorted were immediately used in single cell TCRseq/RNAseq experiments. Only T cells were sequenced in this platform, and the cells analyzed were validated using gene expression programs, so there is no risk of contaminating cell types/phenotypes.
Gating strategy	Gating was based on staining with negative isotype control antibodies

☒ Tick this box to confirm that a figure exemplifying the gating strategy is provided in the Supplementary Information.

Magnetic resonance imaging

Experimental design

Design type	*Indicate task or resting state; event-related or block design.*
Design specifications	*Specify the number of blocks, trials or experimental units per session and/or subject, and specify the length of each trial or block (if trials are blocked) and interval between trials.*
Behavioral performance measures	*State number and/or type of variables recorded (e.g. correct button press, response time) and what statistics were used to establish that the subjects were performing the task as expected (e.g. mean, range, and/or standard deviation across subjects).*

Acquisition

Imaging type(s)	*Specify: functional, structural, diffusion, perfusion.*
Field strength	*Specify in Tesla*
Sequence & imaging parameters	*Specify the pulse sequence type (gradient echo, spin echo, etc.), imaging type (EPI, spiral, etc.), field of view, matrix size, slice thickness, orientation and TE/TR/flip angle.*
Area of acquisition	*State whether a whole brain scan was used OR define the area of acquisition, describing how the region was determined.*

Diffusion MRI ☐ Used ☐ Not used

Preprocessing

Preprocessing software	*Provide detail on software version and revision number and on specific parameters (model/functions, brain extraction, segmentation, smoothing kernel size, etc.).*
Normalization	*If data were normalized/standardized, describe the approach(es): specify linear or non-linear and define image types used for transformation OR indicate that data were not normalized and explain rationale for lack of normalization.*
Normalization template	*Describe the template used for normalization/transformation, specifying subject space or group standardized space (e.g. original Talairach, MNI305, ICBM152) OR indicate that the data were not normalized.*
Noise and artifact removal	*Describe your procedure(s) for artifact and structured noise removal, specifying motion parameters, tissue signals and physiological signals (heart rate, respiration).*
Volume censoring	*Define your software and/or method and criteria for volume censoring, and state the extent of such censoring.*

Statistical modeling & inference

Model type and settings	*Specify type (mass univariate, multivariate, RSA, predictive, etc.) and describe essential details of the model at the first and second levels (e.g. fixed, random or mixed effects; drift or auto-correlation).*
Effect(s) tested	*Define precise effect in terms of the task or stimulus conditions instead of psychological concepts and indicate whether ANOVA or factorial designs were used.*

Specify type of analysis: ☐ Whole brain ☐ ROI-based ☐ Both

Statistic type for inference (See Eklund et al. 2016)	*Specify voxel-wise or cluster-wise and report all relevant parameters for cluster-wise methods.*
Correction	*Describe the type of correction and how it is obtained for multiple comparisons (e.g. FWE, FDR, permutation or Monte Carlo).*

Models & analysis

n/a	Involved in the study
☐	☐ Functional and/or effective connectivity
☐	☐ Graph analysis
☐	☐ Multivariate modeling or predictive analysis

Functional and/or effective connectivity	*Report the measures of dependence used and the model details (e.g. Pearson correlation, partial correlation, mutual information).*
Graph analysis	*Report the dependent variable and connectivity measure, specifying weighted graph or binarized graph, subject- or group-level, and the global and/or node summaries used (e.g. clustering coefficient, efficiency, etc.).*

Multivariate modeling and predictive analysis

Specify independent variables, features extraction and dimension reduction, model, training and evaluation metrics.
