# Supplementary material for: Transcriptional programs of neoantigen-specific TIL in anti-PD-1-treated lung cancers
Source: Nature. 2021 Jul 21;596(7870):126–32. doi: 10.1038/s41586-021-03752-4 (PMC8338555; doi:10.1038/s41586-021-03752-4)

Caushi JX and Zhang J *et al*,

Distinct transcriptional programs characterize neoantigen-specific TIL in lung cancers treated with anti-PD-1

Supplementary Data File 5

Table of contents

Supplementary Data 5.1……………………….. Page 2

MANA-specific TCRs identified in MPR and non-MPR patients using the MANAFEST assays

Supplementary Data 5.2………….…………..… Page 9

Influenza-specific TCRs identified using the ViraFEST assay

Supplementary Data 5.3………………………. Page 12

The frequency of each MANA-specific TCR Vβ clonotype in each condition tested in the MANAFEST assay visualized by individual dot plots

Supplementary Data 5.1 MANA-specific TCRs identified in MPR and non-MPR patients using the MANAFEST assays. Antigen-specific responses identified using the MANAFEST assay are shown for MPRs MD01-005 (re-analyzed results are shown below; original results previously reported for this patient in Forde PM et al, N Engl J Med, 2018), MD043-008, MD043-003, and NY016-025. Antigen-specific responses identified using the MANAFEST assay are shown for non-MPRs MD043-011, NY016-007, and NY016-014. Each antigen-specific clonotypic expansion is color coded to indicate if the clone was not detected in the single cell data (blue), detected in the single cell data but not tested via TCR cloning (green), detected in the single cell data but did not validate with TCR cloning (orange), or detected in the single cell data and validated with TCR cloning (red). Data are shown as the percent of MANAFEST+ clonotypes among CD8+ T cells after 10 day culture. Neither MANA-specific nor CEF-specific T cell responses were detected in non-MPR patient, MD01-019. *Clones determined to recognize CEF were queried against a database of TCRs with known specificity (vdjdb, Supplementary Table 8). Several clonotypes were previously been reported to recognize common viral epitopes used in the CEF pool. Two of these clonotypes reported to recognize the influenza A matrix protein HLA A*02:01-restricted GILGFVFTL epitope. The full TCR corresponding to these clonotypes were cloned into the Jurkat transfer system and were validated to recognize the reported influenza antigen. Composite 3D bar plot for MD01-004 is shown in **Extended Data Fig. 2**.

Patient MD01-005

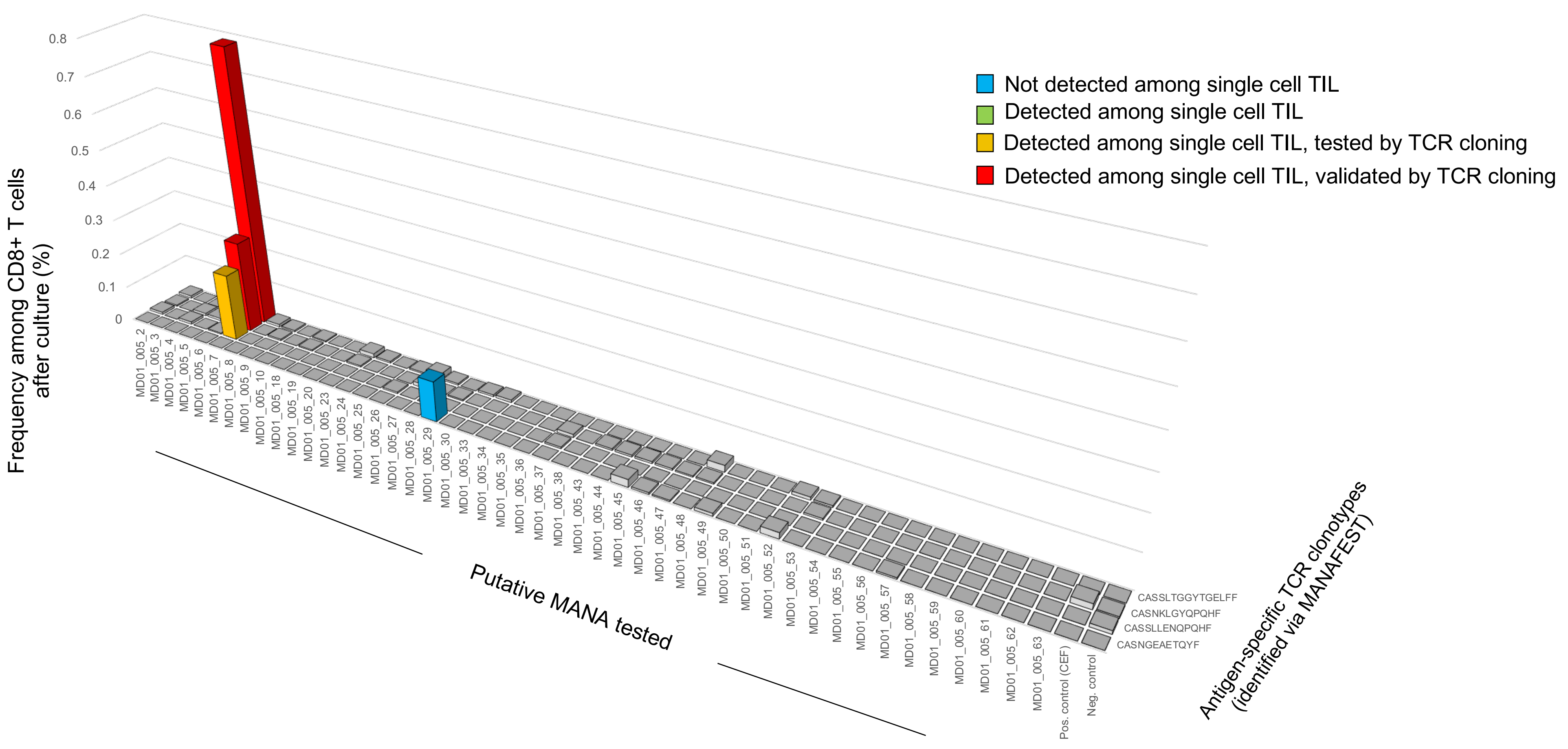

Patient MD043-008

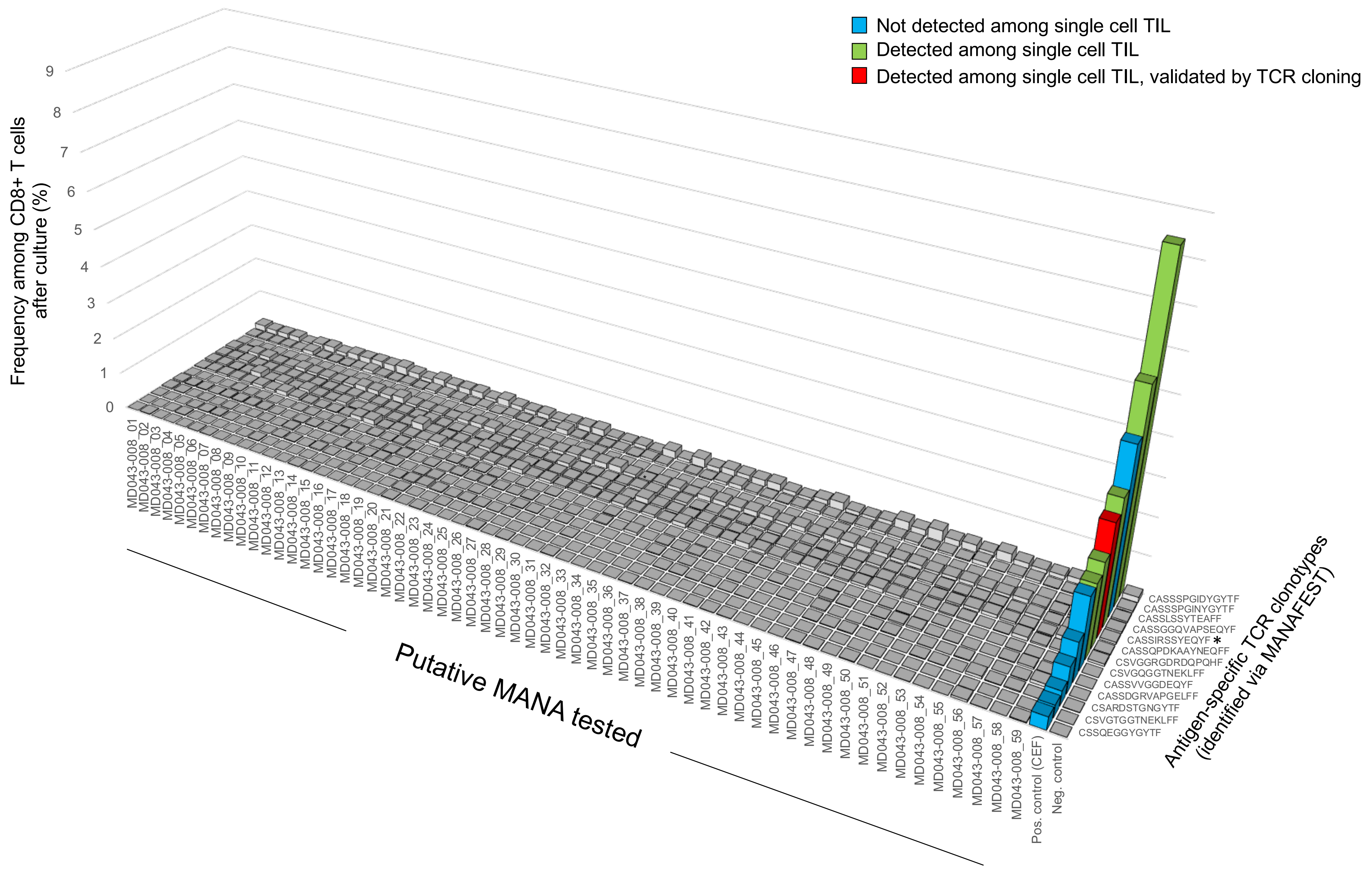

Patient MD043-003

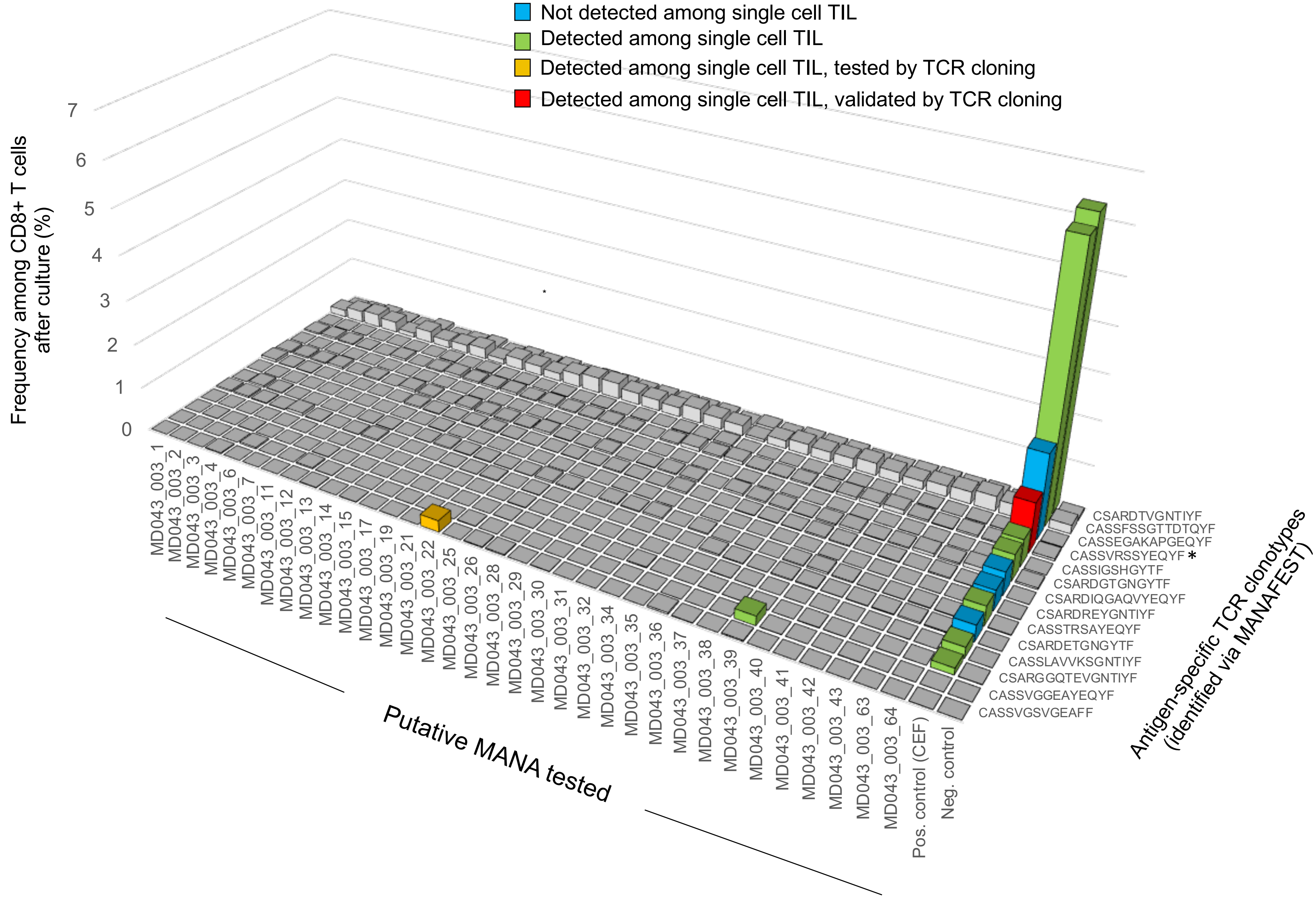

Patient NY016-025

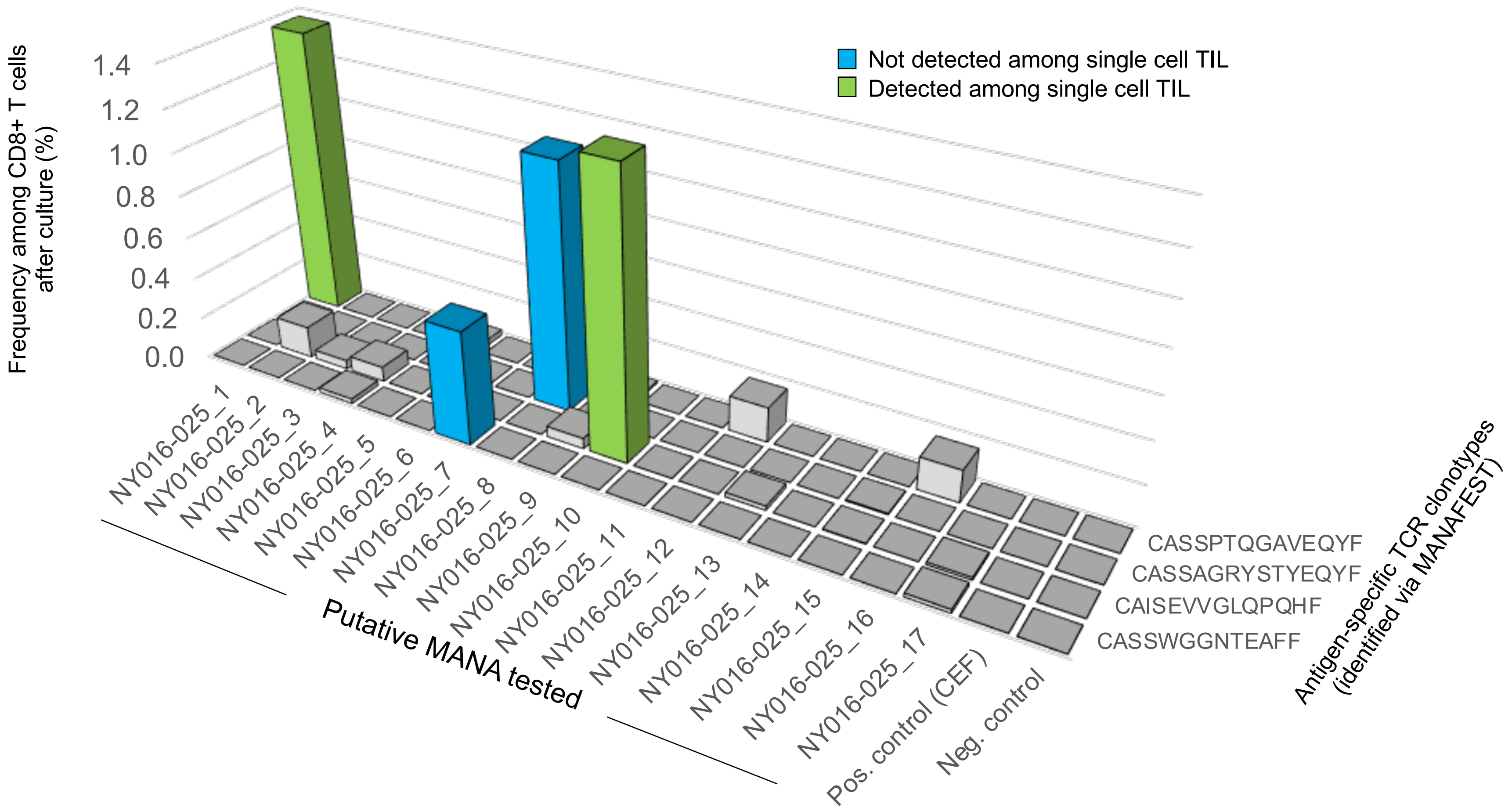

Patient MD043-011

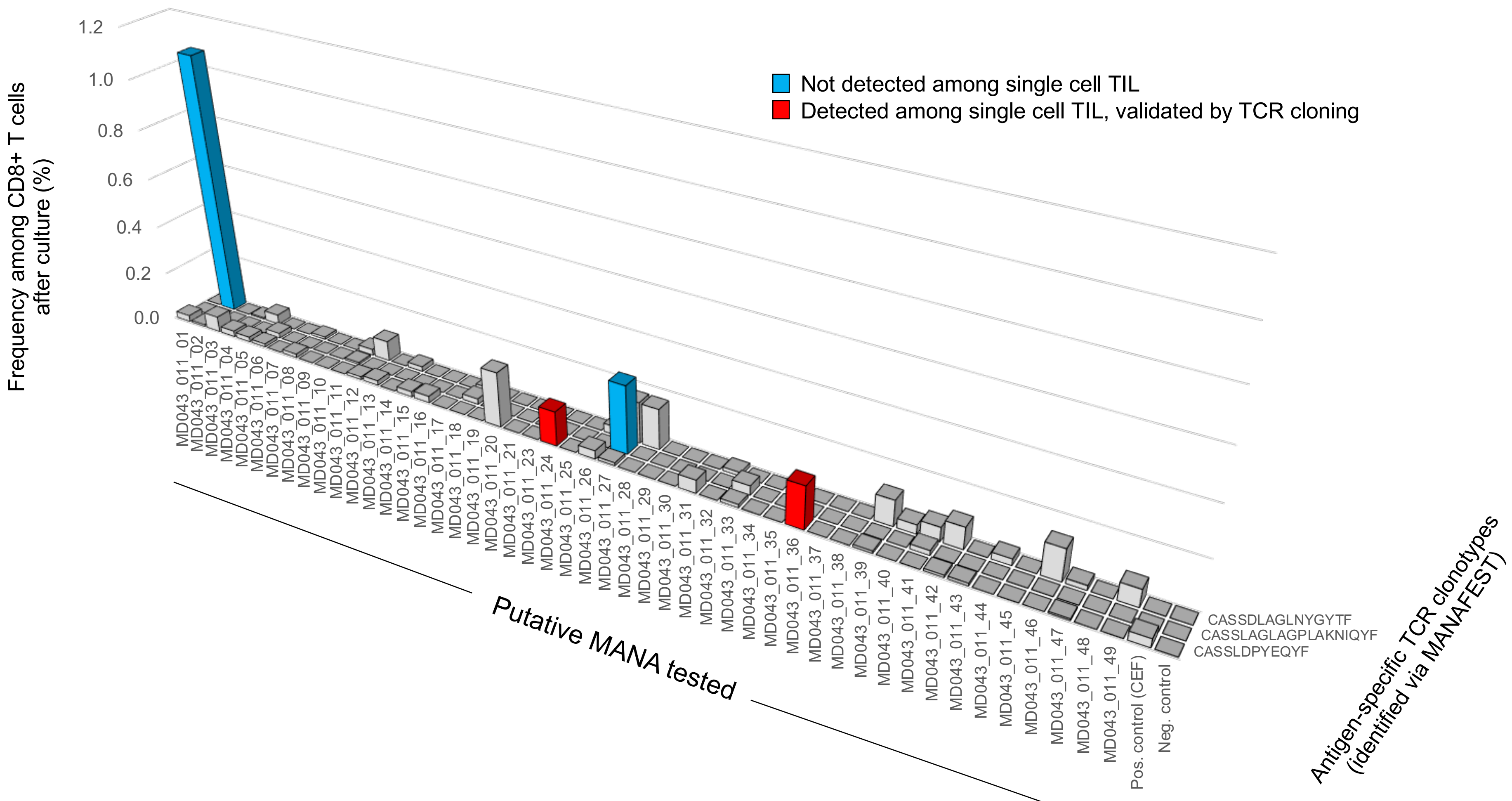

Patient NY016-007

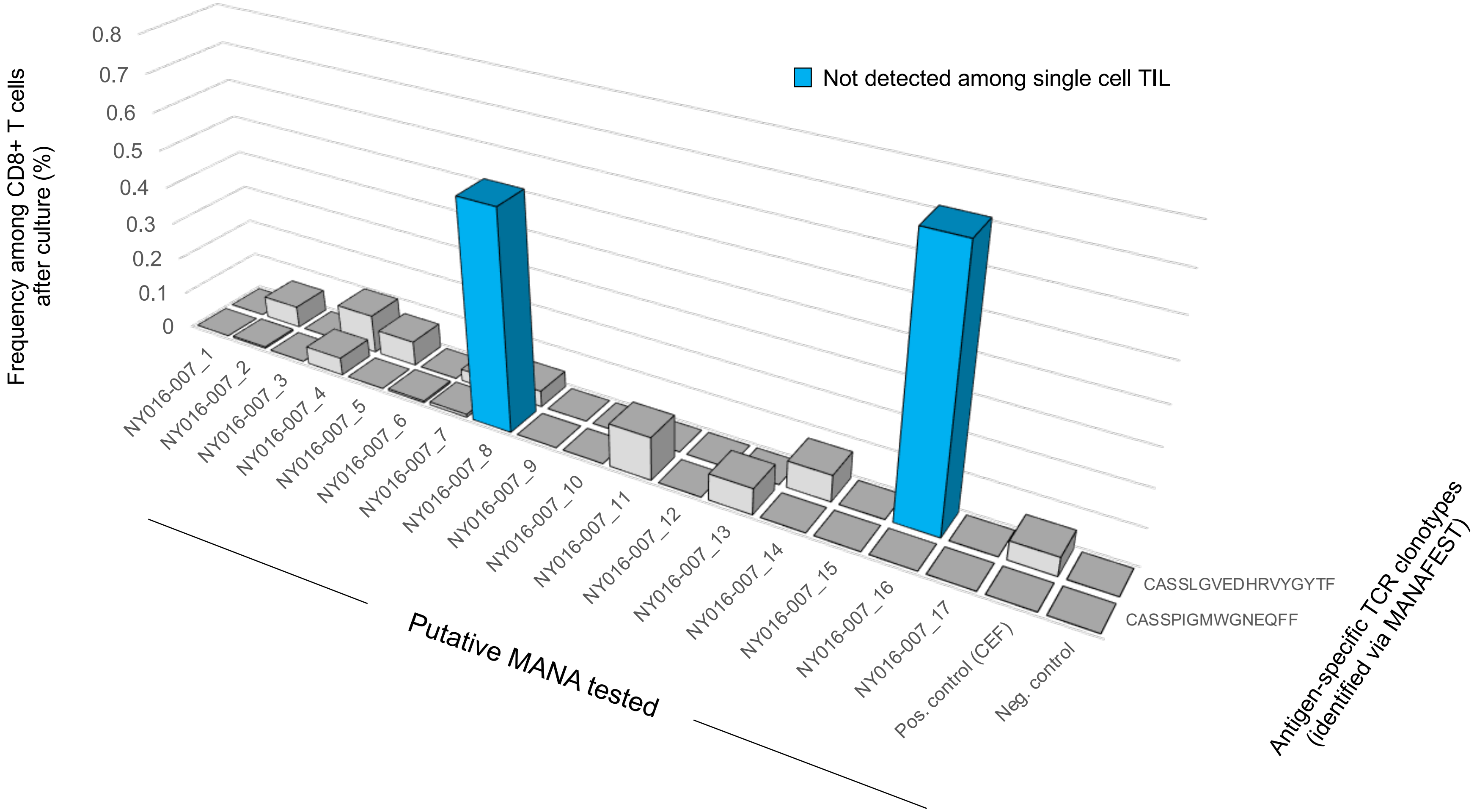

Patient NY016-014

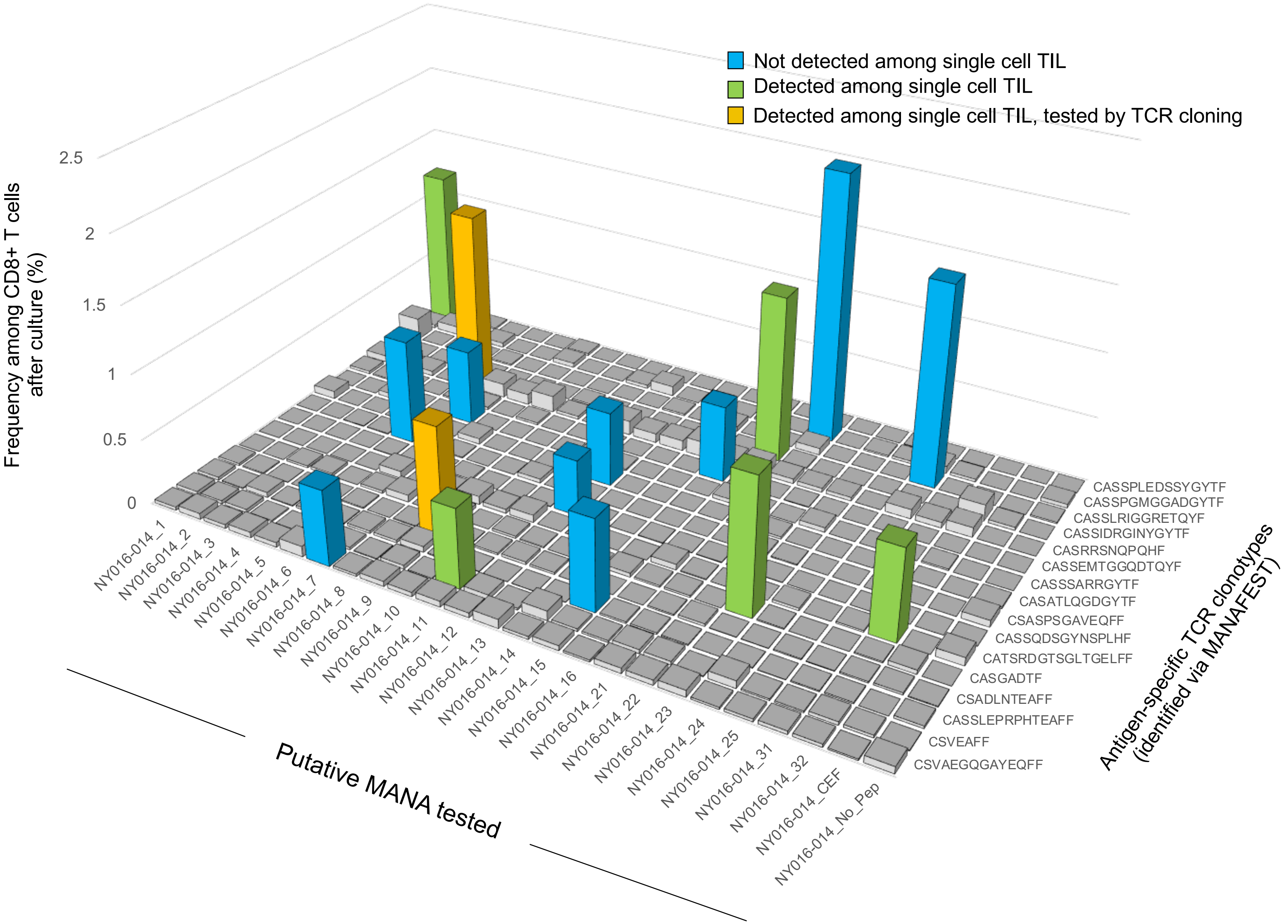

Supplementary Data 5.2. Influenza-specific TCRs identified using the ViraFEST assay. The ViraFEST assay was performed on non-MPR patients MD01-004 and MD043-011, and MPR patient MD01-005 to identify influenza-specific TCRβ clones. Influenza A pools consisting of overlapping peptides from the matrix and nucleocapsid proteins of H1N1 and H3N2, or an HIV-1 Gag pool as a negative control, were used to stimulate peripheral blood T cells in vitro for 10 days. Each flu peptide pool was tested in triplicate. Data are shown as the frequency of ViraFEST+ clonotypes among total CD8+ T cells for each culture condition. Clonotypes are color coded to indicate if they were not detected in single cell TIL (blue) or were detected in single cell TIL (green).

Patient MD01-004

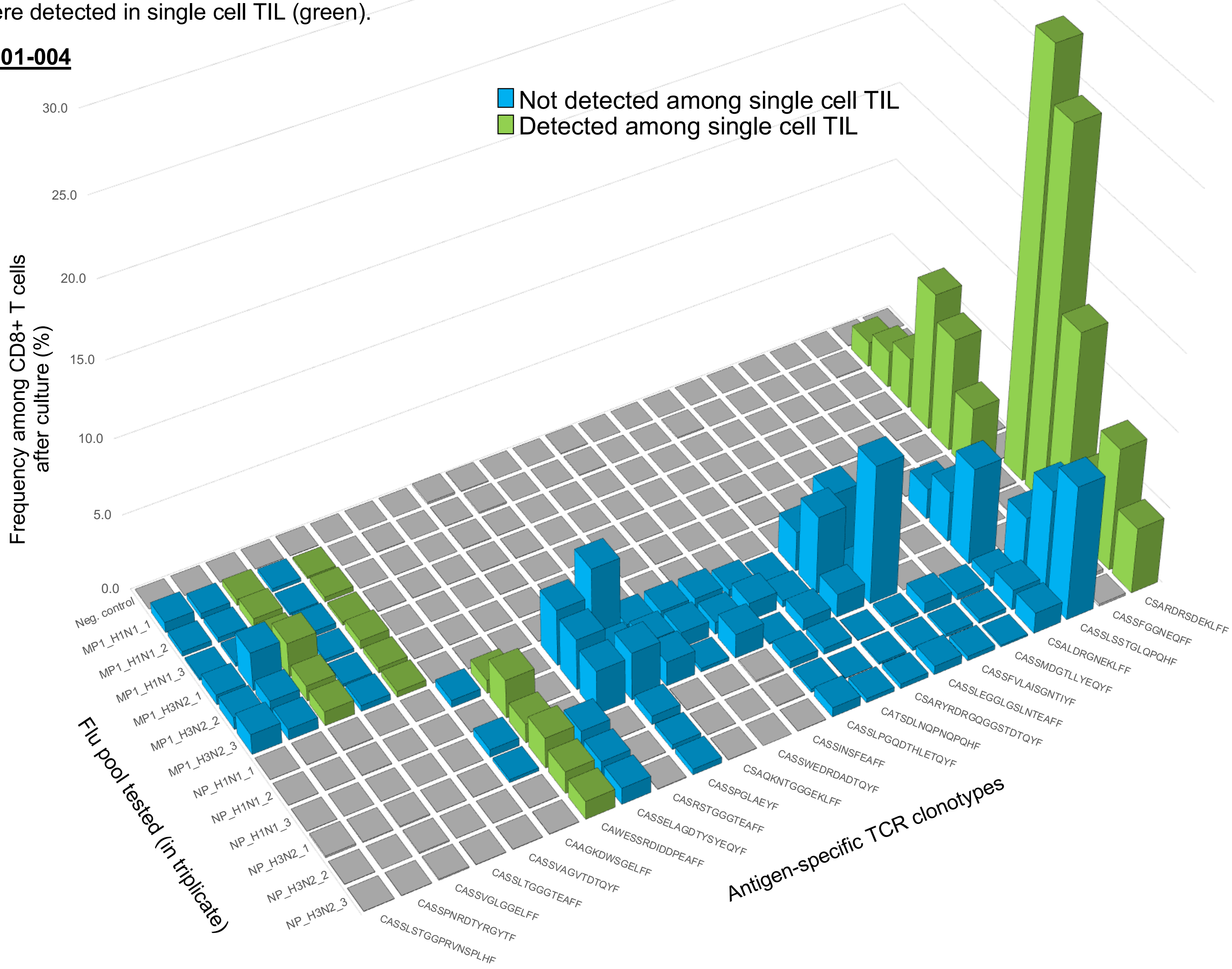

Patient MD043-011

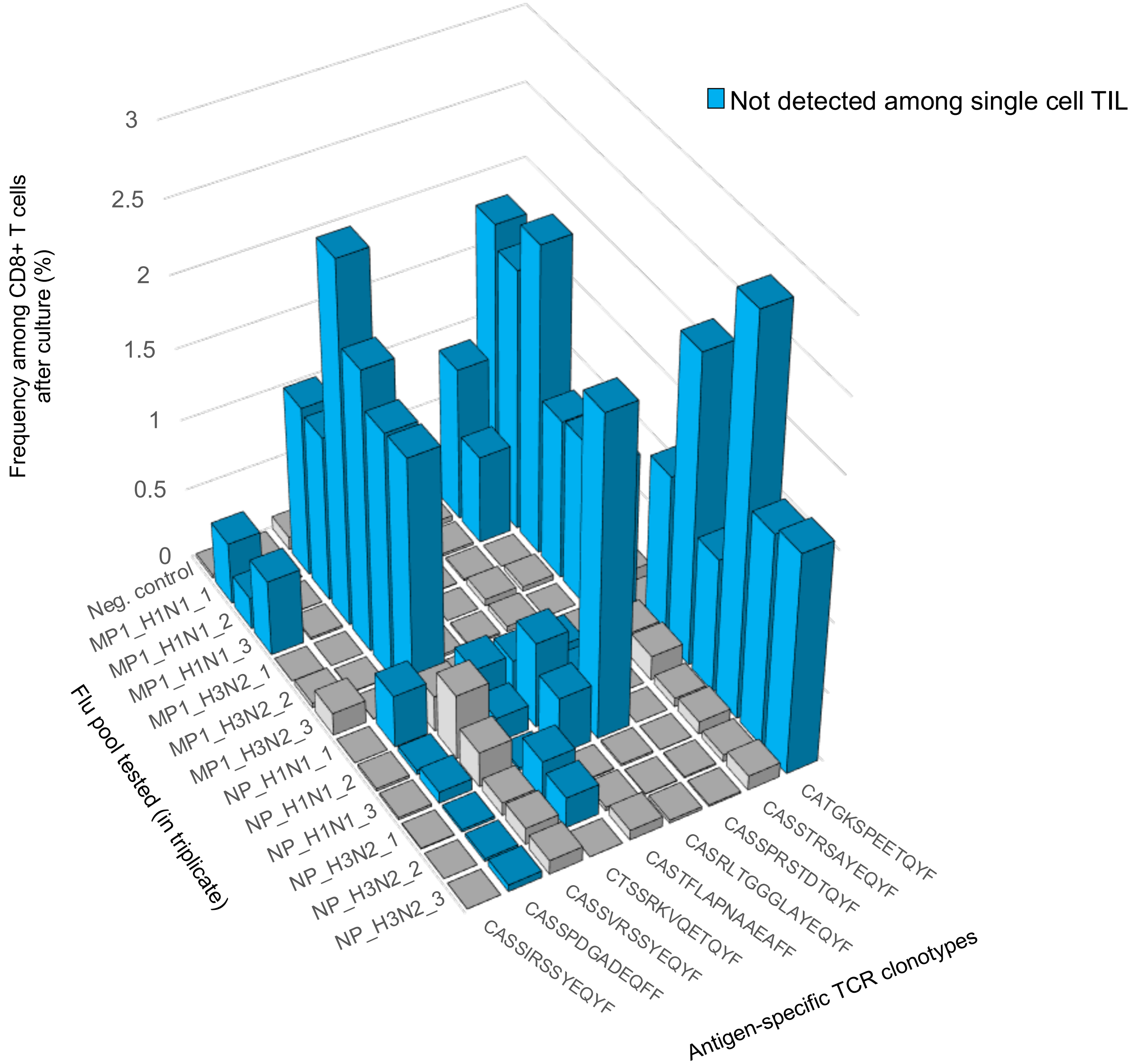

Patient MD01-005

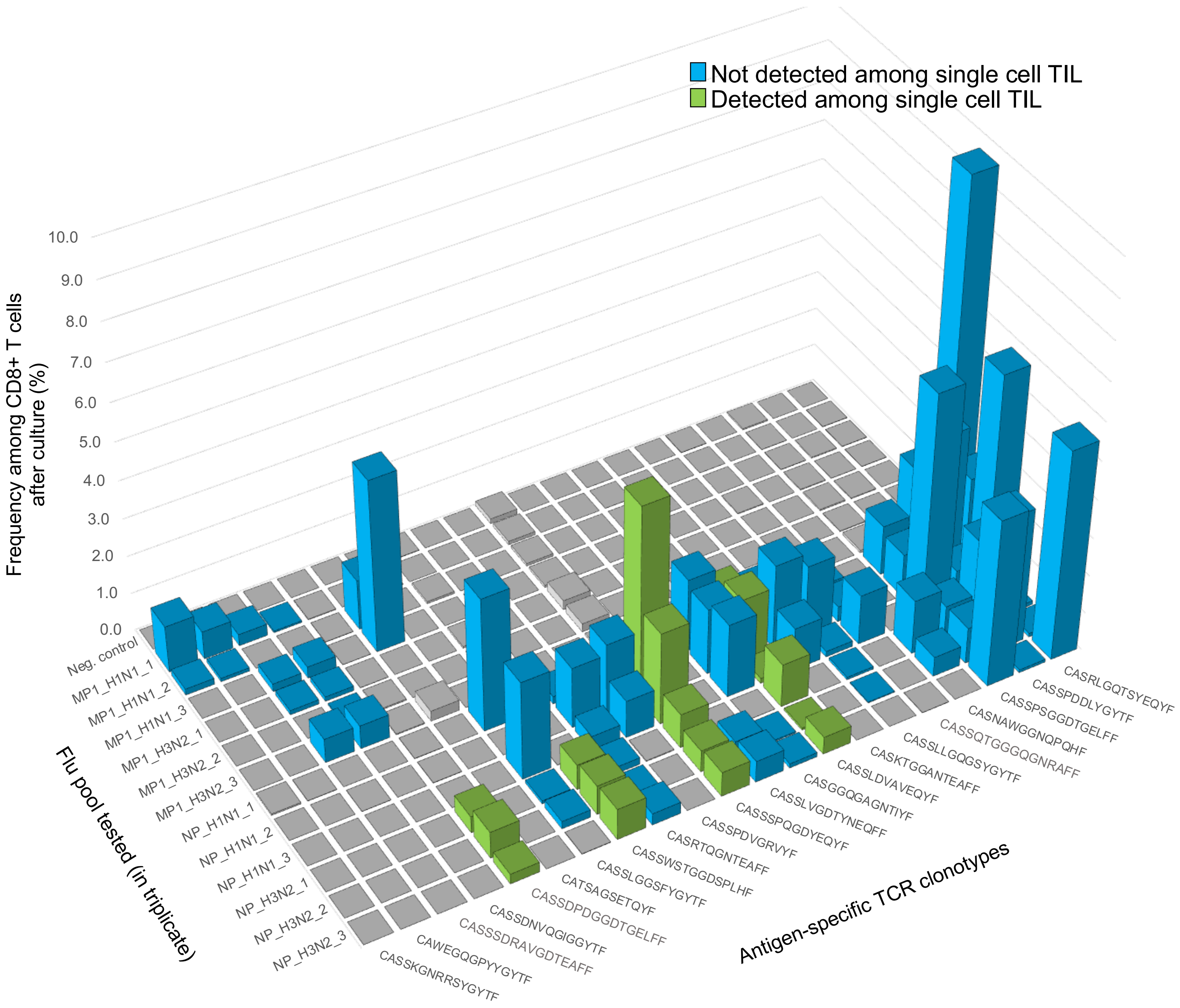

Supplementary Data 5.3 The frequency of each MANA-specific TCR Vβ clonotype in each condition tested in the MANAFEST assay visualized by individual dot plots. Peripheral blood T cells were tested for reactivity to putative MANA using the MANAFEST assay. A graph is shown for each MANA- and CEF-specific TCR Vβ clonotype (right y-axis) detected in each patient. The frequency of each clonotype is shown as the percent among all T cells detected by TCRseq after culture (left y-axis) in each peptide-stimulated condition, the CEF positive control well, and the “no peptide” negative control well (x-axis).

Patient MD01-004

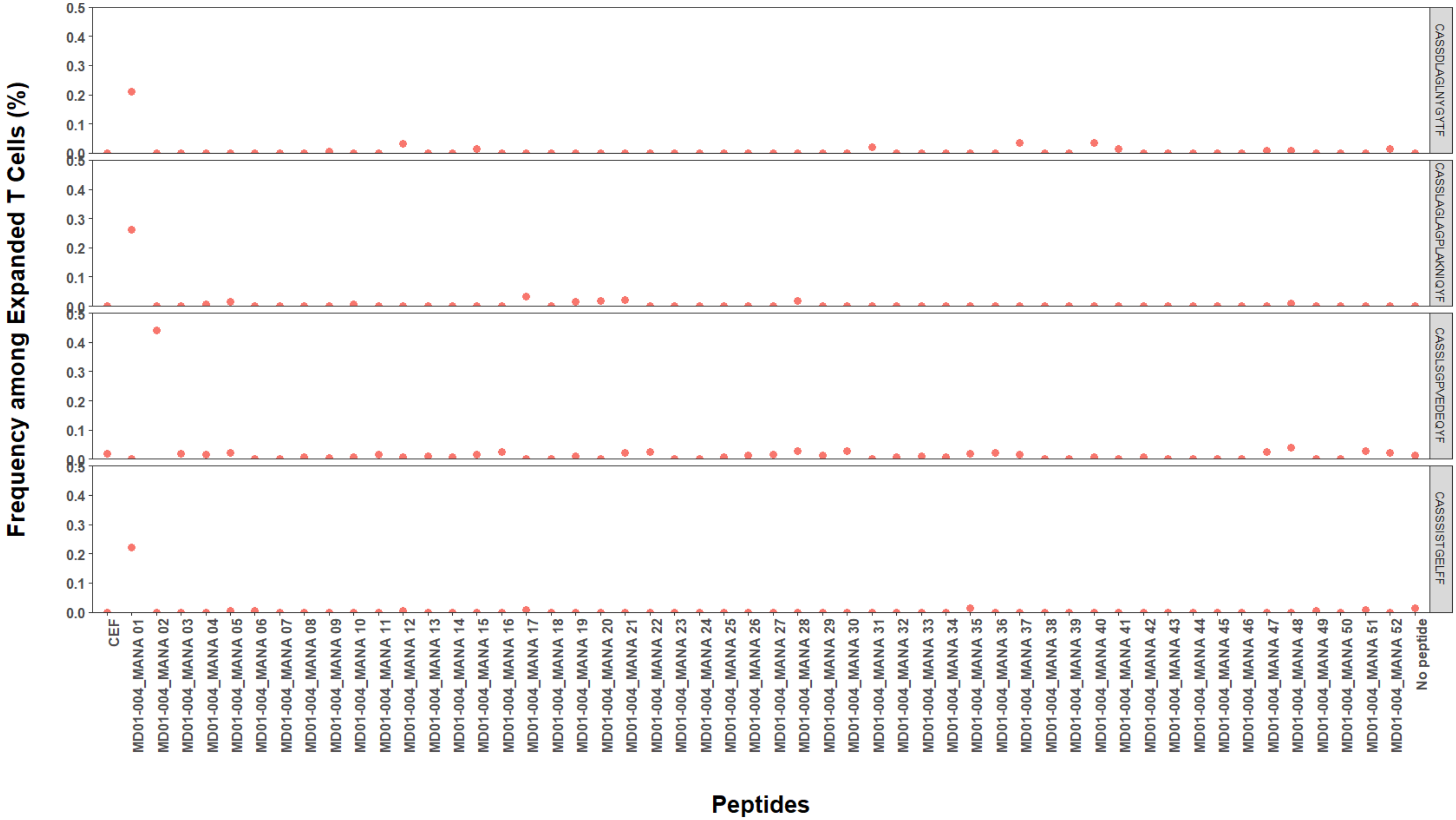

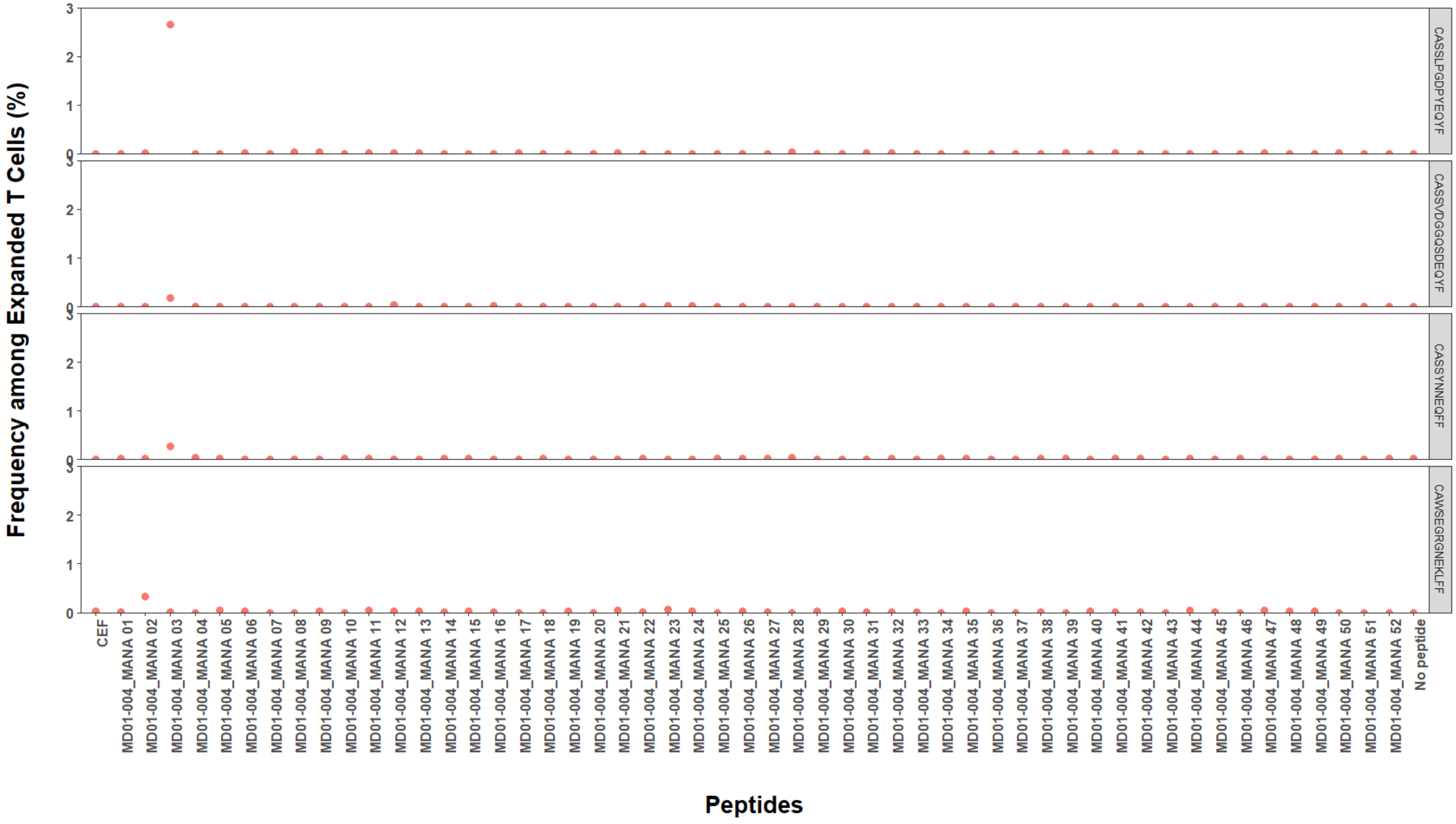
Frequency among Expanded T Cells (%)
3
2
1
0
CASSLPGDPYEQYF
CASSVDGGQSDEQYF
CASSYNNEQFF
CAWSEGRGNEKLFF
CEF
MD01-004_MANA 01
MD01-004_MANA 02
MD01-004_MANA 03
MD01-004_MANA 04
MD01-004_MANA 05
MD01-004_MANA 06
MD01-004_MANA 07
MD01-004_MANA 08
MD01-004_MANA 09
MD01-004_MANA 10
MD01-004_MANA 11
MD01-004_MANA 12
MD01-004_MANA 13
MD01-004_MANA 14
MD01-004_MANA 15
MD01-004_MANA 16
MD01-004_MANA 17
MD01-004_MANA 18
MD01-004_MANA 19
MD01-004_MANA 20
MD01-004_MANA 21
MD01-004_MANA 22
MD01-004_MANA 23
MD01-004_MANA 24
MD01-004_MANA 25
MD01-004_MANA 26
MD01-004_MANA 27
MD01-004_MANA 28
MD01-004_MANA 29
MD01-004_MANA 30
MD01-004_MANA 31
MD01-004_MANA 32
MD01-004_MANA 33
MD01-004_MANA 34
MD01-004_MANA 35
MD01-004_MANA 36
MD01-004_MANA 37
MD01-004_MANA 38
MD01-004_MANA 39
MD01-004_MANA 40
MD01-004_MANA 41
MD01-004_MANA 42
MD01-004_MANA 43
MD01-004_MANA 44
MD01-004_MANA 45
MD01-004_MANA 46
MD01-004_MANA 47
MD01-004_MANA 48
MD01-004_MANA 49
MD01-004_MANA 50
MD01-004_MANA 51
MD01-004_MANA 52
No peptide
Peptides

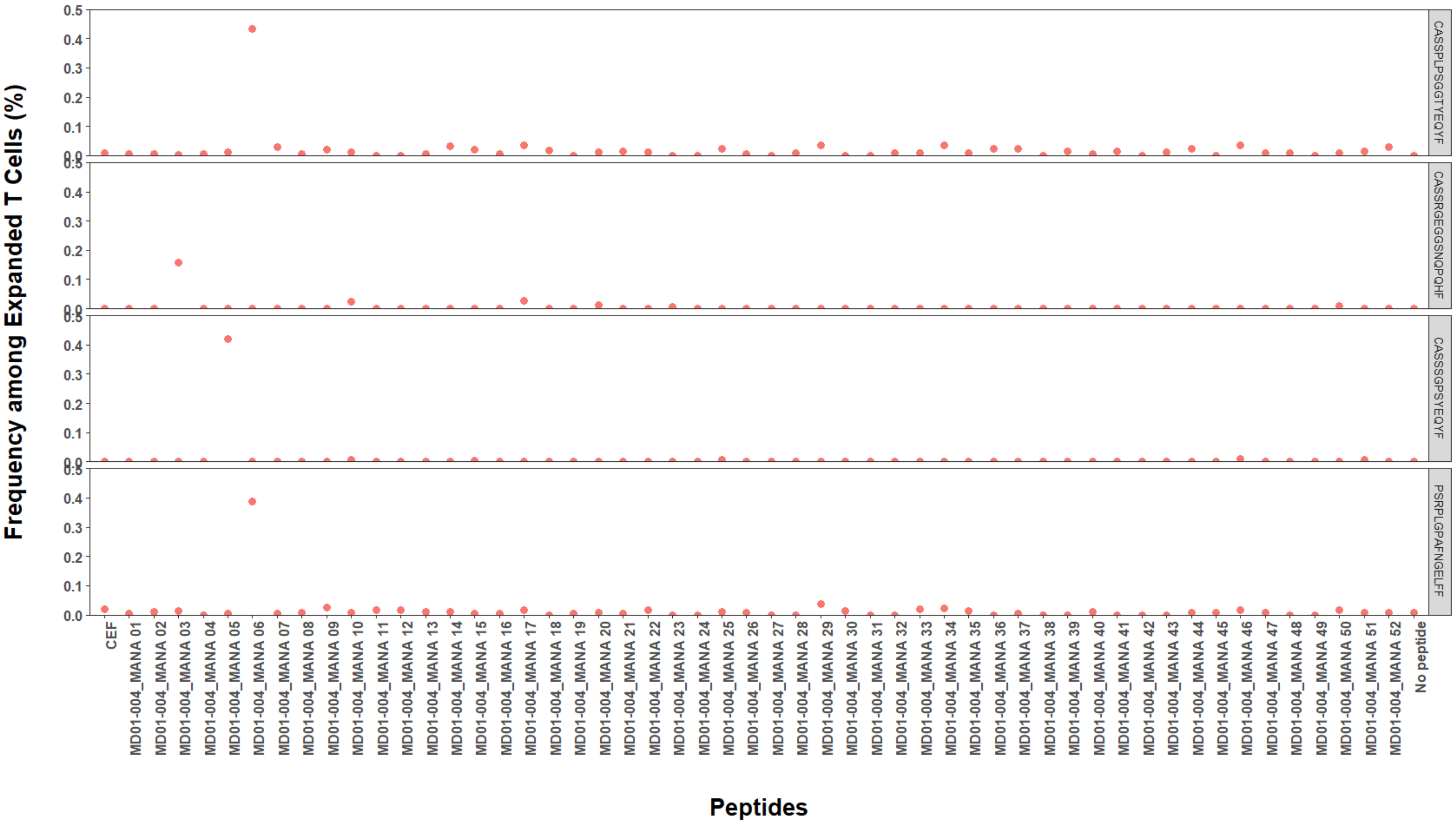

Frequency among Expanded T Cells (%)
CASSPLPSGGTYEQYF
CASSRGEGGSNQPQHF
CASSSGPSYEQYF
PSRPLGPAFNGELFF
Peptides

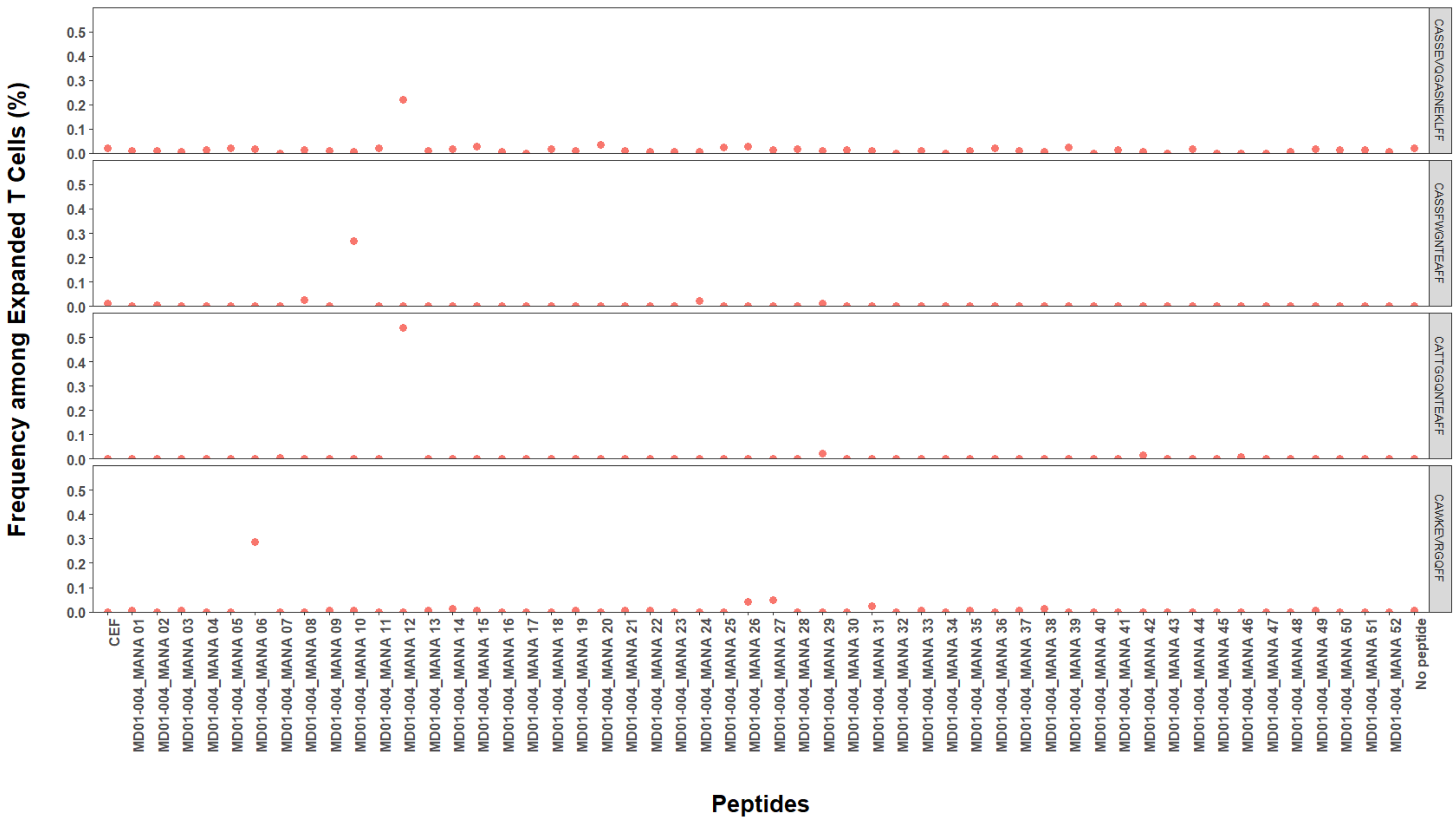

Frequency among Expanded T Cells (%)
0.0
0.1
0.2
0.3
0.4
0.5
CASSEVQGASNEKLFF
CASSFWGNTEAFF
CATTGGQNTEAFF
CAWKEVRGQFF
CEF
MD01-004_MANA 01
MD01-004_MANA 02
MD01-004_MANA 03
MD01-004_MANA 04
MD01-004_MANA 05
MD01-004_MANA 06
MD01-004_MANA 07
MD01-004_MANA 08
MD01-004_MANA 09
MD01-004_MANA 10
MD01-004_MANA 11
MD01-004_MANA 12
MD01-004_MANA 13
MD01-004_MANA 14
MD01-004_MANA 15
MD01-004_MANA 16
MD01-004_MANA 17
MD01-004_MANA 18
MD01-004_MANA 19
MD01-004_MANA 20
MD01-004_MANA 21
MD01-004_MANA 22
MD01-004_MANA 23
MD01-004_MANA 24
MD01-004_MANA 25
MD01-004_MANA 26
MD01-004_MANA 27
MD01-004_MANA 28
MD01-004_MANA 29
MD01-004_MANA 30
MD01-004_MANA 31
MD01-004_MANA 32
MD01-004_MANA 33
MD01-004_MANA 34
MD01-004_MANA 35
MD01-004_MANA 36
MD01-004_MANA 37
MD01-004_MANA 38
MD01-004_MANA 39
MD01-004_MANA 40
MD01-004_MANA 41
MD01-004_MANA 42
MD01-004_MANA 43
MD01-004_MANA 44
MD01-004_MANA 45
MD01-004_MANA 46
MD01-004_MANA 47
MD01-004_MANA 48
MD01-004_MANA 49
MD01-004_MANA 50
MD01-004_MANA 51
MD01-004_MANA 52
No peptide
Peptides

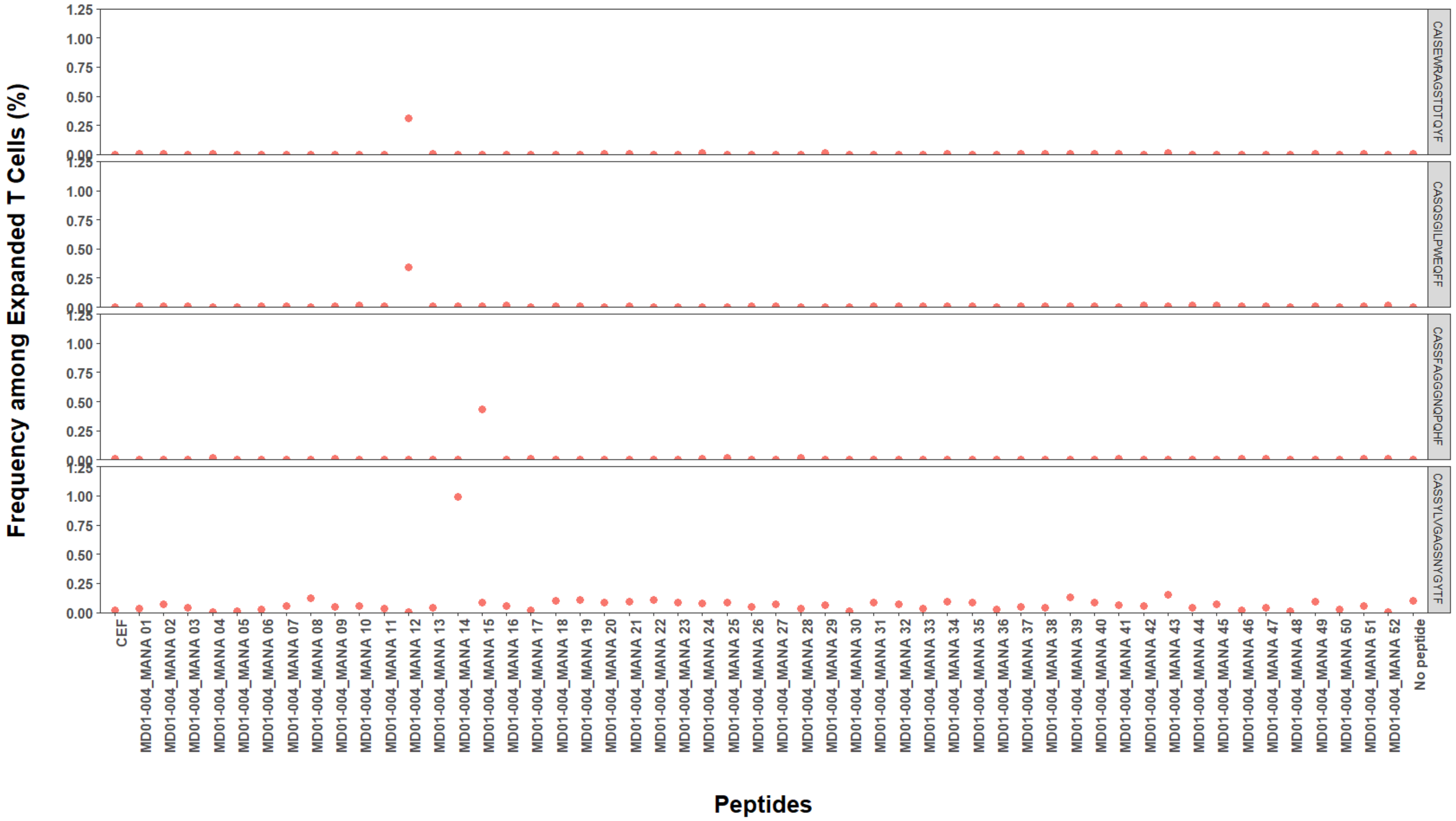

Frequency among Expanded T Cells (%)
Peptides
CAISEWRAGSTDTQYF
CASQSGILPWEQFF
CASSFAGGGNQPQHF
CASSYLVGAGSNYGYTF
0.00
0.25
0.50
0.75
1.00
1.25
CEF
MD01-004_MANA 01
MD01-004_MANA 52
No peptide

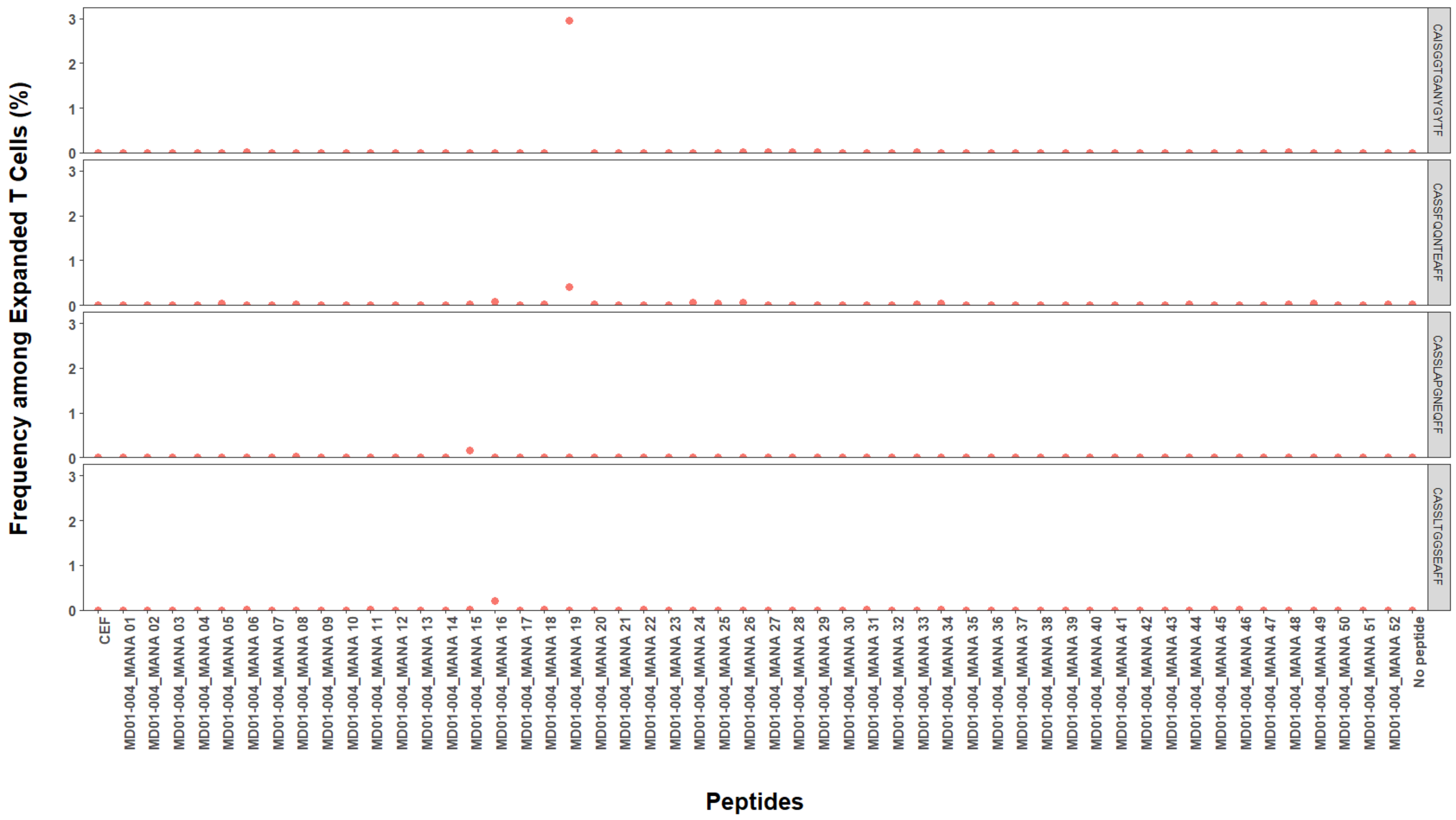
Frequency among Expanded T Cells (%)
Peptides
CAISGGTGANYGYTF
CASSFQNTEAFF
CASSLAPGNEQFF
CASSLTGGSEAFF
0
1
2
3
CEF
MD01-004_MANA 01
MD01-004_MANA 02
MD01-004_MANA 03
MD01-004_MANA 04
MD01-004_MANA 05
MD01-004_MANA 06
MD01-004_MANA 07
MD01-004_MANA 08
MD01-004_MANA 09
MD01-004_MANA 10
MD01-004_MANA 11
MD01-004_MANA 12
MD01-004_MANA 13
MD01-004_MANA 14
MD01-004_MANA 15
MD01-004_MANA 16
MD01-004_MANA 17
MD01-004_MANA 18
MD01-004_MANA 19
MD01-004_MANA 20
MD01-004_MANA 21
MD01-004_MANA 22
MD01-004_MANA 23
MD01-004_MANA 24
MD01-004_MANA 25
MD01-004_MANA 26
MD01-004_MANA 27
MD01-004_MANA 28
MD01-004_MANA 29
MD01-004_MANA 30
MD01-004_MANA 31
MD01-004_MANA 32
MD01-004_MANA 33
MD01-004_MANA 34
MD01-004_MANA 35
MD01-004_MANA 36
MD01-004_MANA 37
MD01-004_MANA 38
MD01-004_MANA 39
MD01-004_MANA 40
MD01-004_MANA 41
MD01-004_MANA 42
MD01-004_MANA 43
MD01-004_MANA 44
MD01-004_MANA 45
MD01-004_MANA 46
MD01-004_MANA 47
MD01-004_MANA 48
MD01-004_MANA 49
MD01-004_MANA 50
MD01-004_MANA 51
MD01-004_MANA 52
No peptide

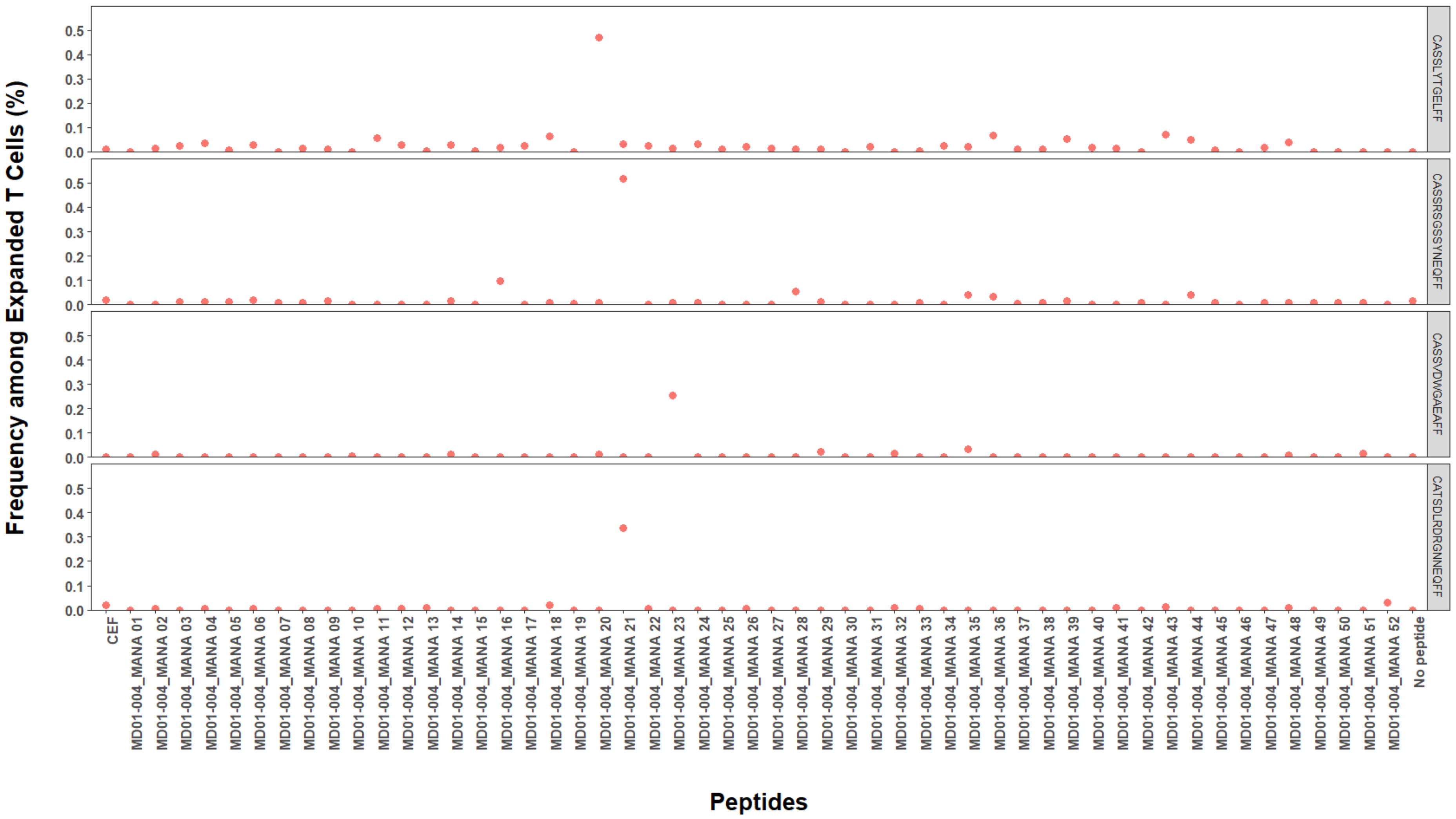

Frequency among Expanded T Cells (%)
CASSLYTGELFF
CASSRSGSSYNEQFF
CASSVDWGAEAFF
CATSDLRDRGNNEQFF
0.0
0.1
0.2
0.3
0.4
0.5
CEF
MD01-004_MANA 01
MD01-004_MANA 02
MD01-004_MANA 03
MD01-004_MANA 04
MD01-004_MANA 05
MD01-004_MANA 06
MD01-004_MANA 07
MD01-004_MANA 08
MD01-004_MANA 09
MD01-004_MANA 10
MD01-004_MANA 11
MD01-004_MANA 12
MD01-004_MANA 13
MD01-004_MANA 14
MD01-004_MANA 15
MD01-004_MANA 16
MD01-004_MANA 17
MD01-004_MANA 18
MD01-004_MANA 19
MD01-004_MANA 20
MD01-004_MANA 21
MD01-004_MANA 22
MD01-004_MANA 23
MD01-004_MANA 24
MD01-004_MANA 25
MD01-004_MANA 26
MD01-004_MANA 27
MD01-004_MANA 28
MD01-004_MANA 29
MD01-004_MANA 30
MD01-004_MANA 31
MD01-004_MANA 32
MD01-004_MANA 33
MD01-004_MANA 34
MD01-004_MANA 35
MD01-004_MANA 36
MD01-004_MANA 37
MD01-004_MANA 38
MD01-004_MANA 39
MD01-004_MANA 40
MD01-004_MANA 41
MD01-004_MANA 42
MD01-004_MANA 43
MD01-004_MANA 44
MD01-004_MANA 45
MD01-004_MANA 46
MD01-004_MANA 47
MD01-004_MANA 48
MD01-004_MANA 49
MD01-004_MANA 50
MD01-004_MANA 51
MD01-004_MANA 52
No peptide
Peptides

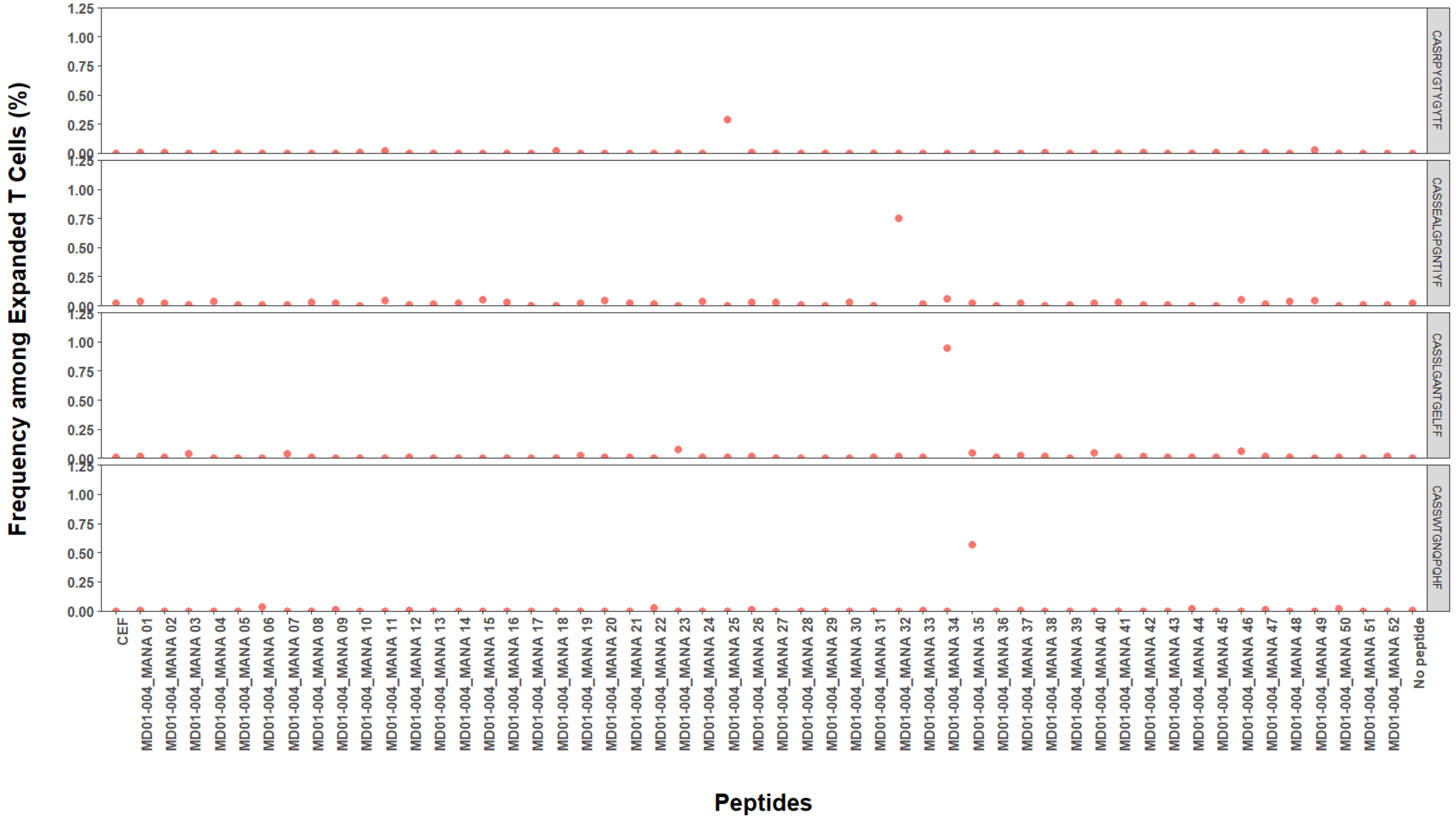
Frequency among Expanded T Cells (%)
Peptides
CASRPYGTYGYTF
CASSEALGPGNTIYF
CASSLGANTGELFF
CASSWTGNQPQHF
0.00
0.25
0.50
0.75
1.00
1.25
CEF
MD01-004_MANA 01
MD01-004_MANA 02
MD01-004_MANA 03
MD01-004_MANA 04
MD01-004_MANA 05
MD01-004_MANA 06
MD01-004_MANA 07
MD01-004_MANA 08
MD01-004_MANA 09
MD01-004_MANA 10
MD01-004_MANA 11
MD01-004_MANA 12
MD01-004_MANA 13
MD01-004_MANA 14
MD01-004_MANA 15
MD01-004_MANA 16
MD01-004_MANA 17
MD01-004_MANA 18
MD01-004_MANA 19
MD01-004_MANA 20
MD01-004_MANA 21
MD01-004_MANA 22
MD01-004_MANA 23
MD01-004_MANA 24
MD01-004_MANA 25
MD01-004_MANA 26
MD01-004_MANA 27
MD01-004_MANA 28
MD01-004_MANA 29
MD01-004_MANA 30
MD01-004_MANA 31
MD01-004_MANA 32
MD01-004_MANA 33
MD01-004_MANA 34
MD01-004_MANA 35
MD01-004_MANA 36
MD01-004_MANA 37
MD01-004_MANA 38
MD01-004_MANA 39
MD01-004_MANA 40
MD01-004_MANA 41
MD01-004_MANA 42
MD01-004_MANA 43
MD01-004_MANA 44
MD01-004_MANA 45
MD01-004_MANA 46
MD01-004_MANA 47
MD01-004_MANA 48
MD01-004_MANA 49
MD01-004_MANA 50
MD01-004_MANA 51
MD01-004_MANA 52
No peptide

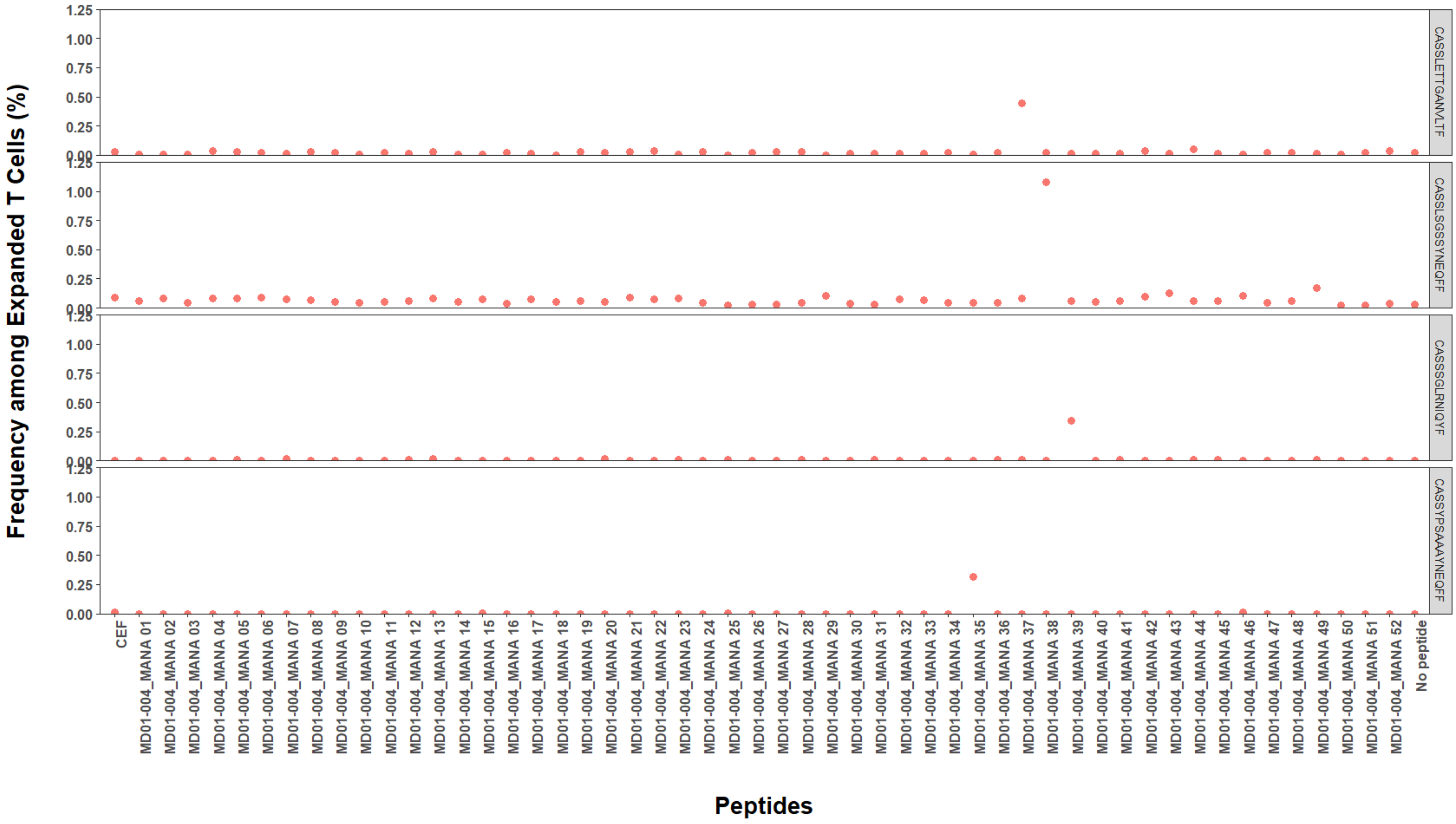

Frequency among Expanded T Cells (%)
1.25
1.00
0.75
0.50
0.25
0.00
CASSLETTGANVLTF
CASSLSGSSYNEQFF
CASSSGLRNIQYF
CASSYPSAAYNEQFF
CEF
MD01-004_MANA 01
MD01-004_MANA 02
MD01-004_MANA 03
MD01-004_MANA 04
MD01-004_MANA 05
MD01-004_MANA 06
MD01-004_MANA 07
MD01-004_MANA 08
MD01-004_MANA 09
MD01-004_MANA 10
MD01-004_MANA 11
MD01-004_MANA 12
MD01-004_MANA 13
MD01-004_MANA 14
MD01-004_MANA 15
MD01-004_MANA 16
MD01-004_MANA 17
MD01-004_MANA 18
MD01-004_MANA 19
MD01-004_MANA 20
MD01-004_MANA 21
MD01-004_MANA 22
MD01-004_MANA 23
MD01-004_MANA 24
MD01-004_MANA 25
MD01-004_MANA 26
MD01-004_MANA 27
MD01-004_MANA 28
MD01-004_MANA 29
MD01-004_MANA 30
MD01-004_MANA 31
MD01-004_MANA 32
MD01-004_MANA 33
MD01-004_MANA 34
MD01-004_MANA 35
MD01-004_MANA 36
MD01-004_MANA 37
MD01-004_MANA 38
MD01-004_MANA 39
MD01-004_MANA 40
MD01-004_MANA 41
MD01-004_MANA 42
MD01-004_MANA 43
MD01-004_MANA 44
MD01-004_MANA 45
MD01-004_MANA 46
MD01-004_MANA 47
MD01-004_MANA 48
MD01-004_MANA 49
MD01-004_MANA 50
MD01-004_MANA 51
MD01-004_MANA 52
No peptide
Peptides

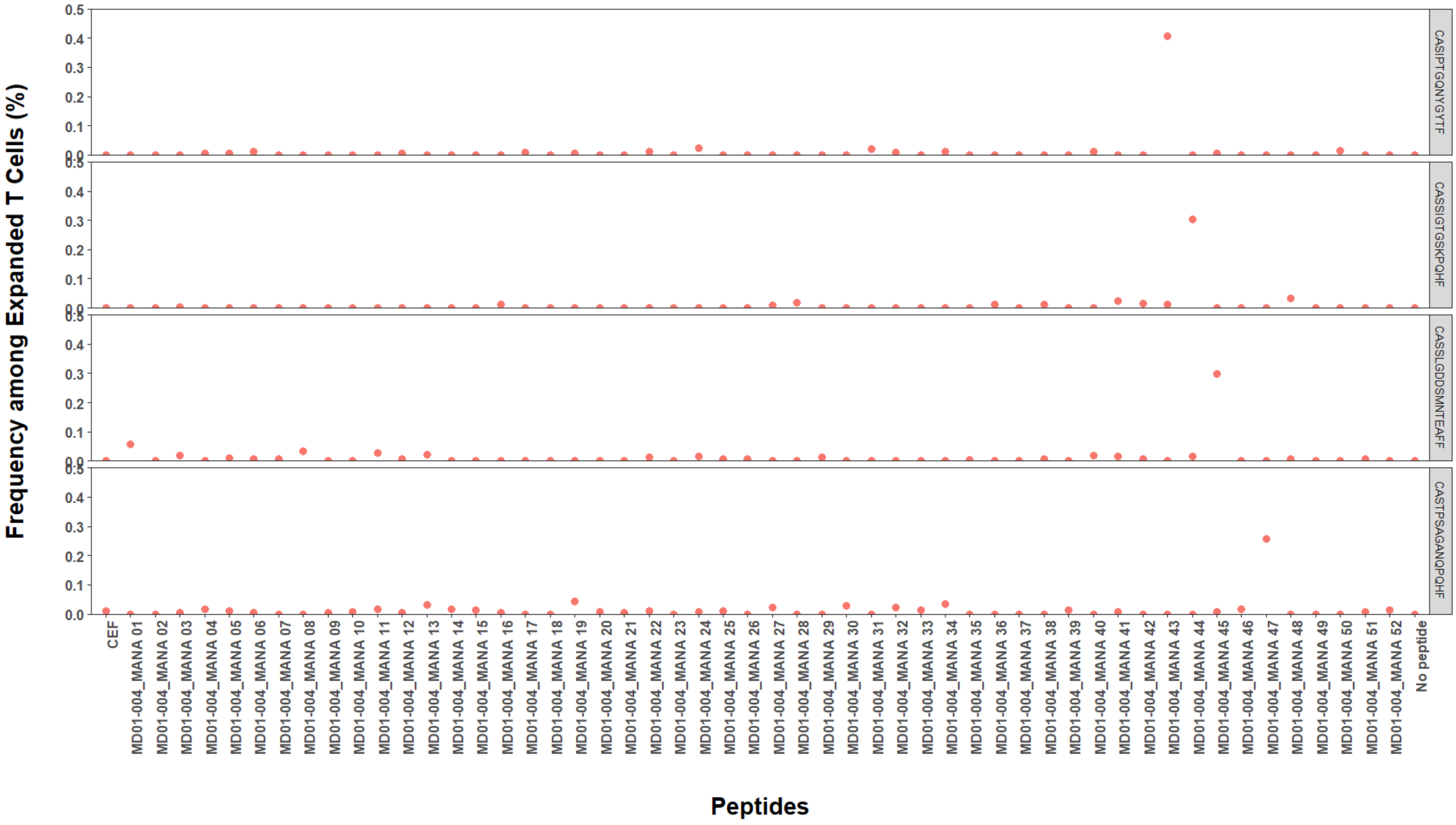
Frequency among Expanded T Cells (%)
Peptides
CASIPTGQNYGYTF
CASSIGTGSKPQHF
CASSLGDDSMNTEAFF
CASTPSAGANQPQHF
0.0
0.1
0.2
0.3
0.4
0.5
CEF
MD01-004_MANA 01
MD01-004_MANA 52
No peptide

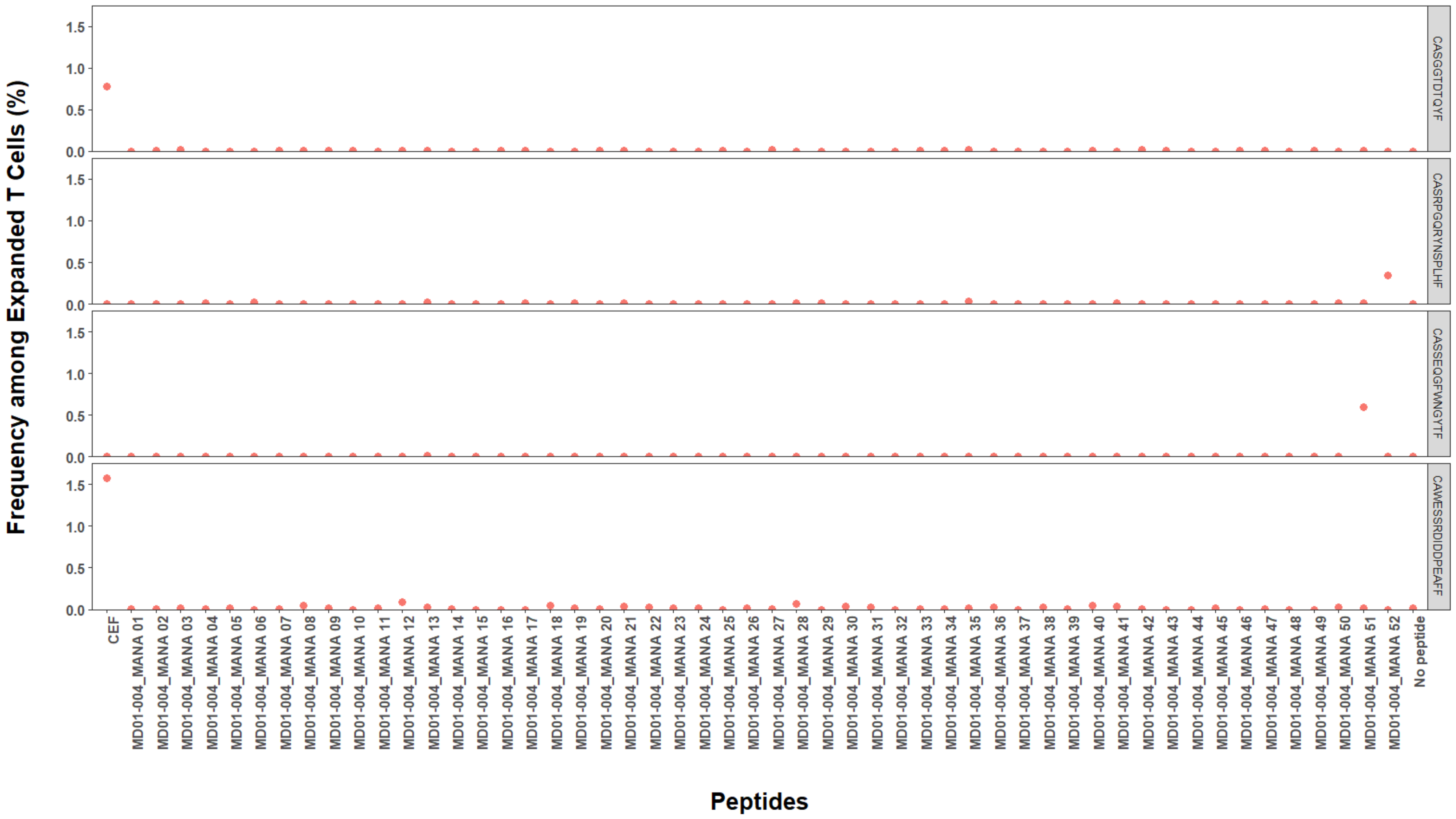
Frequency among Expanded T Cells (%)
Peptides
CASGGTDTQYF
CASRPGQRYNSPLHF
CASSEQGFWNGYTF
CAWESSRDIDDPEAFF
1.5
1.0
0.5
0.0
CEF
MD01-004_MANA 01
MD01-004_MANA 52
No peptide

Patient MD01-005

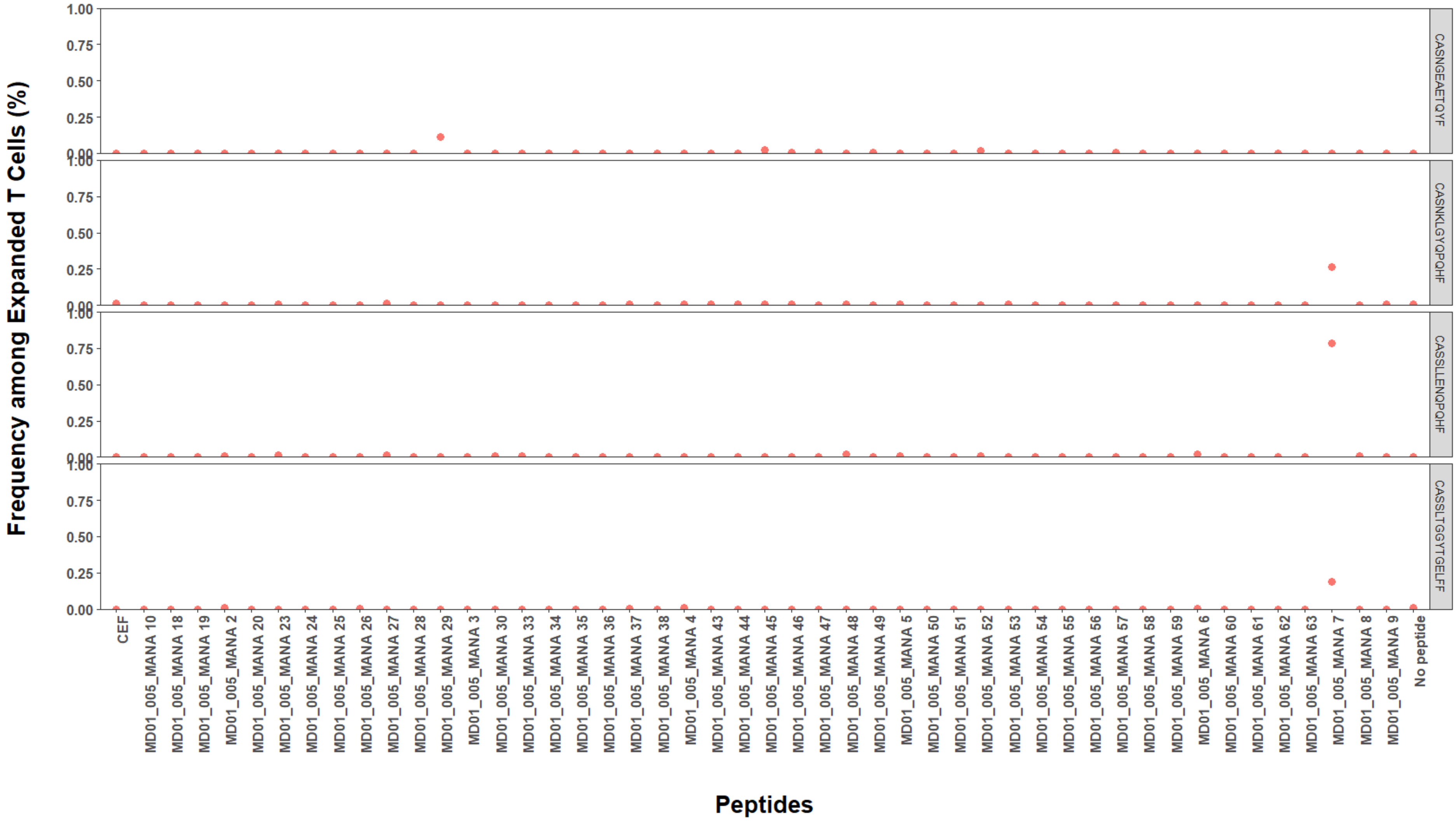

Patient MD043-011

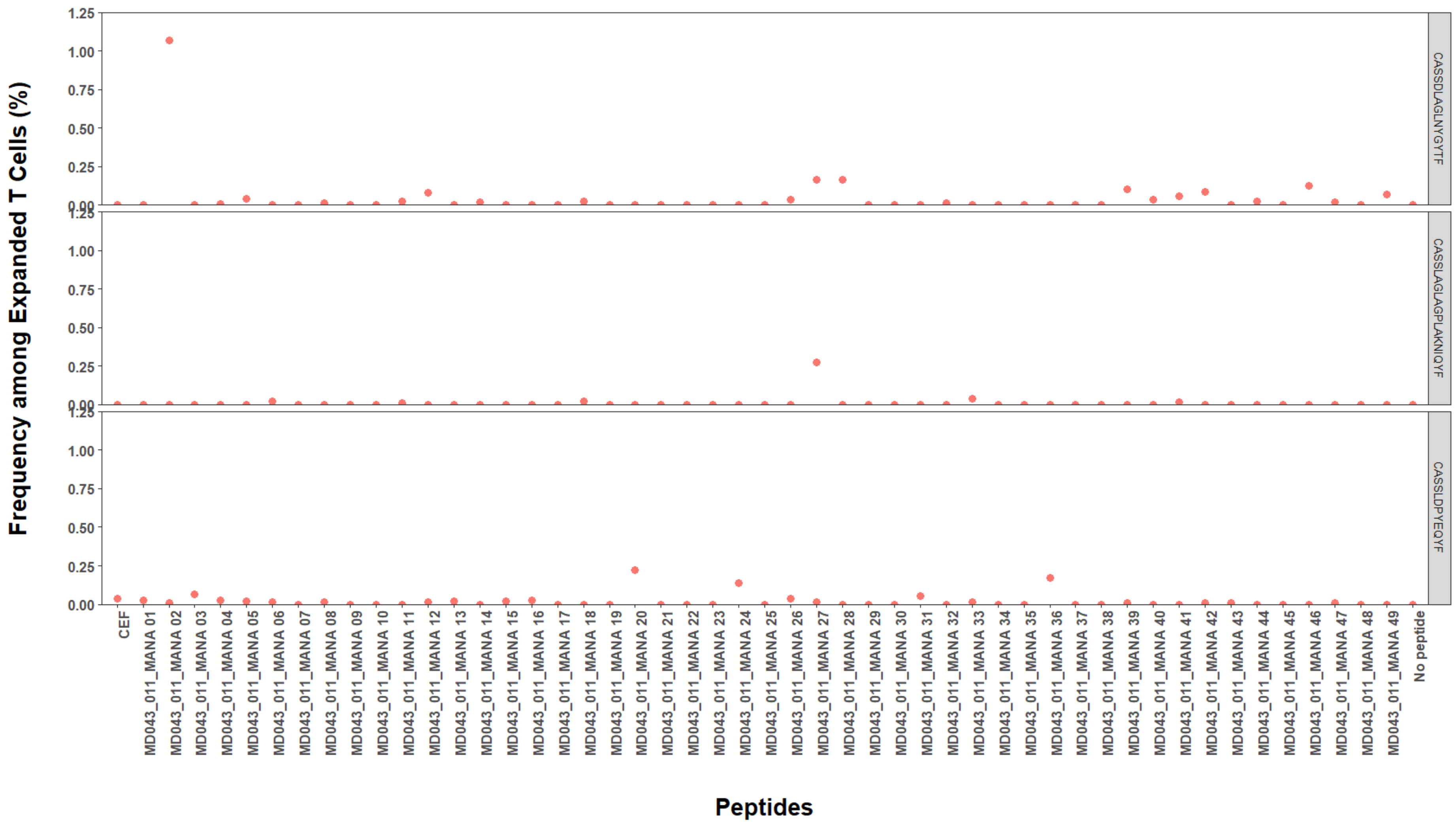

Patient: MD043-003

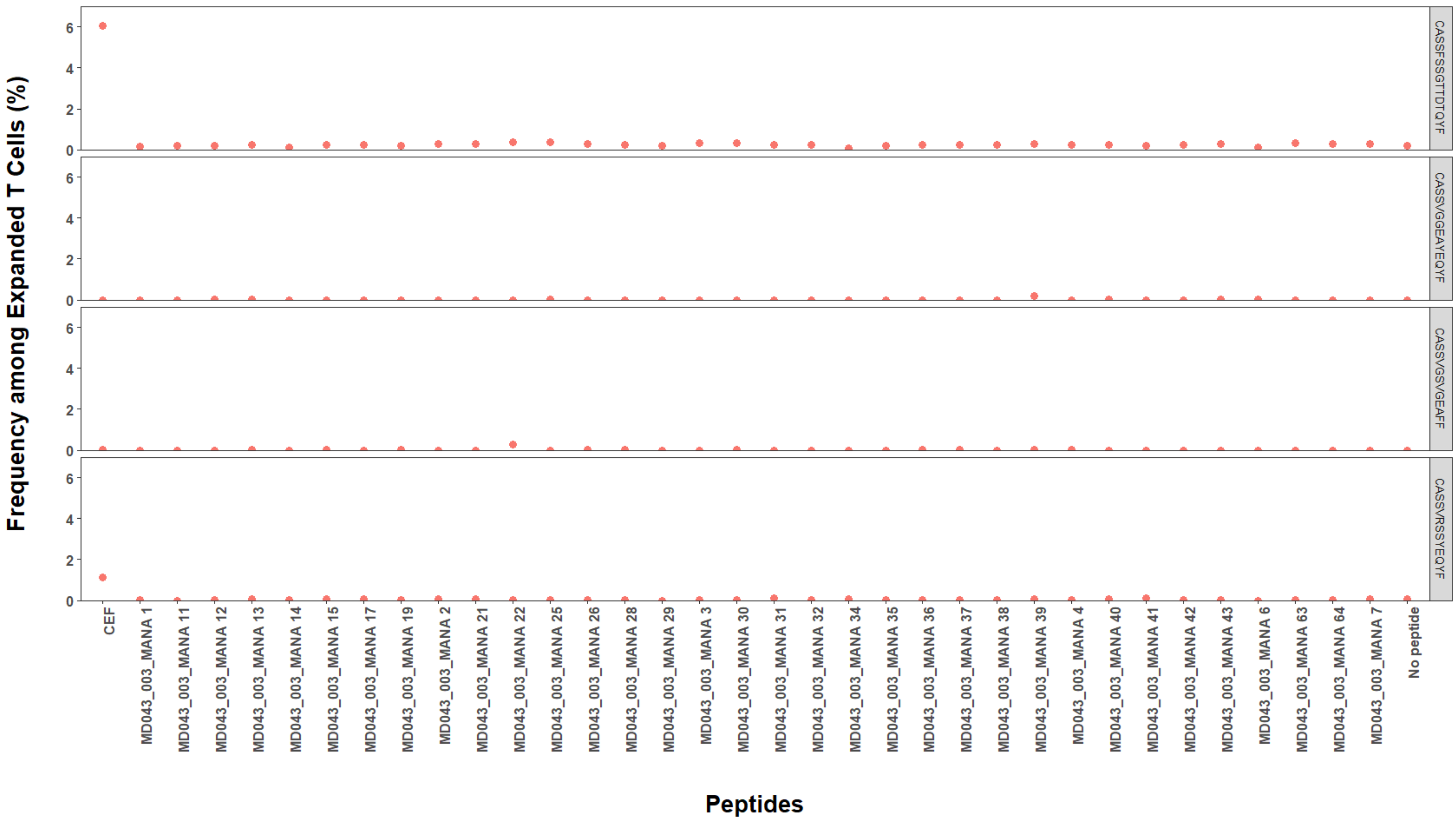

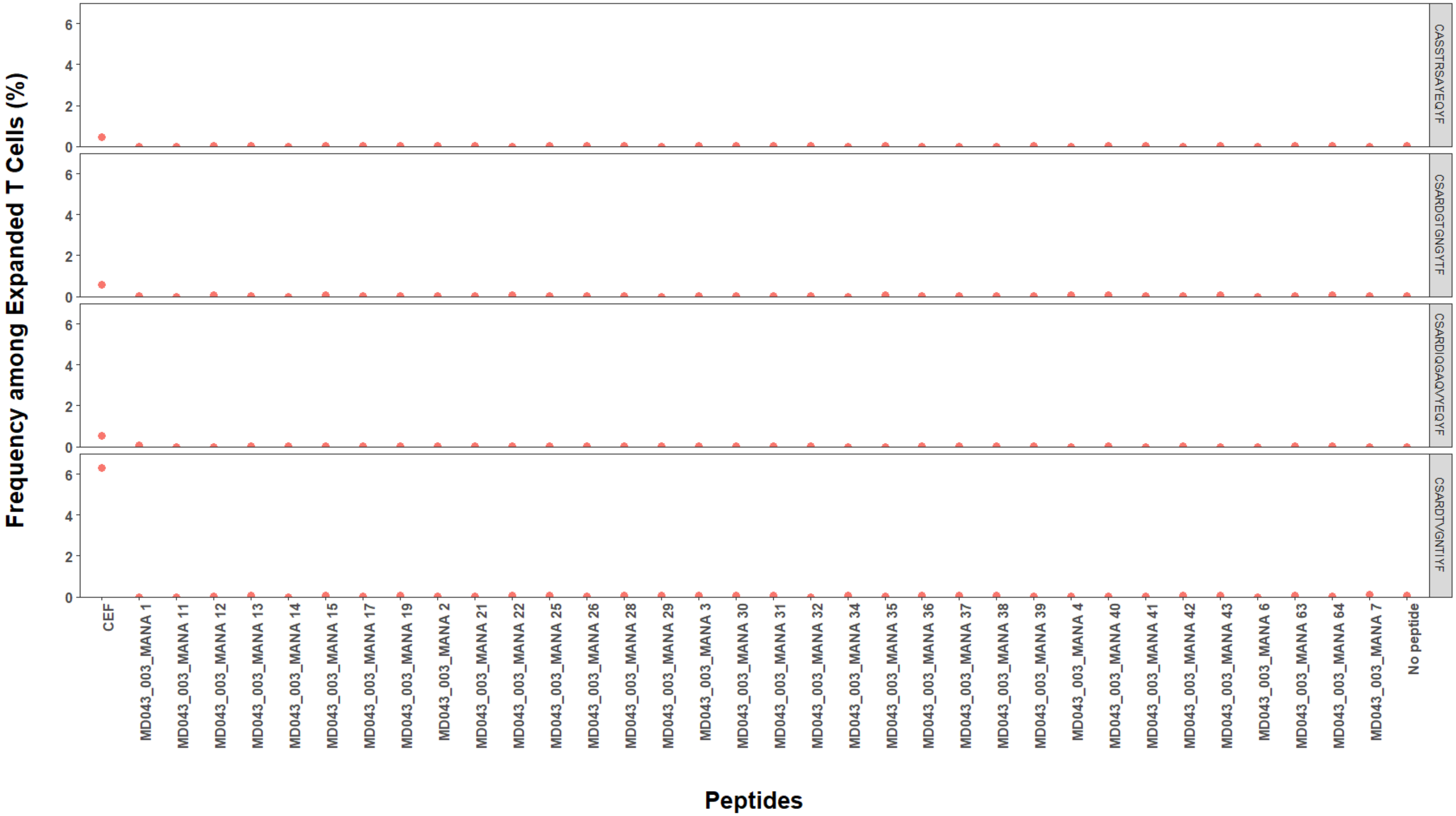
Frequency among Expanded T Cells (%)
CASSTRSAYEQYF
CSARDGTGNGYTF
CSARDIQGAQVYEQYF
CSARDTVGNTIYF
CEF
MD043_003_MANA 1
MD043_003_MANA 11
MD043_003_MANA 12
MD043_003_MANA 13
MD043_003_MANA 14
MD043_003_MANA 15
MD043_003_MANA 17
MD043_003_MANA 19
MD043_003_MANA 2
MD043_003_MANA 21
MD043_003_MANA 22
MD043_003_MANA 25
MD043_003_MANA 26
MD043_003_MANA 28
MD043_003_MANA 29
MD043_003_MANA 3
MD043_003_MANA 30
MD043_003_MANA 31
MD043_003_MANA 32
MD043_003_MANA 34
MD043_003_MANA 35
MD043_003_MANA 36
MD043_003_MANA 37
MD043_003_MANA 38
MD043_003_MANA 39
MD043_003_MANA 4
MD043_003_MANA 40
MD043_003_MANA 41
MD043_003_MANA 42
MD043_003_MANA 43
MD043_003_MANA 6
MD043_003_MANA 63
MD043_003_MANA 64
MD043_003_MANA 7
No peptide
Peptides

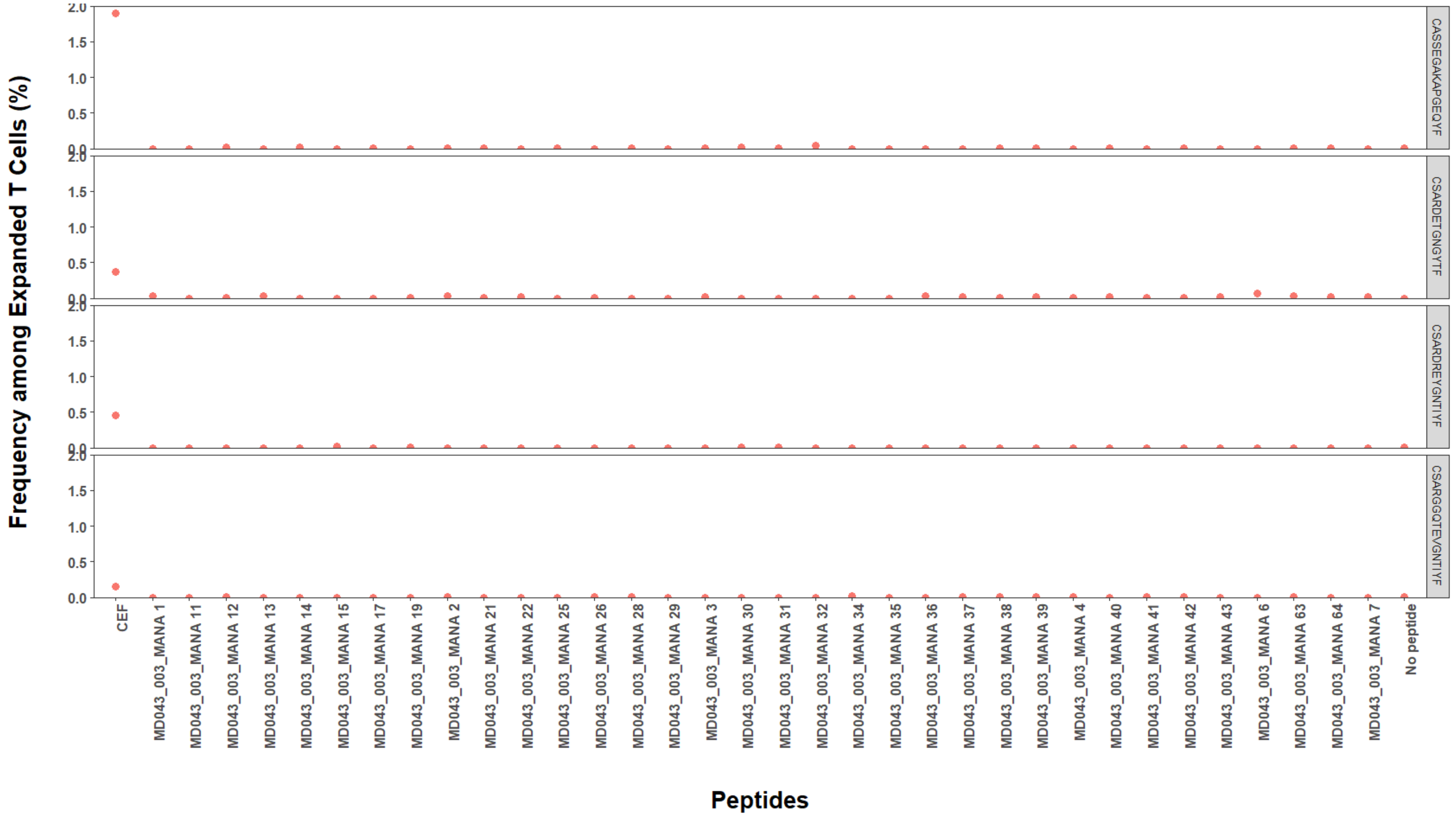
Frequency among Expanded T Cells (%)
2.0
1.5
1.0
0.5
0.0
CASSEGAKAPGEQYF
CSARDETGNGYTF
CSARDREYGNTIYF
CSARGGQTEVGNTIYF
CEF
MD043_003_MANA 1
MD043_003_MANA 11
MD043_003_MANA 12
MD043_003_MANA 13
MD043_003_MANA 14
MD043_003_MANA 15
MD043_003_MANA 17
MD043_003_MANA 19
MD043_003_MANA 2
MD043_003_MANA 21
MD043_003_MANA 22
MD043_003_MANA 25
MD043_003_MANA 26
MD043_003_MANA 28
MD043_003_MANA 29
MD043_003_MANA 3
MD043_003_MANA 30
MD043_003_MANA 31
MD043_003_MANA 32
MD043_003_MANA 34
MD043_003_MANA 35
MD043_003_MANA 36
MD043_003_MANA 37
MD043_003_MANA 38
MD043_003_MANA 39
MD043_003_MANA 4
MD043_003_MANA 40
MD043_003_MANA 41
MD043_003_MANA 42
MD043_003_MANA 43
MD043_003_MANA 6
MD043_003_MANA 63
MD043_003_MANA 64
MD043_003_MANA 7
No peptide
Peptides

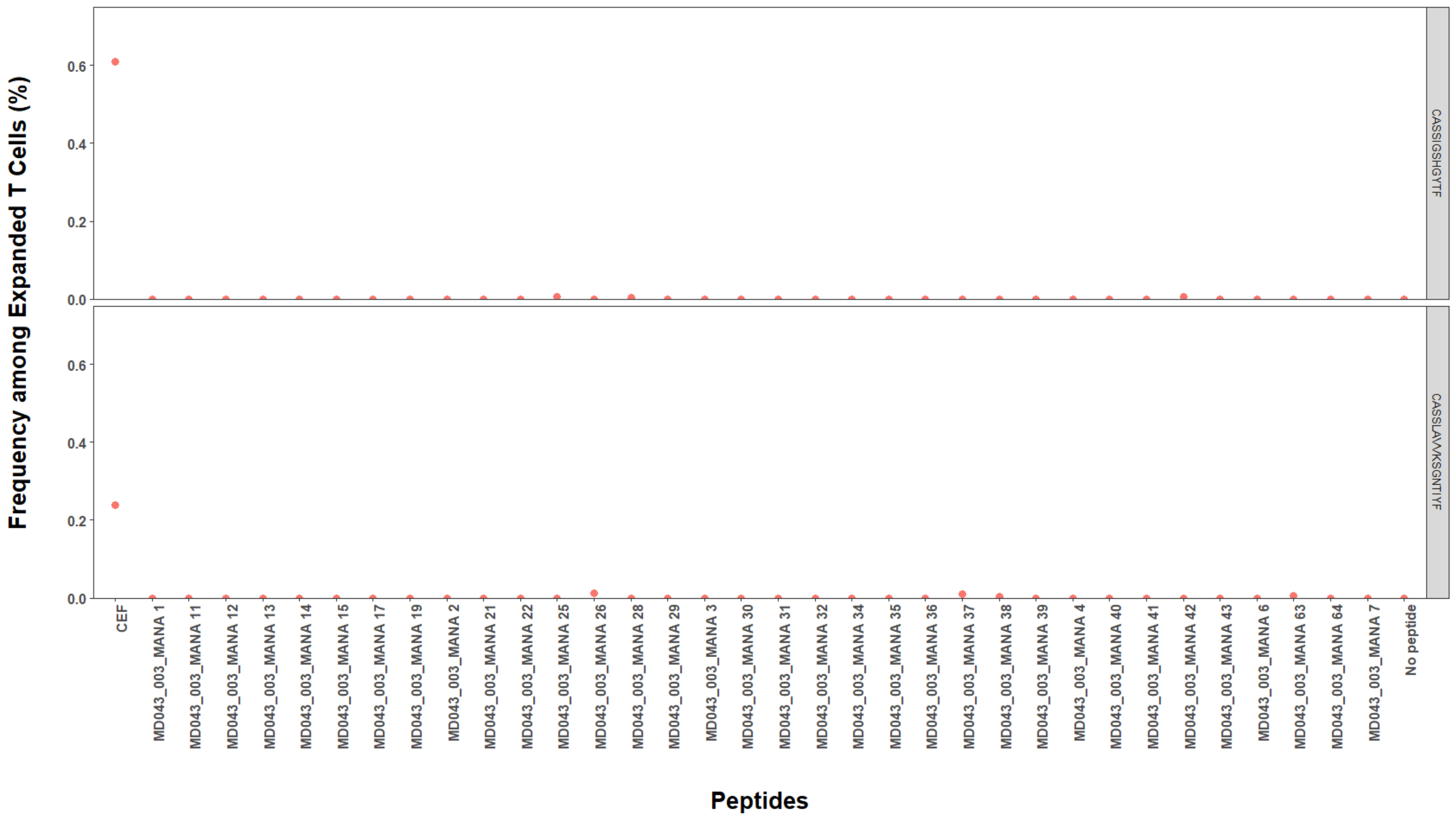
Frequency among Expanded T Cells (%)
0.6
0.4
0.2
0.0
CASSIGSHGYTF
CASSLAVKSGNTIYF
CEF
MD043_003_MANA 1
MD043_003_MANA 11
MD043_003_MANA 12
MD043_003_MANA 13
MD043_003_MANA 14
MD043_003_MANA 15
MD043_003_MANA 17
MD043_003_MANA 19
MD043_003_MANA 2
MD043_003_MANA 21
MD043_003_MANA 22
MD043_003_MANA 25
MD043_003_MANA 26
MD043_003_MANA 28
MD043_003_MANA 29
MD043_003_MANA 3
MD043_003_MANA 30
MD043_003_MANA 31
MD043_003_MANA 32
MD043_003_MANA 34
MD043_003_MANA 35
MD043_003_MANA 36
MD043_003_MANA 37
MD043_003_MANA 38
MD043_003_MANA 39
MD043_003_MANA 4
MD043_003_MANA 40
MD043_003_MANA 41
MD043_003_MANA 42
MD043_003_MANA 43
MD043_003_MANA 6
MD043_003_MANA 63
MD043_003_MANA 64
MD043_003_MANA 7
No peptide
Peptides

Patient MD043-008

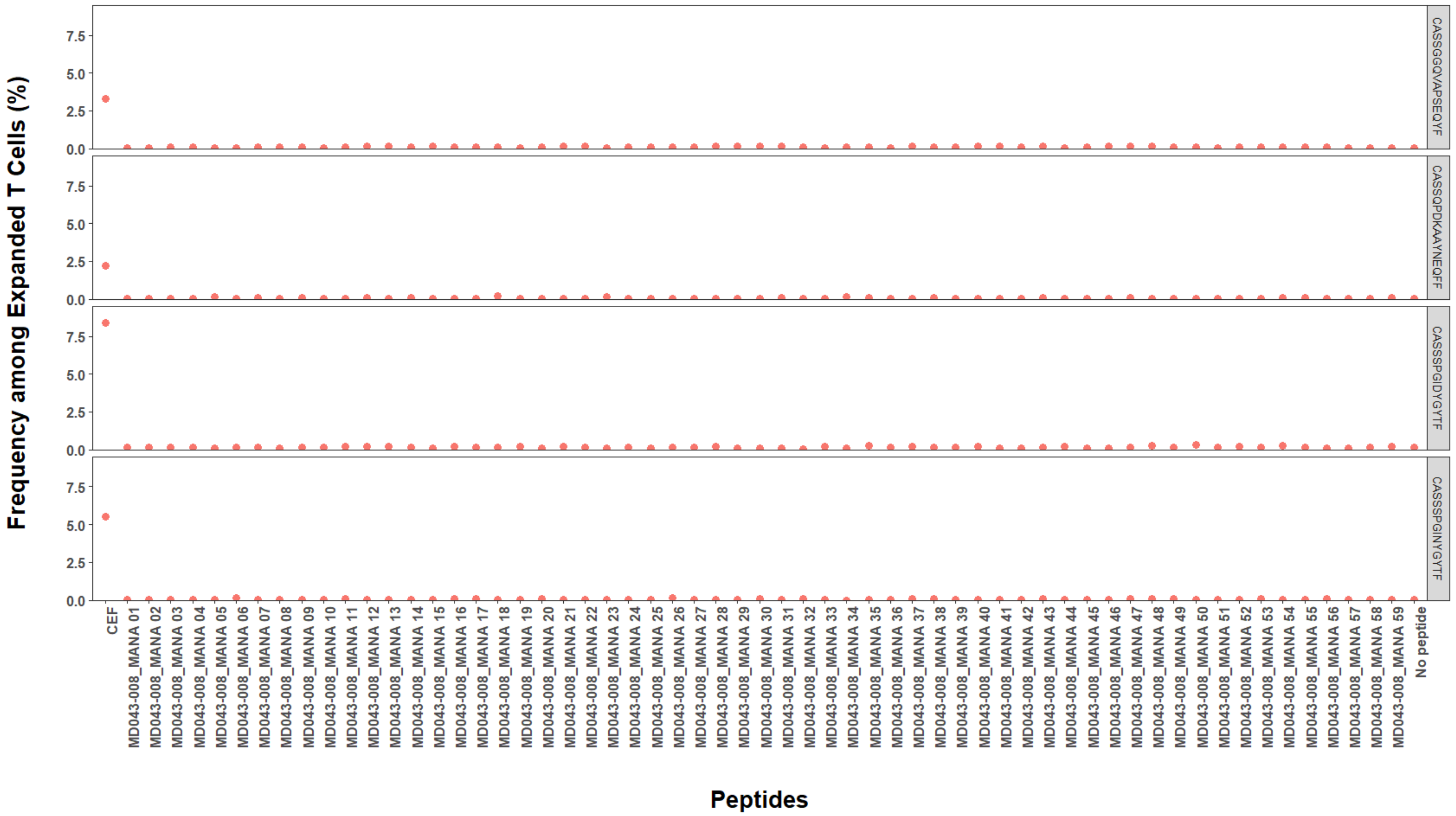

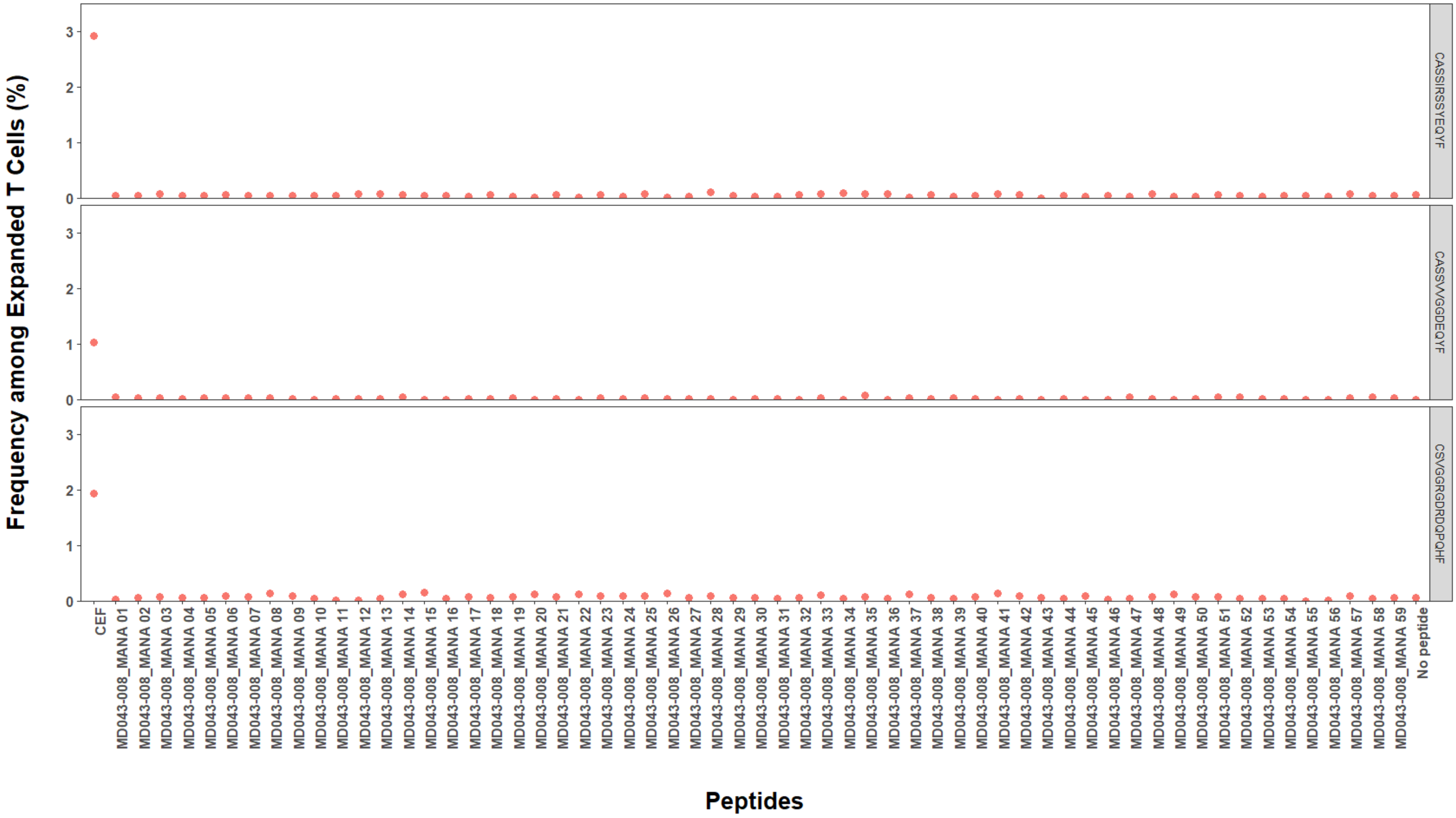

Frequency among Expanded T Cells (%)
CASSIRSSYEQYF
CASSVVGGDEQYF
CSVGGRGRDQPQHF
CEF
MD043-008_MANA 01
MD043-008_MANA 59
No peptide
Peptides

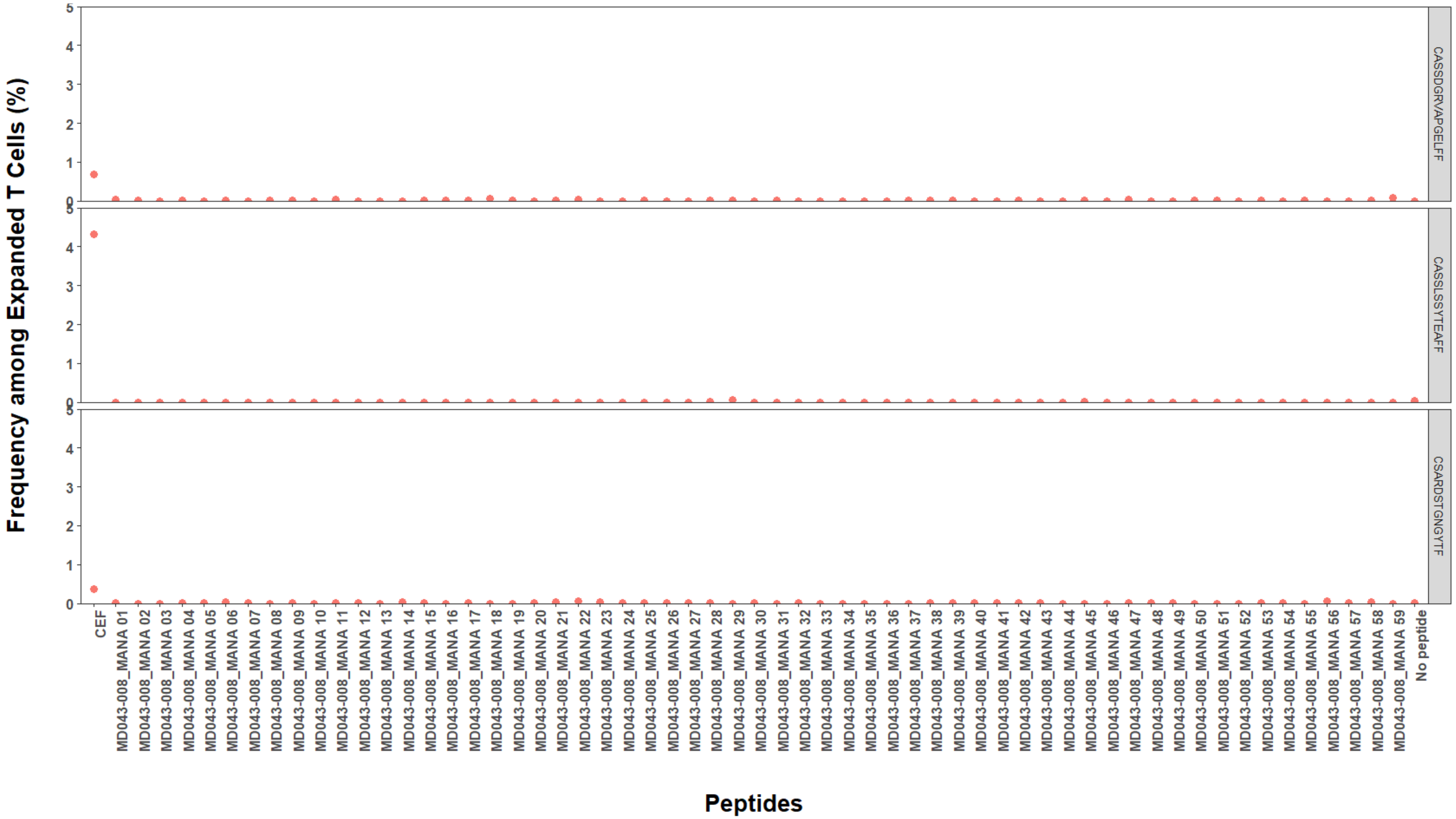
Frequency among Expanded T Cells (%)
Peptides
CASSDGRVAPGELFF
CASSLSSYTEAFF
CSARDSTGNGYTF
CEF
MD043-008_MANA 01 … MD043-008_MANA 59
No peptide

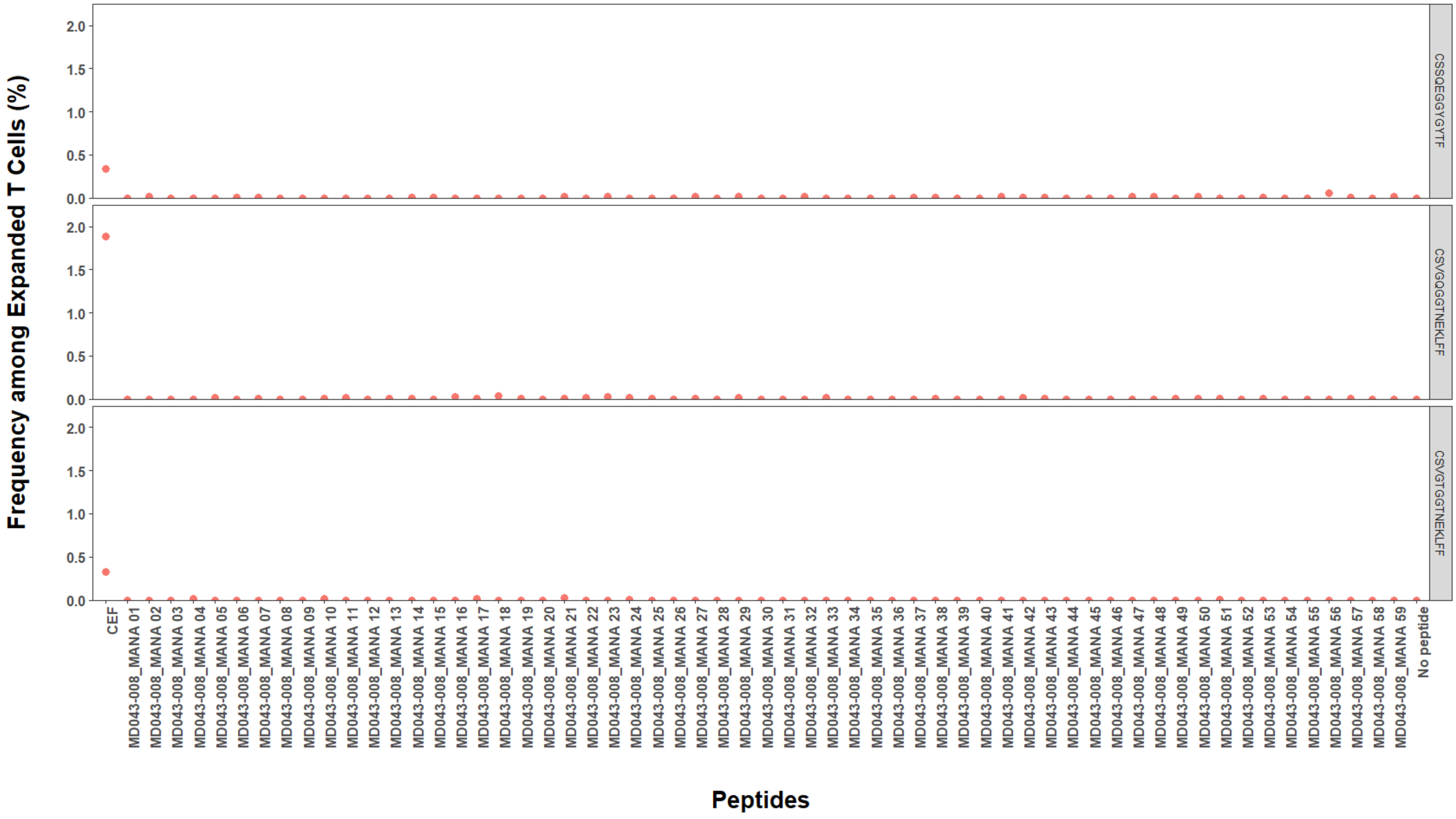
Frequency among Expanded T Cells (%)
Peptides
CSSQEGGYGYTF
CSVGQGGTNEKLFF
CSVGTGGTNEKLFF
0.0
0.5
1.0
1.5
2.0
CEF
MD043-008_MANA 01
MD043-008_MANA 02
MD043-008_MANA 03
MD043-008_MANA 04
MD043-008_MANA 05
MD043-008_MANA 06
MD043-008_MANA 07
MD043-008_MANA 08
MD043-008_MANA 09
MD043-008_MANA 10
MD043-008_MANA 11
MD043-008_MANA 12
MD043-008_MANA 13
MD043-008_MANA 14
MD043-008_MANA 15
MD043-008_MANA 16
MD043-008_MANA 17
MD043-008_MANA 18
MD043-008_MANA 19
MD043-008_MANA 20
MD043-008_MANA 21
MD043-008_MANA 22
MD043-008_MANA 23
MD043-008_MANA 24
MD043-008_MANA 25
MD043-008_MANA 26
MD043-008_MANA 27
MD043-008_MANA 28
MD043-008_MANA 29
MD043-008_MANA 30
MD043-008_MANA 31
MD043-008_MANA 32
MD043-008_MANA 33
MD043-008_MANA 34
MD043-008_MANA 35
MD043-008_MANA 36
MD043-008_MANA 37
MD043-008_MANA 38
MD043-008_MANA 39
MD043-008_MANA 40
MD043-008_MANA 41
MD043-008_MANA 42
MD043-008_MANA 43
MD043-008_MANA 44
MD043-008_MANA 45
MD043-008_MANA 46
MD043-008_MANA 47
MD043-008_MANA 48
MD043-008_MANA 49
MD043-008_MANA 50
MD043-008_MANA 51
MD043-008_MANA 52
MD043-008_MANA 53
MD043-008_MANA 54
MD043-008_MANA 55
MD043-008_MANA 56
MD043-008_MANA 57
MD043-008_MANA 58
MD043-008_MANA 59
No peptide

Patient NY016-007

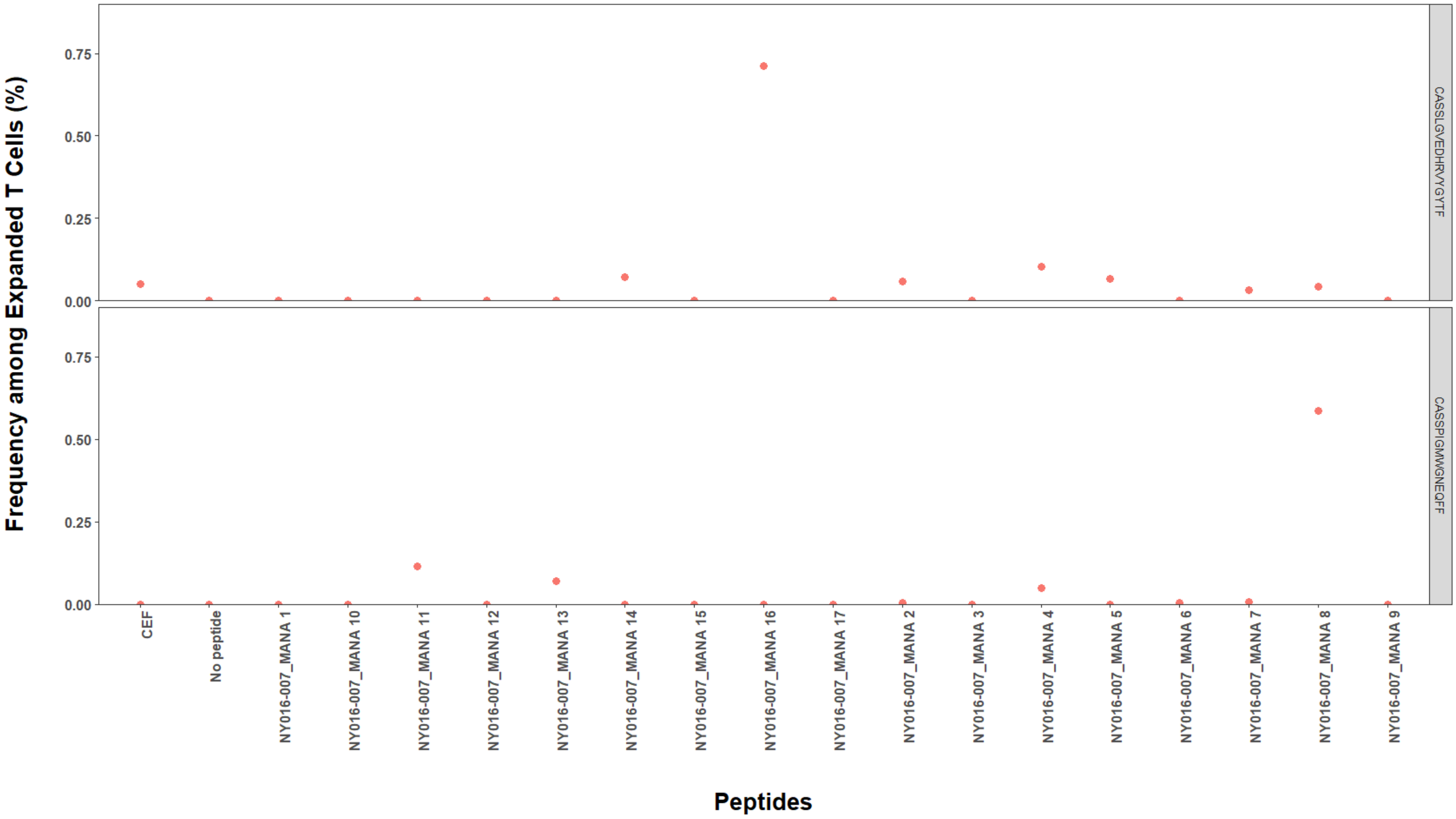

Patient NY016-014

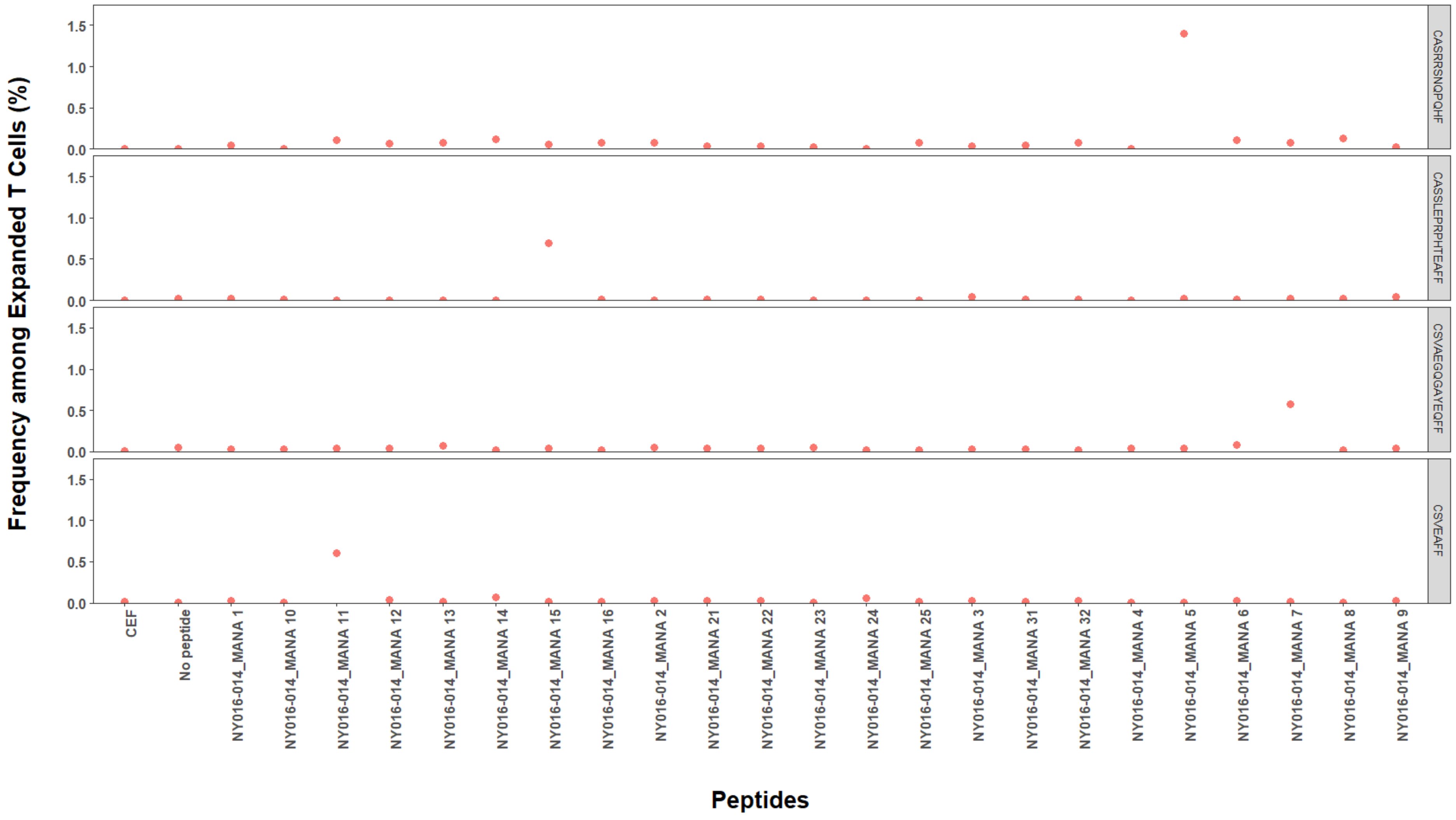

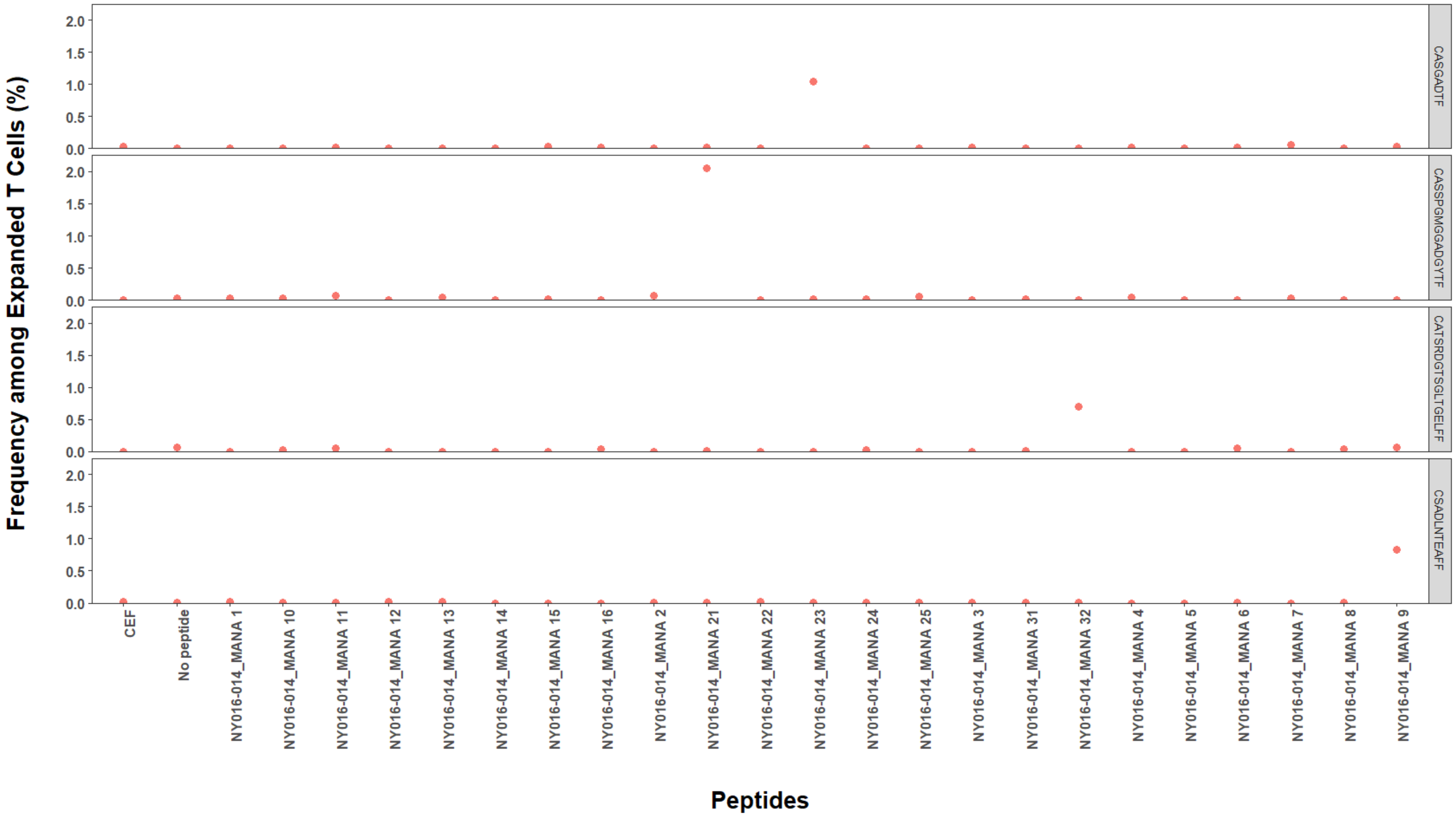

Frequency among Expanded T Cells (%)
Peptides
CASGADTF
CASSPGMGGADGYTF
CATSRDGTSGLTGELFF
CSADLNTEAFF
CEF
No peptide
NY016-014_MANA 1
NY016-014_MANA 10
NY016-014_MANA 11
NY016-014_MANA 12
NY016-014_MANA 13
NY016-014_MANA 14
NY016-014_MANA 15
NY016-014_MANA 16
NY016-014_MANA 2
NY016-014_MANA 21
NY016-014_MANA 22
NY016-014_MANA 23
NY016-014_MANA 24
NY016-014_MANA 25
NY016-014_MANA 3
NY016-014_MANA 31
NY016-014_MANA 32
NY016-014_MANA 4
NY016-014_MANA 5
NY016-014_MANA 6
NY016-014_MANA 7
NY016-014_MANA 8
NY016-014_MANA 9

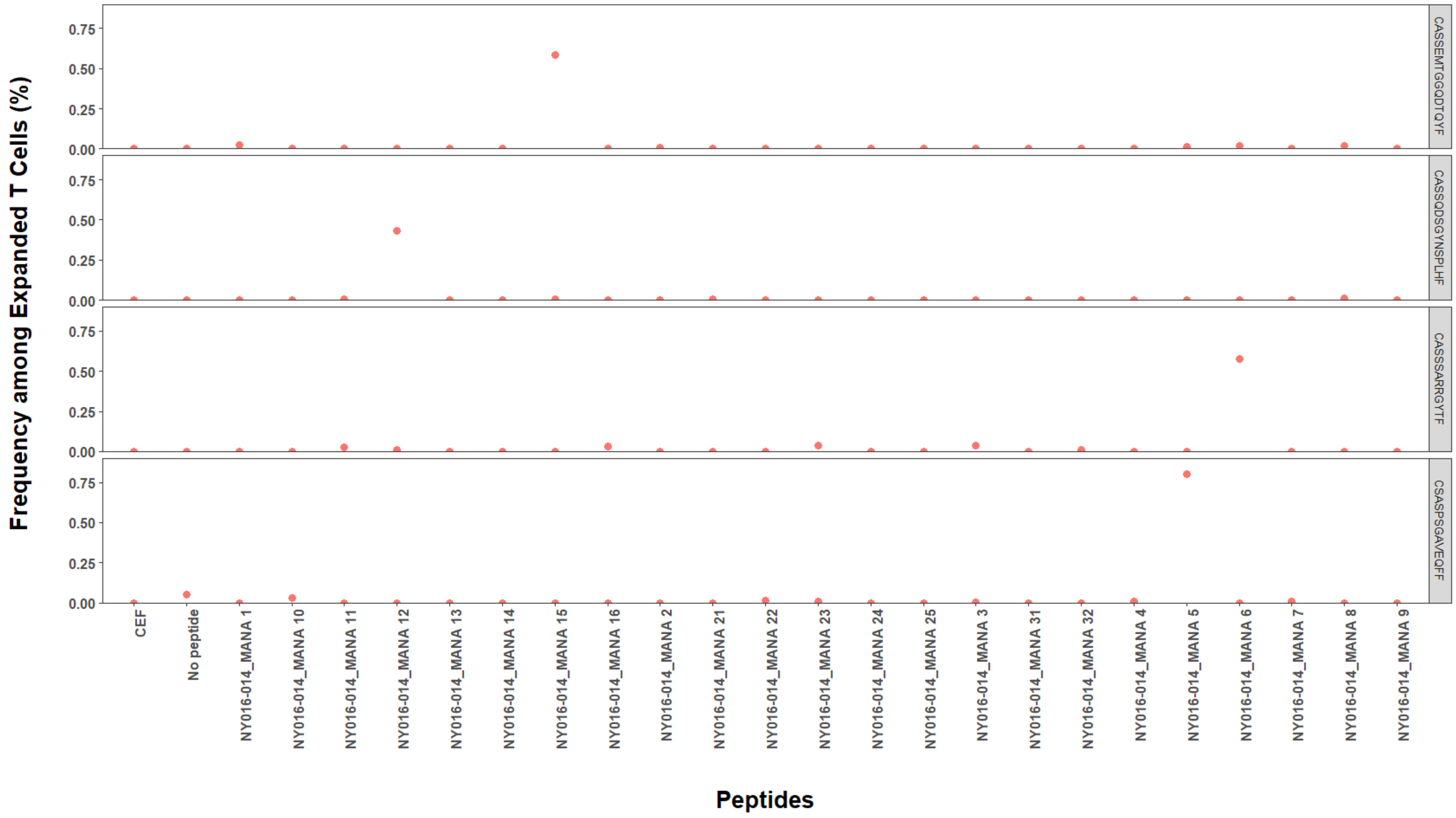

Frequency among Expanded T Cells (%)
0.75
0.50
0.25
0.00
CASSEMTGGDTQYF
CASSQDSGYNSPLHF
CASSSARRGYTF
CSASPSGAVEQFF
CEF
No peptide
NY016-014_MANA 1
NY016-014_MANA 10
NY016-014_MANA 11
NY016-014_MANA 12
NY016-014_MANA 13
NY016-014_MANA 14
NY016-014_MANA 15
NY016-014_MANA 16
NY016-014_MANA 2
NY016-014_MANA 21
NY016-014_MANA 22
NY016-014_MANA 23
NY016-014_MANA 24
NY016-014_MANA 25
NY016-014_MANA 3
NY016-014_MANA 31
NY016-014_MANA 32
NY016-014_MANA 4
NY016-014_MANA 5
NY016-014_MANA 6
NY016-014_MANA 7
NY016-014_MANA 8
NY016-014_MANA 9
Peptides

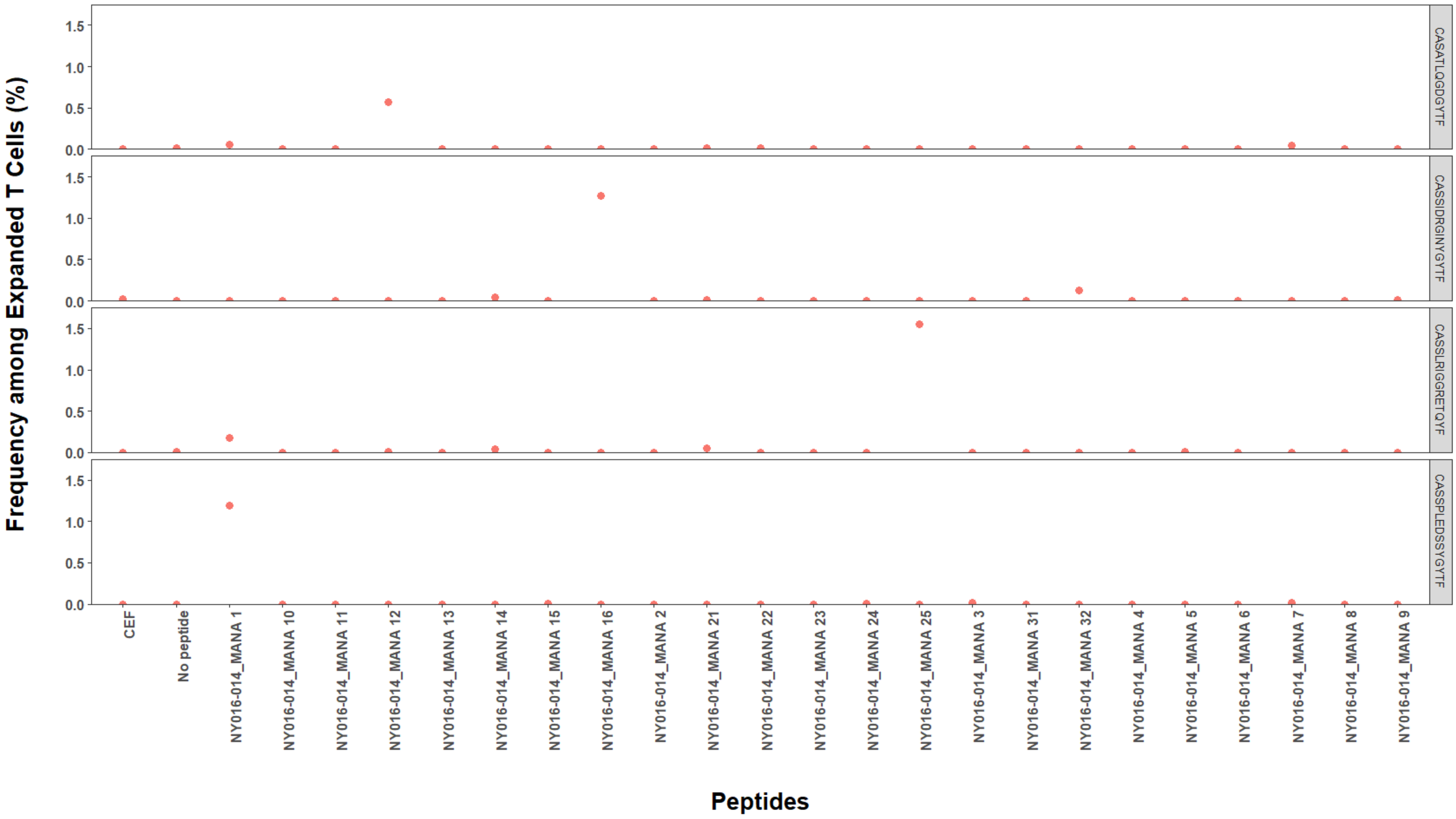

Frequency among Expanded T Cells (%)
0.0
0.5
1.0
1.5
CASATLQGDGYTF
CASSIDRGINYGYTF
CASSLRIGGRETQYF
CASSPLEDSSYGYTF
CEF
No peptide
NY016-014_MANA 1
NY016-014_MANA 10
NY016-014_MANA 11
NY016-014_MANA 12
NY016-014_MANA 13
NY016-014_MANA 14
NY016-014_MANA 15
NY016-014_MANA 16
NY016-014_MANA 2
NY016-014_MANA 21
NY016-014_MANA 22
NY016-014_MANA 23
NY016-014_MANA 24
NY016-014_MANA 25
NY016-014_MANA 3
NY016-014_MANA 31
NY016-014_MANA 32
NY016-014_MANA 4
NY016-014_MANA 5
NY016-014_MANA 6
NY016-014_MANA 7
NY016-014_MANA 8
NY016-014_MANA 9
Peptides

Patient NY016-025

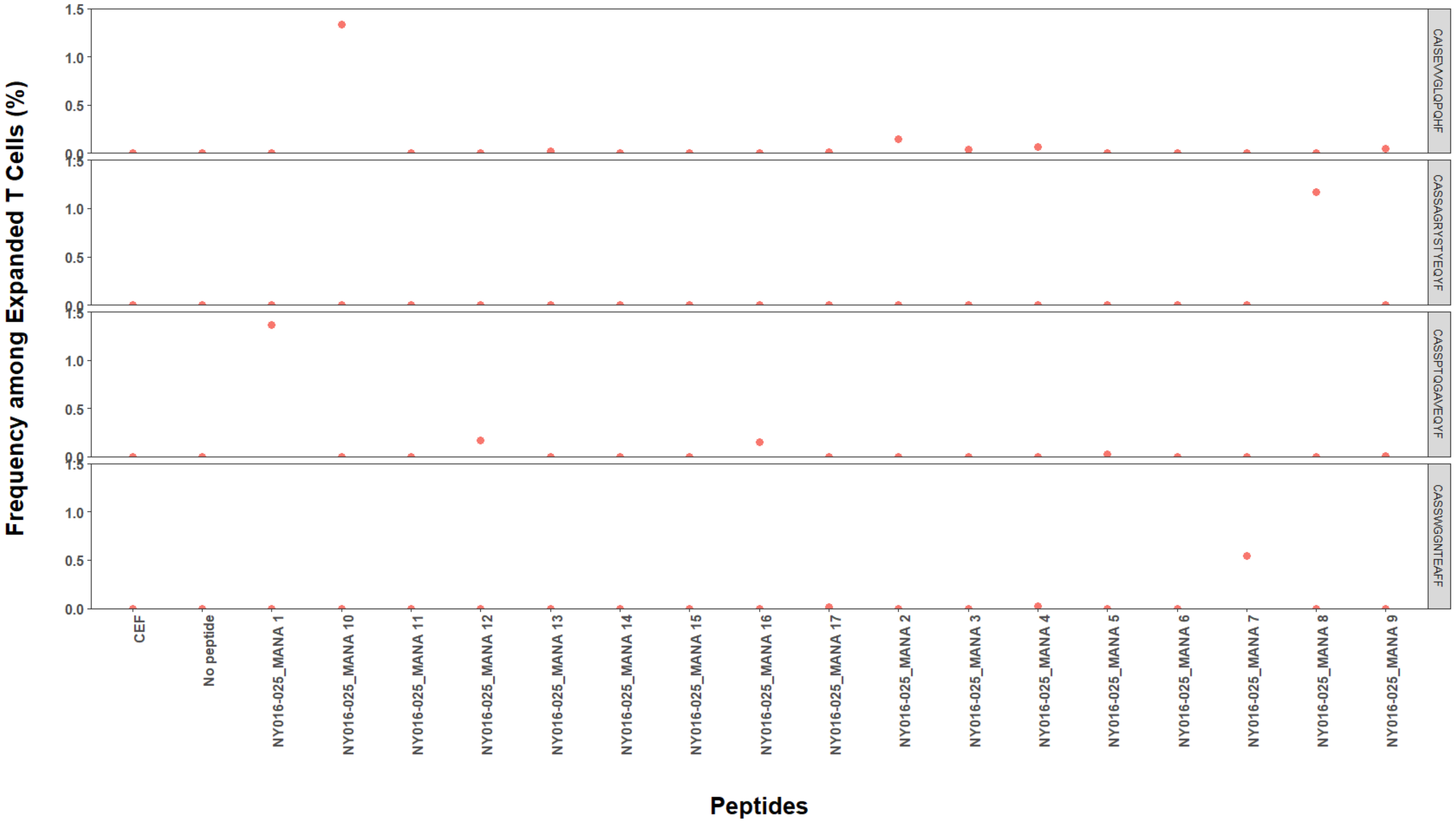

Supplement: Supplementary file 7 — This file contains MANAFEST and ViraFEST assay graphs. See Supplementary Information PDF for full file description. [file 41586_2021_3752_MOESM7_ESM.pdf]
